# Supplementary material for: Impact of rapid near-patient STI testing on service delivery outcomes in an integrated sexual health service in the United Kingdom: a controlled interrupted time series study
Source: BMJ Open. 2023 Jan 11;13(1):e064664. doi: 10.1136/bmjopen-2022-064664 (PMC9835959; doi:10.1136/bmjopen-2022-064664)
Supplement: Supplementary data [file bmjopen-2022-064664supp001.pdf]

Supplementary material

The impact of rapid near-patient STI testing on service delivery outcomes in an integrated sexual health service in the United Kingdom: a controlled interrupted time series study

Scott R Walter, Joni Jackson, Gareth Myring, et al.

Contents:

Rapid STI testing details

Figure S1. Male treatment pathway before (usual care pathway) and after (rapid pathway) implementation of the Panther rapid results system.

Figure S2. Overview of female rapid treatment pathway for asymptomatic and symptomatic patients before (usual care pathway) and after (rapid pathway) implementation of the Panther rapid results system.

Table S1. Definition of examination of any type based on a combination of two examination-related variables.

Figure S3. Diagrams to illustrate controlled interrupted time series variables for estimating A) changes at the time of intervention and B) changes in trends.

Table S2. Unit costs.

Table S3. Intervention-related model estimates for females and males from sensitivity analyses using generalised additive models.

Figure S4. Modelled outcome estimates for males based on sensitivity analyses using generalised additive models.

Figure S5. Modelled outcome estimates for females based on sensitivity analyses using generalised additive models.

Figure S6. Modelled estimates of staff capacity for males and females combined.

Definition of complex cases

Definition of ethnic minority

Rapid STI testing details

Male urine samples, self-taken vaginal swabs, and clinician-taken endocervical swabs, rectal swabs and throat swabs were collected into the appropriate Aptima Collection kits (Hologic) and tested in the satellite laboratory at the intervention site for chlamydia and gonorrhoea using the Aptima Combo 2 assay (Hologic). Vaginal and endocervical samples were tested for Trichomonas using the Aptima *Trichomonas vaginalis* assay (Hologic). Reactive chlamydia NAAT results were not confirmed and were reported as positive, as internal audit has demonstrated that the result of the Aptima Combo 2 NAAT has a high concordance with Aptima CT NAAT when used to confirm a reactive chlamydia Aptima Combo 2 result[S1].

A reactive gonorrhoea Aptima Combo 2 result was reported as reactive and sent to the main laboratory (UKHSA South West Regional Laboratory) for confirmation using the Aptima GC NAAT. As the UKHSA South West Regional Laboratory have a > 90% positive predictive value (low risk of false positives) for a reactive gonorrhoea Aptima Combo 2 result, such patients were managed by clinicians as though they were gonorrhoea-positive which is consistent with the British Association for Sexual Health and HIV (BASHH) national guideline[S2] but informed that there was a small chance it could be a false positive result pending the confirmatory test result, which was usually available within 2 working days. All assays were performed using the automated Panther instrument (Hologic). Quality control measures in both laboratories included weekly testing of the Amplirun Total CT/NG/TV/MGE Control (Vircell, testing of NEQAS *Chlamydia trachomatis* & *Neisseria gonorrhoeae* external quality assessment (EQA) panels three times per annum and testing of Quality Control in Molecular Diagnostics *C. trachomatis* DNA and *N. gonorrhoeae* DNA EQA panels twice per annum.)

S1. Public Health England. UK Standards for Microbiology Investigations: *Chlamydia trachomatis* infection – testing by Nucleic Acid Amplification Tests (NAAT). V 37, issue 4, 9th January 2017. Available at: https://assets.publishing.service.gov.uk/government/uploads/system/uploads/attachment_data/file/583847/V_37i4.pdf)

S2. Fifer H, Saunders J, Soni S, Sadiq ST, FitzGerald M. UK national guideline for the management of infection with Neisseria gonorrhoeae. Int J STD AIDS, **2020**; 31(1): 4-15. doi: 10.1177/0956462419886775.

Figure S1. Male treatment pathway before (usual care pathway) and after (rapid pathway) implementation of the Panther rapid results system. Reproduced from: Lorenc A, Kesten J, Brangan E, Horner PJ, Clarke M, Crofts M, Turner J, Muir P, Horwood J. What can be learnt from a qualitative evaluation of implementing a rapid sexual health testing, diagnosis and treatment service? BMJ Open, 2021; 11: e050109. doi: 10.1136/bmjopen-2021-050109.

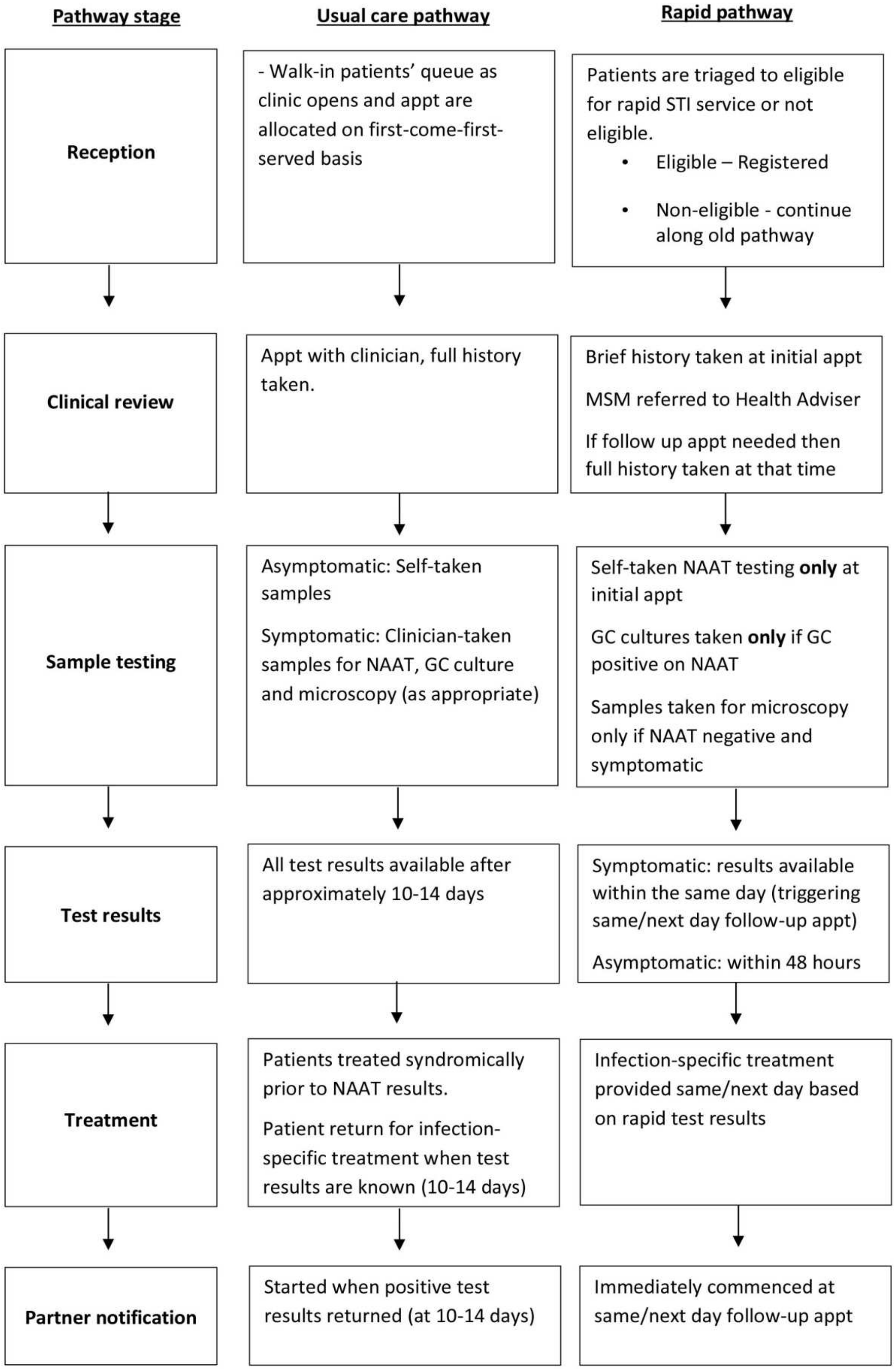

Figure S2. Overview of female rapid treatment pathway for asymptomatic and symptomatic patients before (usual care pathway) and after (rapid pathway) implementation of the Panther rapid results system. NAAT = Nucleic Acid Amplification Test GC = Gonorrhoea CT= Chlamydia, TV= *Trichomonas vaginalis*

Pathway stage	Usual care pathway	Rapid pathway: asymptomatic	Rapid pathway: symptomatic
**Reception**	Walk-in patients' queue as clinic opens and appointments are allocated on first-come-first-served basis.	Patients are triaged to rapid STI service or not:<br>● Asymptomatic and Eligible = Registered<br>● Asymptomatic and Non-eligible = continue along modified usual care pathway	Patients triaged to rapid STI service or not:<br>● Symptomatic and Eligible = Registered<br>● Symptomatic and non-eligible = modified usual care pathway (GC/CT and TV NAAT results <48 hrs) GC cultures only taken if GC NAAT positive or patient receives treatment for GC before results of GC NAAT available
**Clinical review**	Appt. with clinician, full history taken.	Brief history taken at initial appt.<br>If follow up appt. needed then full history taken at that time	History taken and no indications for visual inspection or pelvic examination required.<br>If follow up appt. needed then full history taken at that time
**Sample testing**	● Asymptomatic: Self-taken samples<br>● Symptomatic: Clinician-taken samples for CT/GC NAAT. If vaginal symptoms or pelvic pain then speculum exam and swabs taken for microscopy, TV and GC culture (as appropriate).	Self-taken NAAT testing ***only*** at initial appt<br>GC cultures taken ***only*** if GC positive on NAAT or if treatment as GC contact within 2 weeks of sexual intercourse with contact	Self-taken:<br>● Vaginal CT/GC/TV NAAT<br>● Vaginal swab for Gram staining<br>GC cultures taken ***only*** if GC positive on NAAT or if treatment as GC contact within 2 weeks of sexual intercourse with contact.
**Test results**	All test results available after approximately 10-14 days.	Results available within 48 hours.	Results of vaginal microscopy available in 20 mins and patient is then reviewed by clinician CT/GCTV NAAT results available within 48 hours
**Treatment**	Patients treated syndromically prior to NAAT results.<br>Patients return for infection-specific treatment when test results are known (10-14 days).	Infection-specific treatment provided same/next day based on rapid test results.	Infection-specific treatment provided same/next day based on rapid test results.
**Partner notification**	Started when positive test results returned (at 10-14 days).	Immediately commenced at same/next day follow-up appt.	Immediately commenced at same/next day follow-up appt.

Table S1. Definition of examination of any type based on a combination of two examination-related variables.

Categorical exam variable	Free text exam variable	Define as exam – MALES	Define as exam - FEMALES
Yes	Notes indicating exam	Yes	Yes
Yes	Missing	Yes	Yes
No	Notes indicating exam	Yes	Yes
No	Notes indication NO exam		
No	Missing		
External only	Notes indicating exam	Yes	Yes
External only	Notes indication NO exam		
External only	Missing		Yes
Speculum and external	Notes indicating exam	Yes	Yes
Speculum and external	Notes indication NO exam		
Speculum and external	Missing		Yes
Missing	Notes indicating exam	Yes	Yes
Missing	Notes indication NO exam		
Missing	Missing		

Notes: The categorical exam variable was intended for use with female patients but was sometimes used for males.

Figure S3. Diagrams to illustrate controlled interrupted time series variables for estimating A) changes at the time of intervention and B) changes in trends.

A. Changes at the time of intervention

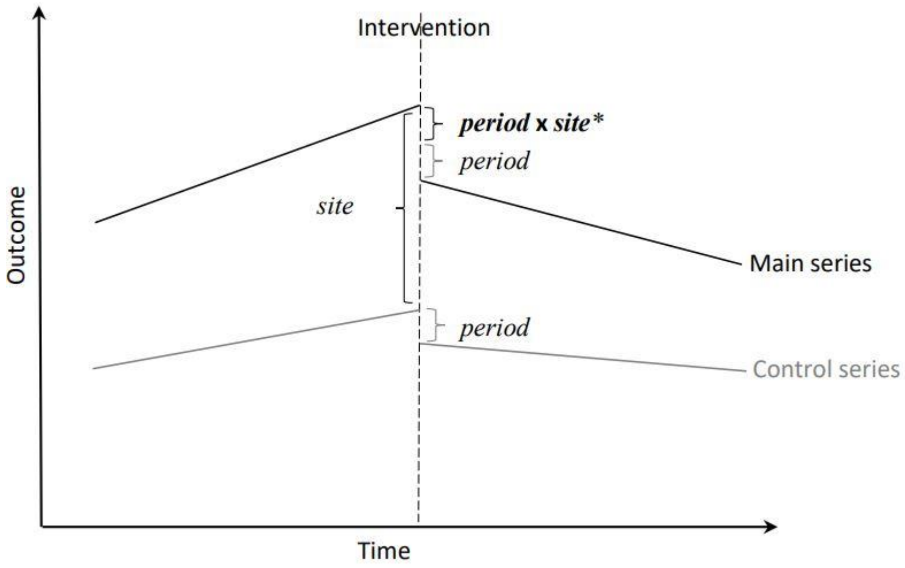

B. Trend changes

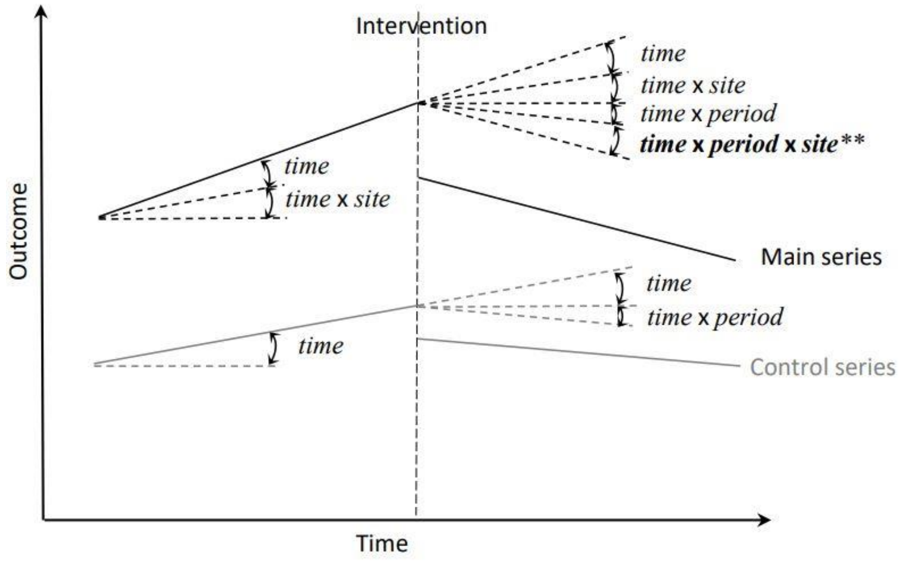

Note: *site* is a binary variable indicating either intervention or control sites; *period* is also binary indicating pre- or post-intervention periods; *time* is a continuous variable consecutively numbering each time unit (weeks in this study) with *time*=0 centred at the intervention

* *period* x *site* represents change in the intervention site at the time of intervention over and above any changes in the control site

** *time* x *period* x *site* represents change in trend for the intervention site over and above any trend changes in the control site

Table S2. Unit costs.

Unit costs of postal kit tests, and staff time[12] were from the literature and inflated to 2021 values using a UK government GDP deflator[26]. Local unit costs of diagnostic tests were provided by the intervention site. Treatment costs were from the British National Formulary[27].

Resource use	Unit cost
**Tests**	
Lab CT/GC test	£8.10
POCT CT/GC	£9.48
GC culture swab	£6.13
Male postal kit returned	£4.44
Male postal kit not returned	£3.61
Female postal kit returned	£4.08
Female postal kit not returned	£3.24
**Consultation staff time**	
Follow-up	£9.33
Male non-complex	£29.03
Male complex	£46.54
MSM complex	£42.97
Female non-complex	£29.03
Female complex	£52.26
**Treatment**	
13.5mg Levonorgestrel IUS	£69.22
6mg norelgestromin and 600micrograms ethinylestradiol	£19.51
Aciclovir 400 mg (tds for 5 Days)	£0.79
Aciclovir 400mg (bd for 6 months)	£17.64
Aciclovir 400mg (bd for 3 months)	£8.82
Aciclovir 800mg (tds for 2 days)	£0.59
Amoxicillin 250 mg tds for 5 days	£0.98
Amoxicillin 500mg	£1.01
Anusol Cream	£2.49
Anusol Ointment	£2.49
Anusol Suppositories	£1.74
Aqueous Cream BP 100g Tube	£0.77
Aqueous Cream BP 500g Tub	£3.85
Azithromycin 1g (2 x 500mg tablets)	£0.81
Azithromycin 1g (4 x 250mg capsules)	£1.24
Azithromycin 1g stat, then 500mg od for 2 days	£1.21
Azithromycin 1g stat, then 500mg od for 4 days	£2.42
Azithromycin 2g o stat	£1.62
Benzathine Benzylpenicillin 2.4 million units on day 0	£9.50
Benzathine benzylpenicillin 2.4 million units at day 7	£9.50
Benzathine benzylpenicillin 2.4 million units at day14	£9.50
Betamethasone Valerate 0.1% w/w Cream	£1.47
Betamethasone Valerate 0.1% w/w Ointment	£1.84
Betamethasone Valerate Ointment (Betnovate RD)	£1.84
Cefixime 400mg (2 x 200mg)	£26.46
Ceftriaxone 1g	£3.62
Ceftriaxone 500 mg (2 x 250mg vials)	£4.60
Chlorphenamine	£2.21
Cilest 63 tablet pack	£4.65

Ciprofloxacin 500mg (2 x 250mg)	£0.31
Clindamycin 300mg bd for 7 days	£17.84
Clindamycin phosphate vaginal cream	£10.86
Clobetasol Propinate (0.05% w/w) Cream (Dermovate)	£2.69
Clobetasol Propionate (0.05% w/w) Ointment	£2.69
Clobetasone Butyrate Cream (Eumovate)	£1.86
Clobetasone Butyrate Ointment (Eumovate)	£1.86
Clobetasone Butyrate, Calcium oxtertracycline & Nystatin Cream (Trimovate)	£12.45
Clotrimazole 100mg Pessary	£0.64
Clotrimazole 200mg Pessary	£1.14
Clotrimazole 500 mg Pessary	£6.99
Clotrimazole Cream 1%	£1.36
Co-Amoxiclav 250/125 (contains PENICILLIN)	£2.03
Co-amxoxiclav 500/125 (contains PENICILLIN)	£2.53
Crotamiton 10% w/w cream	£2.50
Dermol Lotion 500	£6.04
Desogestrel 75 micrograms	£2.26
Doxycycline 100mg (bd for 14 days)	£3.67
Doxycycline 100mg (bd for 21 days)	£5.51
Doxycycline 100mg (bd for 28 days)	£7.35
Doxycycline 100mg (bd for 7 days)	£2.26
Doxycycline 200mg bd for 4 weeks	£5.51
Emtricitabine 200mg & Tenofovir Disproxil 245mg	£106.00
Emtricitabine 200mg & Tenofovir Disproxil 245mg (3 days)	£10.60
Emulsifying Ointment	£4.82
Erythromycin 250 mg	£8.95
Estradiol 0.5g gel	£5.08
Estradiol 1.0mg gel	£5.85
Estradiol 10 micrograms vaginal tablet	£16.72
Femodene 63 tablet pack	£6.73
Flucloxacillin	£1.41
Fluconazole 150mg	£0.91
Fusidic acid cream	£1.92
GENTAMICIN 240mg for IM injection	£4.13
GYNAEFIX IUD	£27.11
Gardasil 0.5ml - First Dose	£86.50
Gardasil 0.5mls - Second Dose	£86.50
Gardasil 0.5mls - Third Dose	£86.50
Gedarel 20/150	£5.08
Gedarel 30/150	£5.08
Hepatitis A & B Combined Vaccine (adult)	£31.18
Hepatitis A Vaccine (2nd at 6 months)	£16.77
Hepatitis A vaccine Day 0	£16.77
Hepatitis B Vaccine (final at 6 months)	£12.20
Hepatitis B Vaccine - Dose 1 - 10mcg or 20mcg	£12.20
Hepatitis B Vaccine - Dose 2 - 10mcg or 20mcg	£12.20
Hepatitis B Vaccine - Dose 3 - 10mcg or 20mcg	£12.20
Hepatitis B Vaccine - Dose 4 - 10mcg or 20mcg	£12.20
Hepatitis B Vaccine - Extra Dose - 10mcg or 20 mcg	£12.20
Hepatitis B Vaccine 10 mcg 1 month	£12.20

Hepatitis B Vaccine 10 mcg 12 months	£12.20
Hepatitis B Vaccine Day 7	£12.20
Hepatits B Vaccine Day 21	£12.20
Hydro-Caine 6mls	£10.50
Hydrocortisone Cream 1%	£1.40
Hydrocortisone Ointment 1%	£1.59
Ibuprofen 200mg	£1.03
Imiquimod 5%	£48.60
Itraconazole 100mg	£3.29
Levonorgestral and Ethinylestradiol 150microgram/30microgram	£2.60
Levonorgestrel 1.5 mg	£3.65
Levonorgestrel 30 micrograms	£0.92
Levosert 52mgs IUS	£66.00
Lidocaine 4% w/w cream	£2.98
Lidocaine 5% m/m Ointment	£8.28
Lidocaine HCL 1% in 2 mls injection	£0.25
Lidocaine HCL 1% in 3.5 mls injection	£0.30
Lidocaine HCL 1% in 5 mls injection	£0.30
Lidocaine HCL 1% in 8mls for IM inj (with IM penicillin) second dose	£0.10
Lidocaine HCL 1% in 8mls for IM injection (with IM penicillin for syphilis)	£0.10
Lidocaine HCL 1% in 8mls for IM injection (with IM penicillin) third dose	£0.10
Lidocaine HCL 2% in 2 mls injection	£0.27
Lidocaine HCL 2% in 5 mls injection	£0.32
Lignocaine 2% Gel	£2.99
Loestrin 20 63 Tablet Pack	£1.99
Loestrin 30 63 Tablet Pack	£1.99
Logynon	£2.60
Marvelon 63 Tablet Pack	£7.10
Mebendazole 100mg	£2.66
Medroxyprogesterone Acetate 104mg in 0.65mls sub cutaneous	£6.90
Medroxyprogesterone Acetate 150mg in 1ml	£6.01
Mefenamic Acid 250mg	£8.17
Mepivacaine Hydrochloride 3%	£0.44
Mepivicaine 3% in 2.2mls	£0.44
Mercilon 63 Tablet Pack	£8.44
Metronidazole 0.75% Vaginal Gel	£4.31
Metronidazole 2g stat dose (400 mg x 5)	£0.52
Metronidazole 400mg (bd for 5 days)	£1.03
Metronidazole 400mg bd for 10 days	£2.07
Miconazole Nitrate 2%w/w, hydrocortisone 1%w/w Cream (Daktocourt)	£2.49
Miconazole Nitrate Cream 20mg/g (Gyno-Daktarin)	£4.33
Miconazole nitrate 20mg per g	£4.33
Millinette 20/75	£5.41
Millinette 30/75	£4.12
Mini TT 380	£12.46
Mirena 52mg IUS	£88.00
Moxifloxacin 400mg od for 10 days	£19.08
Moxifloxacin 400mg od for 14 days	£26.71
Nexplanon 68mg implant	£83.43
Nitrofurantoin 50mg o qds 7 days	£5.08
Nitrofurantoin 50mg o qds for 3 days	£2.18

Nonoxinol-9	£11.00
Norethisterone 350 micrograms	£2.10
Norethisterone 350 micrograms 84 Tablet Pack	£2.10
Norimin 63 Tablet Pack	£2.28
Nova T 380	£15.20
Ofloxacin 200mg (one tablet twice daily for 14 days)	£12.54
Ofloxacin 200mg (one tablet twice daily for 7 days)	£6.27
Ofloxacin 200mg (two tablets twice daily for 14 days)	£25.09
Paediatric Hepatitis B Vaccine - Dose 1 - 10mcg	£12.20
Paediatric Hepatitis B Vaccine - Dose 2 - 10 mcg	£12.20
Paediatric Hepatitis B Vaccine - Dose 3 - 10 mcg	£12.20
Paediatric Hepatitis B Vaccine - Dose 4 - 10mcg	£12.20
Paracetamol 500mg	£0.86
Permethrin 5% w/w cream	£8.54
Podophyllotoxin 0.15% Cream	£17.83
Podophyllotoxin 0.5% Solution	£14.49
Raltegravir 400 mg bd for 3 days	£47.14
T- Safe 380A QL	£10.55
TT 380 Slimline	£12.46
Terbinafine Hydrochloride 1% Cream	£2.39
Trimethoprim 200mg	£1.16
Ulipristal Acetate 30mg	£14.05
Xylocaine 1% with adrenaline 1 :200,000	£1.77

Table S3. Intervention-related model estimates for females and males from sensitivity analyses using generalised additive models.

Outcome	Change at time of intervention (95% CI)	P-value for post-panther non-linearity of intervention site data
**MALES – 12th November 2018**		
Gonorrhoea culture swabs per consultation	-16.6% (-30.1%, -0.5%)	<0.001
Time to notification	+0.4 days (+0.27, +0.71)	0.03
**FEMALES – 29th May 2019**		
Gonorrhoea culture swabs per consultation	-11.1% (-29.8%, +12.6%)	<0.001
Time to notification	-0.16 days (-0.42%, +0.10)	<0.001

Figure S4. Modelled outcome estimates for males based on sensitivity analyses using generalised additive models. Both the overall time trend and the post-panther intervention site trend were estimated as splines with three degrees of freedom. All other covariates treated as in the main analysis.

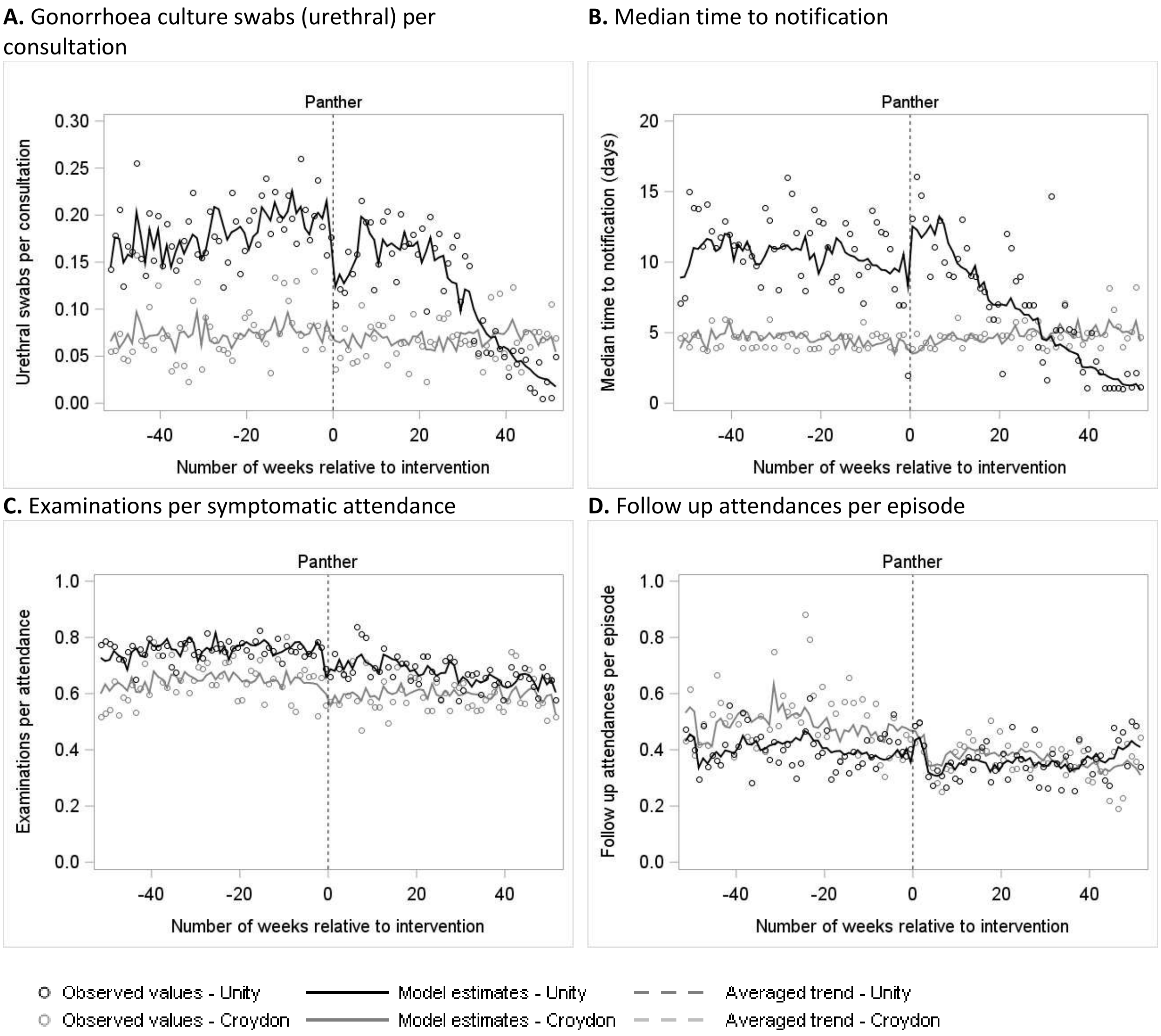

Figure S5. Modelled outcome estimates for females based on sensitivity analyses using generalised additive models. Both the overall time trend and the post-panther intervention site trend were estimated as splines with three degrees of freedom. All other covariates treated as in the main analysis.

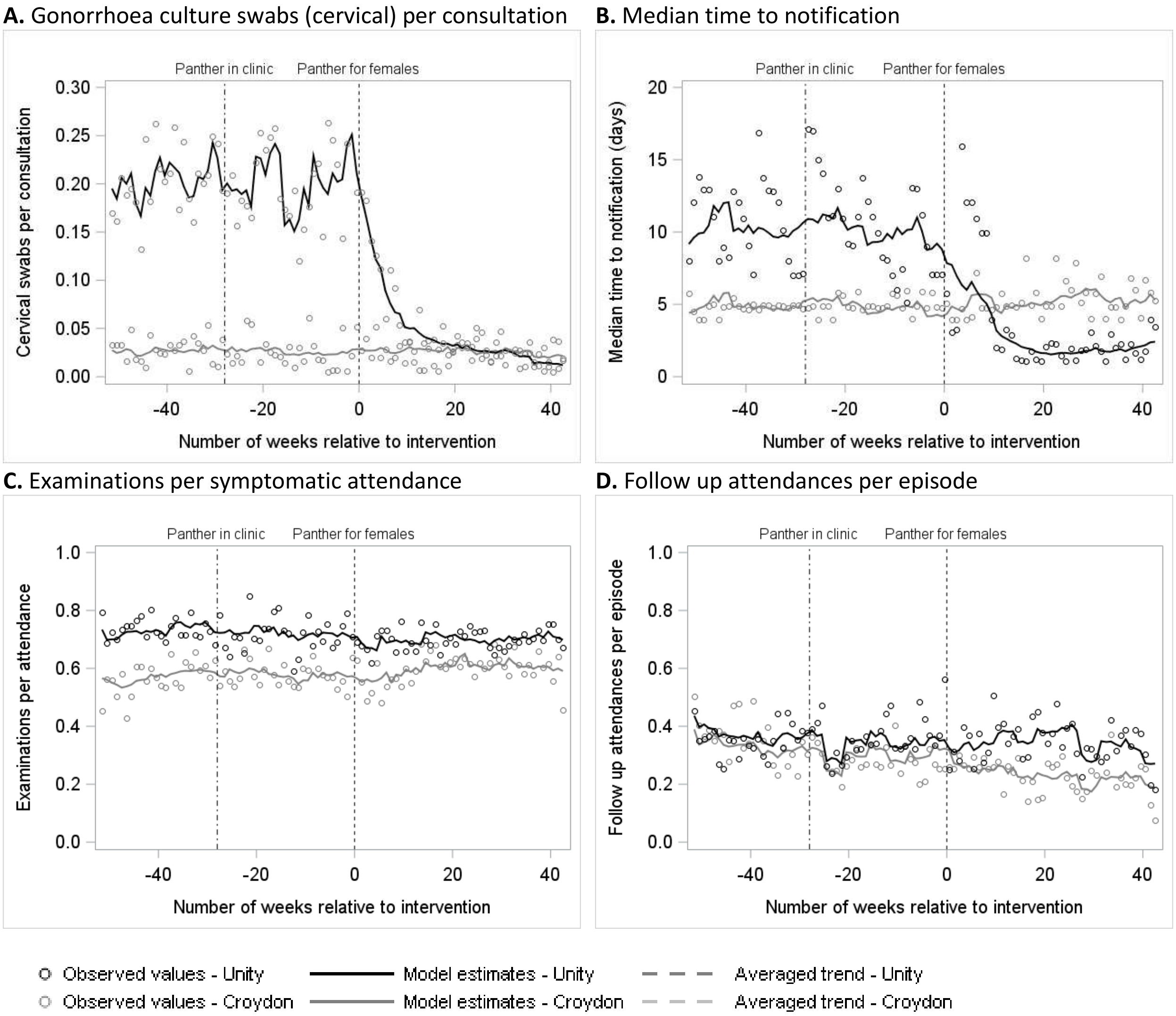

Figure S6. Modelled estimates of staff capacity for males and females combined. Time trends modelled with splines to allow for non-linearity. All other covariates treated as in the main analysis.

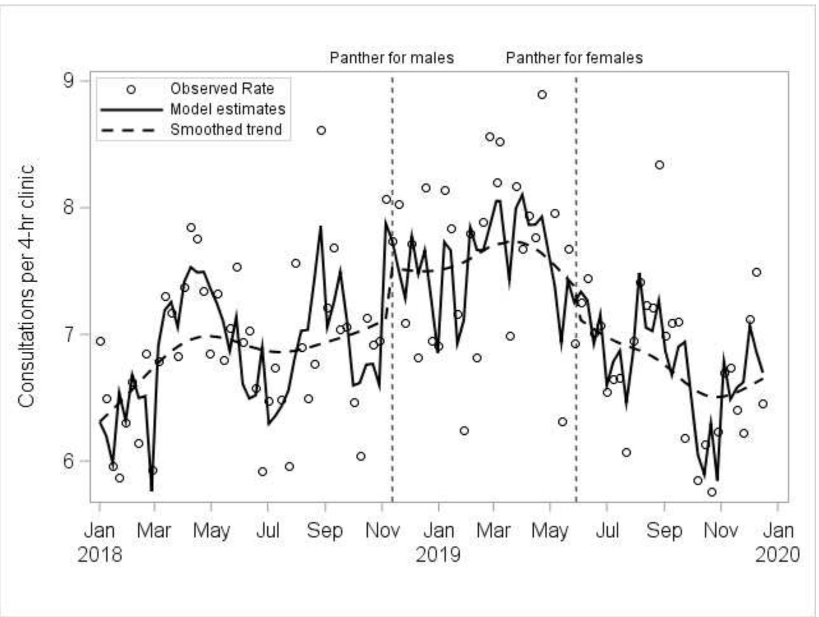

Definition of complex cases

Criteria for all patients:

a. Patients under 18 years of age
b. Have been/are currently exposed to child sexual exploitation, domestic violence, sexual assault
c. Has a current record of substance misuse
d. Has a current diagnosis of syphilis
e. Has current multiple diagnoses clinical diagnoses (GUMCAD coding B &/or C)
f. Has a history of/current diagnosis of genital herpes or had a swab taken for genital herpes
g. Has had post exposure prophylaxis after sexual exposure to HIV (PEPSE)
h. Needed an interpreter/use of translation service
i. Has current diagnosis of D2B on GUMCAD

Additional criteria for females:

i. Receive contraceptive care
ii. experienced pelvic pain, dyspareunia or post coital bleeding
iii. are pregnant
iv. experienced female genital mutilation.

Additional criteria for males:

v. are bisexual
vi. has sex with men
vii. Experienced testicular pain
viii. has a history/current record of chronic pelvic syndrome

Definition of an ethnic minority

This is self-reported at patient registration using the nationally-defined categories embedded within the patient record system.

Categories included in our definition are:

- African
- Caribbean
- Any other black background
- White and black Caribbean
- White and black African
- Indian
- Pakistani
- Bangladeshi
- Any other Asian background
- White and Asian
- Chinese
- Any other mixed background
- Any other ethnic group
