# Supplementary material for: Impact of rapid near-patient STI testing on service delivery outcomes in an integrated sexual health service in the United Kingdom: a controlled interrupted time series study
Source: BMJ Open. 2023 Jan 11;13(1):e064664. doi: 10.1136/bmjopen-2022-064664 (PMC9835959; doi:10.1136/bmjopen-2022-064664)
Supplement: Author's manuscript [file bmjopen-2022-064664.draft_revisions.pdf]

BMJ Open is committed to open peer review. As part of this commitment we make the peer review history of every article we publish publicly available.

When an article is published we post the peer reviewers' comments and the authors' responses online. We also post the versions of the paper that were used during peer review. These are the versions that the peer review comments apply to.

The versions of the paper that follow are the versions that were submitted during the peer review process. They are not the versions of record or the final published versions. They should not be cited or distributed as the published version of this manuscript.

BMJ Open is an open access journal and the full, final, typeset and author-corrected version of record of the manuscript is available on our site with no access controls, subscription charges or pay-per-view fees (http://bmjopen.bmj.com).

If you have any questions on BMJ Open's open peer review process please email info.bmjopen@bmj.com

BMJ Open

The impact of rapid near-patient STI testing on service delivery outcomes: a controlled interrupted time series study

Journal:	*BMJ Open*
Manuscript ID	bmjopen-2022-064664
Article Type:	Original research
Date Submitted by the Author:	17-May-2022
Complete List of Authors:	Walter, Scott; University of Bristol Medical School, NIHR Applied Research Collaboration West<br>Jackson, Joni; NIHR ARC West, ARC West; University of Bristol Medical School, Populations Health Sciences<br>Myring, Gareth; University of Bristol School of Social and Community Medicine<br>Redaniel, Maria Theresa; University of Bristol, NIHR CLAHRC West; University of Bristol, School of Social and Community Medicine<br>Margelyte, Ruta; University of Bristol, School of Social and Community Medicine; NIHR Collaboration for Leadership in Applied Health Research and Care West,<br>Gardiner, Rebecca; University Hospitals Bristol and Weston NHS Foundation Trust, Unity Sexual Health; University Hospitals Bristol and Weston NHS Foundation Trust, Bristol Haematology and Oncology Centre<br>Clarke, Michael; University Hospitals Bristol and Weston NHS Foundation Trust, Unity Sexual Health<br>Crofts, Megan; University Hospitals Bristol and Weston NHS Foundation Trust, Unity Sexual Health<br>McLeod, Hugh; University of Bristol<br>Hollingworth, William; University of Bristol, School of Social and Community Medicine<br>Phillips, David; Croydon University Hospital, Croydon Sexual Health<br>Muir, Peter; Public Health England, Specialist Virology Centre, Public Health Laboratory Bristol<br>Steer, Jonathan; Public Health England, Bristol Public Health Laboratory<br>Turner, Jonathan; Public Health England, Bristol Public Health Laboratory<br>Horner, Paddy; University of Bristol, School of Social and Community Medicine; University Hospitals Bristol NHS Fouindation Trust, Bristol Sexual Health Centre<br>De Vocht, Frank; University of Bristol,
Keywords:	SEXUAL MEDICINE, Epidemiology < INFECTIOUS DISEASES, HEALTH ECONOMICS

SCHOLARONE™
Manuscripts

I, the Submitting Author has the right to grant and does grant on behalf of all authors of the Work (as defined in the below author licence), an exclusive licence and/or a non-exclusive licence for contributions from authors who are: i) UK Crown employees; ii) where BMJ has agreed a CC-BY licence shall apply, and/or iii) in accordance with the terms applicable for US Federal Government officers or employees acting as part of their official duties; on a worldwide, perpetual, irrevocable, royalty-free basis to BMJ Publishing Group Ltd ("BMJ") its licensees and where the relevant Journal is co-owned by BMJ to the co-owners of the Journal, to publish the Work in this journal and any other BMJ products and to exploit all rights, as set out in our licence.

The Submitting Author accepts and understands that any supply made under these terms is made by BMJ to the Submitting Author unless you are acting as an employee on behalf of your employer or a postgraduate student of an affiliated institution which is paying any applicable article publishing charge ("APC") for Open Access articles. Where the Submitting Author wishes to make the Work available on an Open Access basis (and intends to pay the relevant APC), the terms of reuse of such Open Access shall be governed by a Creative Commons licence – details of these licences and which Creative Commons licence will apply to this Work are set out in our licence referred to above.

Other than as permitted in any relevant BMJ Author's Self Archiving Policies, I confirm this Work has not been accepted for publication elsewhere, is not being considered for publication elsewhere and does not duplicate material already published. I confirm all authors consent to publication of this Work and authorise the granting of this licence.

For peer review only

The impact of rapid near-patient STI testing on service delivery outcomes: a controlled interrupted time series study

Scott R Walter[1,2], Joni Jackson[1,2], Gareth Myring[1,2], Maria Theresa Redaniel[1,2], Ruta Margelyte[1,2], Rebecca Gardiner[3,4], Michael D Clarke[4], Megan Crofts[4], Hugh McLeod[1,2], William Hollingworth[1,2], David Phillips[5], Peter Muir[6,7], Jonathan Steer[6], Jonathan Turner[6], Paddy J Horner[2,3,7]*, Frank de Vocht[1,2]*

*joint last authors

Affiliations

1. National Institute for Health Research, Applied Research Collaboration West (NIHR ARC West) University Hospitals Bristol and Weston NHS Foundation Trust, Bristol, UK.
2. Population Health Sciences, Bristol Medical School, University of Bristol, Bristol, UK.
3. Unity Sexual Health, University Hospitals Bristol and Weston NHS Foundation Trust, Bristol, UK.
4. Bristol Haematology and Oncology Centre, University Hospitals Bristol and Weston NHS Foundation Trust, Bristol, UK.
5. Croydon Sexual Health
6. Southwest Regional Laboratory, UK Health Security Agency, North Bristol NHS Trust, Bristol, UK.
7. National Institute for Health Research, Health Protection Research Unit in Evaluation of Interventions, University of Bristol, Bristol, UK.

Keywords: sexual health; chlamydia; gonorrhoea; rapid STI testing

Corresponding author:
Scott R Walter
National Institute for Health Research
Applied Research Collaboration West (NIHR ARC West)
University Hospitals Bristol and Weston NHS Foundation Trust
9th Floor, Whitefriars, Lewins Mead
Bristol, BS1 2NT, UK
scott.walter@bristol.ac.uk

Word count: 3342

For peer review only - http://bmjopen.bmj.com/site/about/guidelines.xhtml

Abstract

Objectives: To evaluate the impact of a new clinic-based rapid STI testing, diagnosis and treatment service on healthcare delivery and resource needs in a sexual health service.

Design: Controlled interrupted time series study.

Setting: Two sexual health services in UK: Unity Sexual Health in Bristol, UK (main site) and Croydon Sexual Health in London (control site).

Participants: Electronic patient records for all attendances during the period one year before and one year after the intervention.

Intervention: Introduction of an in-clinic rapid testing system for gonorrhoea and chlamydia in combination with revised treatment pathways.

Outcome measures: Time-to-test notification, staff capacity, cost per episode of care and overall service costs. We also assessed rates of gonorrhoea culture swabs, follow-up attendances, and examinations.

Results: Time-to-notification and the rate of gonorrhoea swabs significantly decreased following implementation of the new system. There was no evidence of change in follow-up visits or examination rates for patients seen in clinic related to the new system. Staff capacity in clinics appeared to be maintained across the study period. Overall, the number of episodes per week was unchanged in the Unity SHS, and the mean cost per episode decreased by 7.5% (95%CI 5.7%, 9.3%).

Conclusions: The clear improvement in time-to-notification, while maintaining activity at a lower overall cost, suggests that the implementation of clinic-based testing in parallel to postal testing kits had the intended impact, which bolsters the case for more widespread rollout in SHS.

For peer review only - http://bmjopen.bmj.com/site/about/guidelines.xhtml

For peer review only

Strengths and limitations of this study

- We used controlled interrupted time series models with confounder adjustment to estimate the effect of the intervention distinct from any background changes and independent of other time varying factors.
- Model validity was bolstered by using a relatively long time series with good temporal resolution.
- Data from both the main and control sites was derived from the same electronic patient record system.
- There was a general consensus between main and sensitivity analyses.
- Our study was limited by being non-randomised, having only one control site, and the follow up period for females being truncated by the impact of the Covid-19 pandemic.

For peer review only

1. Introduction

Sexually transmitted infection (STI) diagnoses are increasing in England with more than a 10% increase in new infections between 2016 and 2019[1]. Over the same period, a 19.2% increase in total consultations at sexual health services (SHS) was reported in England[2]. Open-access SHS providing rapid treatment and partner notification can reduce the risk of STI complications and infection spread[3,4,5]. Public Health England (now UK Health Security Agency) recommends that local SHS need to be available to both the general population and groups with greater sexual health needs[3]. Nevertheless, the central government's public health grant, including SHS funding, has steadily decreased since 2015[6,7]. Despite diminishing resources, continued provision of SHS has been achieved through increased efficiencies at clinic-based services and introduction of online services[8,9].

Another approach to improving efficiency while ensuring quality, could be the introduction of near-patient testing (NPT) for chlamydia and gonorrhoea. Potential benefits include earlier diagnosis and treatment, reduced risk of sequelae and onward transmission, and reduction in unnecessary treatments, as well as reduced costs and clinician time due to reduction in gonorrhoea cultures, examinations and follow-up visits[10,11,12]. Although modelling studies suggest NPT can be cost-effective, this remains to be demonstrated in practice[10-14]. Research also suggests that reduced waiting times for STI test results may enhance patient acceptability[15,16] and increase testing uptake[17,18]. Importantly, patients have expressed preferences for earlier provision of results[19] due to the stress of waiting[20].

In November 2018, Unity Sexual Health (hereafter *Unity*), a UK specialist SHS, implemented a rapid nucleic acid amplification (NAAT) STI testing, diagnosis and treatment service for chlamydia and gonorrhoea, using the Hologic 'Panther' diagnostic platform in a clinic-based satellite laboratory [21]. It can deliver results in 3.5 hours by eliminating sample batching and transit times associated with microbiology laboratory testing.

For peer review only - http://bmjopen.bmj.com/site/about/guidelines.xhtml

We used a quantitative approach to evaluate the impact of the new rapid testing process on service delivery and resource needs of the Unity SHS.

2. Methods

2.1 Setting and design

This study is a quasi-experimental, controlled interrupted time series (CITS) design that used routinely collected electronic patient record (EPR) data. The intervention time points were defined differently for males and females: rapid STI testing was introduced on 12 November 2018 for males and 29 May 2019 for females.

2.2 Rapid STI service model

Eligibility criteria and treatment pathways differed for males and females. A graphical overview of each pathway is provided in the supplement (Figures S1 and S2) with pre-intervention pathway included for reference. Additional changes were made to the SHS related to staff capacity. Rapid STI asymptomatic consultations were reduced to 15 minutes, while the number of allocated patients per staff member for the walk-in clinic remained the same.

2.2.1 Males

Male patients were eligible for the rapid STI pathway if they were asymptomatic or had urethritis symptoms. If asymptomatic, a brief history was taken prior to patient self-sampling for chlamydia and gonorrhoea and taking blood tests for HIV and syphilis. Men who have sex with men (MSM) were referred to a health adviser. Symptomatic men were asked to return four hours later when NAAT results were available. If positive, they received infection specific treatment; if negative a urethral smear was undertaken to diagnose non-gonococcal urethritis. Contacts of patients with gonorrhoea or chlamydia outside a two-week window were treated if NAAT-positive. Swabs for

For peer review only - http://bmjopen.bmj.com/site/about/guidelines.xhtml

gonococcal culture and sensitivities were only taken after a NAAT-positive result for gonorrhoea or if gonococcal treatment was administered prior to the NAAT result.

2.2.2 Females

Female asymptomatic patients without contraception needs were eligible for the rapid drop-off service. Women with abnormal vaginal discharge, not requiring bimanual or speculum examination to exclude pathology, self-swabbed and were treated on the results of microscopy and clinical findings at the time of visit and informed that chlamydia and gonorrhoea NAAT test results would be available within 48 hours. Trichomonas vaginalis (TV) culture was replaced with a more sensitive TV NAAT[22], also available within 48 hours. For contraceptive needs, a clinical consultation was necessary to determine the need for examination. A gonococcal culture swab was only taken after a NAAT-positive result for gonorrhoea or if gonococcal treatment was administered prior to NAAT result.

2.3 Control site

Croydon Sexual Health, a similar SHS in South London, was used as the control site to account for background changes unrelated to the intervention. This site has similar patient throughput (about 32,000 annual attendances compared to about 40,000 for Unity) and uses the same EPR system.

2.4 Data

Fully anonymised individual patient data extracted from the Unity and Croydon EPR systems[23] comprised demographic information, sexual behaviour, mode of presentation and attendances to the clinic, diagnostic testing and treatment. Analyses were based on a census of attendance level records.

Time-to-notification was defined from the text message notification system[23]. This included text message type for identifying test results messages, time stamps and anonymised

For peer review only - http://bmjopen.bmj.com/site/about/guidelines.xhtml

patient identifiers. Numbers of NAAT postal testing kits were extracted from Unity's records, while Croydon did not implemented these until after the study period.

Prior to analysis, data were checked for duplicates, implausible values and missingness. Individual variables were combined to generate indicator variables for complex cases, MSM, examinations, ethnic minority status. All time-related variables were derived from the date and time of each attendance.

For analysis, data were aggregated at weekly level over a two-year period centred at the intervention. For females, data were excluded from the first UK Covid-19-related lockdown (23 March 2020) due to changes in outcomes that could not be adequately accounted for in models. The study period for males was from 13 November 2017 to 10 November 2019, and for females 28 May 2018 to 22 March 2020.

2.5 Statistical analysis

There main study outcomes are detailed in Table 1. CITS models within a generalised linear modelling framework were applied to each outcome separately for males and females: ten models in total. $Time$ was modelled as linear using consecutively numbered weeks, with $time = 0$ at the intervention point. A binary variable ($period$) representing pre- and post-intervention periods was defined by the respective male and female intervention dates.

Gonorrhoea culture swabs per consultation, follow-up attendances per care episode, examinations per symptomatic attendance and staff capacity were modelled as rates assuming a negative binomial distribution. These models generate rate ratios, presented as percentage changes. For time-to-notification, a normal distribution was assumed and results presented as differences in median time (days). This represents absolute measure of time including weekends as opposed to working days only.

For peer review only - http://bmjopen.bmj.com/site/about/guidelines.xhtml

Table 1. Definitions of main study outcomes.

Outcome measure	Definition
1. Rate of gonorrhoea culture swabs per consultation	Numerator: the number of GC swabs, urethral for male and cervical for female<br>Denominator: the number of consultations where these were defined as attendances for new, rebooked or walk-in patients
2. Time to notification	Median time from sample collection until the patient was notified of the test result via text message
3. Rate of examinations per symptomatic attendance	Numerator: the number of examinations of any type. This was based on a combination of variables used to record information about examinations (supplementary Table S1)<br>Denominator: all attendances where the patient was recorded as being symptomatic
4. Rate of follow up attendances per episode of care	Numerator: the number of follow up attendances occurring within 30 days of an initial consultation<br>Denominator: the number of episodes involving at least 1 consultation
5. Staff capacity – rate of patients seen per four-hour clinic	Numerator: number of patient consultations (any new, rebooked, walk-in or follow up attendance)<br>Denominator: number staff available for four-hour clinics

The main variables in the models were $time$, $period$ and $site$ (Unity vs. Croydon) along with all two-way and three-way interactions, as per a CITS approach for estimating both a step change and slope change[24,25]. Two key terms in the models represent intervention-related changes over and above any control site changes. The interaction $period \times site$ captures a differential step change for the intervention site compared to control site. While the three-way interaction term $time \times period \times site$ captures different degrees of pre-post trend change for the intervention site compared to control site (supplement Figure S3).

Additional covariates were included in the models: proportions of complex patients, symptomatic patients and patients from an ethnic minority, plus mean patient age and calendar month. Since models of examination rate only analysed symptomatic patients, the proportion of symptomatic patients was excluded as a covariate. The proportion of MSM was only included in models for males. Complex cases were defined differently for males and females (definition S1).

Data for staff capacity was only available for Unity and was modelled as an uncontrolled interrupted time series spanning the duration of available denominator data: 1 January 2018 to 22

For peer review only - http://bmjopen.bmj.com/site/about/guidelines.xhtml

December 2019. The denominator could not be separated by gender, so this outcome was analysed for females and males combined, allowing two change points as per the respective intervention dates.

Where outcomes showed marked change over time, sensitivity analyses were conducted by fitting generalised additive models to account for potential non-linearity of trends. All analyses were conducted with the SAS System for Windows, version 9.4 (SAS Institute Inc.). Models were fitted using the GENMOD and GAM procedures.

2.6 Economic analysis

Postal testing kit data were combined with EPR data to estimate the total number of episodes per week (including those with negative postal tests and no clinic attendance). For estimating the difference in the mean number of episodes per week i) negative postal test episodes were assigned to weeks pro rata with asymptomatic episodes that included clinic attendance, and ii) the combined post-intervention analysis used data for the first 43 weeks only. Episode costs were estimated using unit costs of diagnostic tests provided by Unity SHS, and postal kit tests and staff time from the literature[12] inflated to 2021 values using a UK government GDP deflator[26]. Treatment costs were from the British National Formulary[27] (supplement Table S2). The cost of unreturned postal kits was allocated to episodes including a postal test result. Confidence intervals for differences in the number of episodes and cost per episode were calculated using the Normal approximation method.

Patient and public involvement

Three members of the public who had used Unity services as patients were involved in reviewing the proposed outcome measures and informed the study design.

For peer review only - http://bmjopen.bmj.com/site/about/guidelines.xhtml

3. Results

In the EHR Unity data, 48,776 attendances for females and 34,413 for males were recorded during the study period, representing 32,482 and 22,073 episodes of care involving a clinic attendance, and 29,573 and 19,083 patients, respectively (Table 2). Patients were symptomatic in just over 20% of female attendances, and over 40% of male attendances. About 90% of female and 55% of male attendances were complex. Just over 30% of male attendances were by MSM.

3.1 Males

There were significant changes in the rate of gonorrhoea culture swabs for males associated with the intervention. A small increase at the time of the intervention for Unity (+6.5%) compared to a large decrease for Croydon (-43.7%), resulted in a significant adjusted step-increase for Unity (+89.1%, 95% confidence interval [CI] +37.1%, +160.6%, p<0.001) (Table 3 and Figure 1A). However, this was not observed in the sensitivity analysis allowing for non-linear trends (supplement Table S3 and Figure S4A). This was followed by a significant adjusted downward change in post-intervention trend of -3.2% per week (95% CI -4.3%, -2.1%, p<0.001). The long-term result of these two effects was an overall decrease from 35-50 swabs per week, pre-intervention, to below 10 at the end of the study period, translating to 849 swabs avoided over the post-intervention period.

Time-to-notification increased by an estimated 3.6 days (95% CI 1.7, 5.5 days, p<0.001) at the time of the intervention, relative to controls, and a similar increase was observed in the sensitivity analysis. However, this was followed by an overall long-term decrease of -0.2 days per week (95% CI -0.3, -0.2 days, p<0.001) through the post-intervention period. That is, the pre-intervention weekly median of around eight to nine days dropped to around 2 days after the Panther system had been in place for a year (Figure 1B).

We found no evidence of a meaningful change in rates of examinations or follow-up attendances associated with the intervention.

For peer review only - http://bmjopen.bmj.com/site/about/guidelines.xhtml

For peer review only

Table 2. Summary of population characteristics and outcomes by site, gender and time period based on EPR data.

	**Unity**		**Croydon**	
	**Pre**	**Post**	**Pre**	**Post**
**MALES**				
Total attendances, n	17626	16787	11920	12085
Total episodes of care, n	11445	10628	7946	8021
Total patients, n	9932	9151	6271	6335
Symptomatic attendances, n (%)	7307 (41.5%)	7084 (42.2%)	4735 (39.7%)	4556 (37.7%)
Complex attendances, n (%)	9869 (56.0%)	9259 (55.2)%	4458 (37.4%)	4940 (40.9%)
Ethnic minority attendances, n (%)	2834 (16.1%)	3025 (18.0%)	7244 (60.8%)	7311 (60.5%)
MSM attendances, n(%)	5300 (30.1%)	5418 (32.3%)	2529 (21.2%)	2849 (23.6%)
Mean age, years	30.2	30.8	34.9	35.1
Urethral GC swabs per consultation	0.18	0.11	0.08	0.07
Median time to notification	10.90	6.73	4.51	4.95
Examinations per symptomatic attendance	0.76	0.67	0.64	0.60
Follow up attendances per episode	0.40	0.36	0.50	0.37
**FEMALES**				
Total attendances	28487	20289	20931	16910
Total episodes of care	18616	13866	13971	11660
Total patients	16779	12794	11799	9902
Symptomatic attendances	6312 (22.2%)	4929 (24.3%)	6860 (32.8%)	5561 (32.9%)
Complex attendances	26022 (91.3%)	18173 (89.6%)	12328 (58.9%)	11221 (66.4%)
Ethnic minority attendances	3979 (14.0%)	3067 (15.1%)	12647 (60.4%)	10107 (59.8%)
Mean age	25.1	25.8	29.8	30.4
Cervical GC swabs per consultation	0.20	0.04	0.03	0.03
Median time to notification (median, IQR)	10.58	3.52	4.90	5.32
Examinations per symptomatic attendance	0.73	0.70	0.58	0.60
Follow up attendances per episode	0.36	0.34	0.31	0.23

For peer review only

Table 3. Intervention-related model estimates for females and males.

Outcome	Change at time of intervention			Trend change following intervention		
	Intervention series	*Control series*	*Intervention vs. control, % change (95% CI)*	*Intervention series*	*Control series*	*Intervention vs. control, % change per week (95% CI)*
**MALES – 12th November 2018**						
**1. Gonorrhoea culture swabs per consultation**	+6.5%	-43.7%	**+89.1% (+37.1%, +160.9%)**	-3.6%	-0.3%	**-3.2% (-4.3%, -2.1%)**
**2. Time to notification**	+2.2 days	+5.8 days	**+3.6 (+1.7, +5.5) days**	-0.19 days	+0.03 days	**-0.2 (-0.3, -0.2) days**
**3. Examinations per symptomatic attendance**	+3.6%	-1.6%	+5.4% (-7.5%, +20.0%)	-0.21%	-0.16%	-0.04% (-0.5%, +0.4%)
**4. Follow up attendances per episode**	-9.0%	-11.9%	+3.3% (-14.6%, +24.9%)	+0.23%	-0.001%	+0.30% (+0.31%, +0.96%)
**FEMALES – 29th May 2019**						
**1. Gonorrhoea culture swabs per consultation**	-38.7%	+3.6%	**-40.8% (-61.6%, -8.8%)**	-6.1%	-0.1%	**-6.1% (-7.8%, -4.5%)**
**2. Time to notification**	-2.5 days	-0.4 days	-2.1 (-4.5, 0.3) days	-0.11 days	-0.0001 days	**-0.1 (-0.2, -0.0) days**
**3. Examinations per symptomatic attendance**	-1.3%	-2.2%	+1.0% (-11.4%, +15.1%)	+0.09%	+0.03%	+0.1% (-0.4%, +0.5%)
**4. Follow up attendances per episode**	-8.2%	+2.7%	-10.6% (-27.6%, +10.3%)	-0.42%	+0.22%	-0.64% (-1.41%, +0.14%)

Note: Results for outcome 5 (staff capacity) reported separately in the text.

For peer review only

For peer review only - http://bmjopen.bmj.com/site/about/guidelines.xhtml

3.2 Females

For females, there was significant decrease in the rate of gonorrhoea culture (GC) swabs: -40.8% (95% CI -61.6%, -8.8%, p=0.02) at the time of intervention, adjusted for control changes (Table 3, Figure 2A). This was followed by a significant decrease in trend through the post-intervention period, with an adjusted change of -6.1% per week (95% CI -7.8%, -4.5%, p<0.001). These changes represent a decrease from an estimated 0.22 swabs per consultation (over 30 swabs per week) immediately before the intervention to 0.14 immediately after (20 to 25 per week) and down to 0.01 at the end of the study period (less than five per week). Over the 43-week post-intervention period, an estimated 1542 swabs were avoided.

For time-to-notification, there was some evidence of a decrease of 2.1 days (95% CI -4.5, 0.3 days, p=0.08) at the time of the intervention, adjusted for the control group, although this estimate does not rule out chance. There was stronger evidence of a downward change in trend, estimated at -0.1 days per week (95% CI -0.20, -0.0 days, p=0.01) over the post-intervention period. These results were confirmed by the sensitivity analyses (Figure S5). To illustrate, the estimated median time-to-notification was eight to nine days just before the intervention, but a year later had dropped to around one day.

[Figures 1 and 2 about here]

3.3 Staff capacity

The main analysis of staff capacity showed a significant trend change at the time of the male intervention (-1.1% per week, 95%CI -1.7%, -0.5%, p<0.001) and a significant step change at the time of the female intervention (+14.3%, 95% CI +3.4%, +26.3%, p=0.009) (Figure 3). However, the sensitivity analysis showed step changes in the opposite direction to the main analysis (supplement Figure S6), suggesting inconclusive evidence of change.

[Figure 3 about here]

3.4 Episodes and costs

For peer review only - http://bmjopen.bmj.com/site/about/guidelines.xhtml

Overall, the Unity SHS experienced a substantial increase in the weekly number of asymptomatic negative episodes managed via postal test kits, particularly for males, while both asymptomatic negative episodes seen in the clinic and symptomatic episodes decreased (Table 4). The mean cost per symptomatic episode increased by 9.2% to £69.04, while this was outweighed by a decrease of 13.5% to £26.23 for costs per asymptomatic episode, resulting in a combined decrease of 7.5%. The total cost per week decreased by 4.7%, largely due to the reduction in both the number and cost of episodes for asymptomatic females who attended the clinic.

For peer review only

For peer review only - http://bmjopen.bmj.com/site/about/guidelines.xhtml

Table 4. Unity clinic pre- and post-intervention estimates of mean number of episodes per week, mean cost per episode and mean cost per week.

	Male					Female					Total				
	pre*	post*	% change	95% CI		pre*	post**	% change	95% CI		pre*	post**	% change	95% CI	
Mean number per week															
Asymptomatic	190.2	223.1	17.3	9.5	25.1	356.2	350.7	-1.5	-7.9	4.9	546.3	573.4	5.0	0.0	9.9
Postal negatives	70.5	111.5	58.2	48.7	67.7	96.3	124.9	29.7	22.3	37.0	166.8	236.2	41.6	35.7	47.4
Other^	119.6	111.5	-6.8	-13.7	0.2	259.9	225.9	-13.1	-19.2	-7.0	379.5	337.2	-11.1	-15.8	-6.5
Symptomatic	92.7	85.0	-8.3	-13.9	-2.7	84.4	77.8	-7.8	-14.6	-1.1	176.7	163.4	-7.5	-11.8	-3.2
Total	282.8	308.0	8.9	2.6	15.2	440.2	429.0	-2.5	-8.7	3.6	723.0	736.8	1.9	-2.5	6.3
Cost per episode (£)															
Asymptomatic	36.47	30.92	-15.2	-19.1	-11.3	27.04	24.23	-10.4	-13.3	-7.5	30.31	26.23	-13.5	-15.9	-11.0
Symptomatic	63.09	69.56	10.3	6.7	13.8	63.36	67.65	6.8	4.3	9.2	63.22	69.04	9.2	6.9	11.5
Total	45.19	41.58	-8.0	-10.8	-5.2	33.98	32.14	-5.4	-7.7	-3.1	38.36	35.47	-7.5	-9.3	-5.7
Cost per week (£)															
Resource															
Postal kit	382	592	55.0	45.9	64.1	629	848	34.8	27.4	42.2	1010	1437	42.3	36.5	48.1
In clinic diagnostic test	1962	1886	-3.9	-9.8	2.1	1452	1213	-16.5	-22.9	-10.1	3413	3155	-7.6	-11.9	-3.3
Consultation staff time	7497	7349	-2.0	-7.3	3.4	9396	8583	-8.7	-15.0	-2.3	16893	15959	-5.5	-9.5	-1.5
Treatment	3024	2896	-4.2	-13.1	4.6	3534	3085	-12.7	-20.3	-5.1	6558	6014	-8.3	-14.4	-2.2
Symptom status															
Asymptomatic	6949	6883	-1.0	-8.5	6.6	9673	8448	-12.7	-18.9	-6.4	16622	15392	-7.4	-12.3	-2.5
Symptomatic	5915	5840	-1.3	-7.5	5.0	5338	5280	-1.1	-8.7	6.6	11253	11174	-0.7	-5.5	4.0
Total	12865	12723	-1.1	-6.7	4.5	15010	13728	-8.5	-14.4	-2.6	27875	26565	-4.7	-8.6	-0.8

* based on 52 week period.

** based on 43 week period

^ includes positive postal test kits

For peer review only

For peer review only - http://bmjopen.bmj.com/site/about/guidelines.xhtml

4. Discussion

We have quantitatively evaluated the impact of a first-of-its-kind integrated rapid STI testing on service delivery. Previous NPT assessments have taken a mathematical modelling approach[11-13]. The only other direct assessment of a chlamydia and gonorrhoea NPT in practice related to a rapid testing service model for asymptomatic patients[28]. This is the first study to quantify the effect of rapid chlamydia and gonorrhoea NPT on gonorrhoea culture swabs, time-to-notification, examinations, follow-up visits, staff capacity, and costs.

The substantial long term post-intervention decrease in the rate at which gonorrhoea swabs were sent for culture, for both males and females, was expected to some extent since patients with negative rapid tests in the new pathway avoided the need for cultures. Adams et al. [11] identified reduced gonorrhoea cultures as a key part of NPT-related cost reduction, although there has been no direct or simulated assessment of expected change in the number of cultures.

The trajectory of the decline in gonorrhoea swab rates following the intervention differed between males and females. The sensitivity analysis capturing non-linear trends suggested substantial decreases for males began more than six months after the intervention, with the lowest rates at one year post-intervention (Figure S4A). In contrast, rates for females appeared to respond to the intervention almost immediately and stabilise at a much lower level within about six months (Figure S5A). The differing implementation timeframes may reflect several barriers to implementation with the initial rollout for males, including providing training to a large group staff with varying timetables exacerbated by understaffing and budget cuts; variable application of eligibility criteria for the new service; and iterative revision of the new system and pathway[29]. There may also have been some just-in-case culture testing in the early stages until staff confidence in the system was established. With these issues largely resolved when the system was implemented for females, the transition appeared both smoother and faster, and this concurs with staff experience.

For peer review only - http://bmjopen.bmj.com/site/about/guidelines.xhtml

We estimated that median time-to-notification decreased from more than a week down to one or two days over the post-intervention period. However, given that it was not possible to separate out all rapid test results (e.g. notifications labelled “all negative”) and that we estimated real time rather than working days, the median time was likely lower, particularly for positive results. This is broadly consistent with findings from Whitlock et al. [28] who reported an average time-to-notification of 0.27 days for a new rapid NAAT testing service compared to 8.95 days for an off-site testing service for symptomatic patients.

The temporary increase in median time-to-notification for males after the intervention may result from the implementation challenges outlined above[29] in addition to a clinician-reported backlog in the early stages of transitioning to the new system. Once again, for males the transition appeared to take place over the full post-intervention period, while the equivalent period for females appeared faster with the lowest post-intervention sensitivity estimates occurring 21 weeks after the new system was implemented (supplement Figures S4B and S5B).

We observed no clear evidence of intervention-related changes in rates of examinations, follow up visits or staff capacity. All three were necessarily constructed from combinations of variables as there was no dedicated data field for each in the data. Although we did not detect a positive change, it is important to note that there was no evidence of a deleterious impact of the rapid testing service on any of these outcomes.

Staff capacity showed some evidence of intervention-related change, although the rate of patients seen per four-hour clinic was at similar levels at the end of the study period as at the start. For asymptomatic patients, the provision of postal testing kits and the introduction of shorter appointments more than likely increased staff capacity for this subgroup. It also reduced the queueing time for walk-in clinics. Conversely, case-mix in the walk-in clinics became more demanding, with patients more likely to be symptomatic and/or complex[29], which may explain the lack of observed improvement in staff capacity during clinics. The lack of evidence for a capacity decrease through the implementation period despite a more demanding patient group and the

For peer review only - http://bmjopen.bmj.com/site/about/guidelines.xhtml

growing numbers of asymptomatic patients being tested both suggest increased capacity of the SHS overall.

The change in management of asymptomatic clinical attendances, supported by the existing postal testing kit system, was a key component of the overall cost reduction following the introduction of the Panther technology, with decreases in both mean cost per asymptomatic episode (13.5%) and weekly asymptomatic costs (7.4%). Although the cost of symptomatic episodes increased, consistent with the reported increase in complexity of symptomatic patients in clinic, this was counteracted by a reduction in the number of weekly symptomatic attendances.

4.1 Strengths and limitations

We conducted a prospective real-time evaluation of a large integrated rapid STI service. We used a CITS framework with both a control site and confounder adjustment to estimate the effect of the intervention distinct from any background changes and independent of other time varying factors. This was bolstered by using a relatively long time series with good temporal resolution. The robustness of our analysis was supported by both sites using the same EPR system and the general consensus between main and sensitivity analyses.

In light of the target trial framework for natural experiments[30], our study was limited by being non-randomised, having only one control site, relying on the construction of certain outcomes from multiple variables, and the impact of the Covid-19 pandemic on the follow up period for females. The unit costs were based on data provided by Unity SHS and estimates from literature, and commissioners will need to assess their applicability to their locality.

4.2 Implications and conclusions

Several studies have suggested that NPT benefits include earlier diagnosis and treatment, reduced risk of sequelae and onward transmission, reduction in unnecessary treatments, earlier partner notification and reduced anxiety [10,28].

For peer review only - http://bmjopen.bmj.com/site/about/guidelines.xhtml

This quantitative assessment of the first UK implementation of rapid chlamydia and gonorrhoea testing within an integrated service revealed clear benefits, namely: reduced gonorrhoea culture swabs and shortened time-to-notification. These improvements, while maintaining activity at a lower overall cost, suggests that the introduction of clinic-based rapid testing had the intended impact, and this is in line with previous NPT modelling studies [10,11]. The qualitative evaluation of this rapid STI service also reported that patients valued faster results and avoiding unnecessary treatment, and that the better targeting of infection-specific treatment improved antimicrobial stewardship[29].

These results provide real-life evidence to support the benefits of a rapid testing service anticipated by modelling studies and strengthen the case for more widespread rollout in SHS.

For peer review only

For peer review only - http://bmjopen.bmj.com/site/about/guidelines.xhtml

Acknowledgements: The authors would like to thank Ed Hulse at Mill Systems for his indispensable assistance with the data extracts.

Funding statement: This research was funded by the National Institute for Health Research (NIHR) Applied Research Collaboration West (ARC West) at University Hospitals Bristol and Weston NHS Foundation Trust (core NIHR infrastructure funded: NIHR200181). The views expressed are those of the authors and not necessarily those of the NIHR or the Department of Health and Social Care. FdV is partly funded by the NIHR School for Public Health Research.

Competing interests: There are no competing interests to declare for any of the authors.

Data sharing statement: Anonymised individual-level data for this study comes from the electronic patient record system of the Unity Sexual Health and Croydon Sexual Health services (data controllers). Our data sharing agreement with the data controllers prohibits sharing data extracts outside of the University of Bristol research team. The data is available upon request from the data controllers.

Licence statement: I, the Submitting Author has the right to grant and does grant on behalf of all authors of the Work (as defined in the below author licence), an exclusive licence and/or a non-exclusive licence for contributions from authors who are: i) UK Crown employees; ii) where BMJ has agreed a CC-BY licence shall apply, and/or iii) in accordance with the terms applicable for US Federal Government officers or employees acting as part of their official duties; on a worldwide, perpetual, irrevocable, royalty-free basis to BMJ Publishing Group Ltd ("BMJ") its licensees and where the relevant Journal is co-owned by BMJ to the co-owners of the Journal, to publish the Work in BMJ Open and any other BMJ products and to exploit all rights, as set out in our licence.

The Submitting Author accepts and understands that any supply made under these terms is made by BMJ to the Submitting Author unless you are acting as an employee on behalf of your employer or a postgraduate student of an affiliated institution which is paying any applicable article publishing charge ("APC") for Open Access articles. Where the Submitting Author wishes to make the Work available on an Open Access basis (and intends to pay the relevant APC), the terms of reuse of such Open Access shall be governed by a Creative Commons licence – details of these licences and which Creative Commons licence will apply to this Work are set out in our licence referred to above.

Ethics approval: This study was approved by the Health Research Authority (South West) Research Ethics Committee, reference 18/SW/0090.

Contributorship statement: PH, MTR, FdV and HM conceptualized the evaluation; MTR and FdV are quantitative evaluation leads; WH and HM are health economic evaluation leads; SW, JJ, RM and MTR acquired the analysis datasets; SRW conducted the effectiveness analysis with supoprt from JJ, RM, MTR, PH and FdV; GM conducted the cost-effectiveness analysis with support from HM and WH; RG, MDC, MC, DP, PM, JS and JT advised on the study methodology, analysis and interpretation of results; SRW wrote the initial draft of the manuscript; all authors reviewed and edited the manuscript for content and approved the submission.

For peer review only

References

1. Public Health England, National STI surveillance data tables 2020 - Table 4. Available at: https://www.gov.uk/government/statistics/sexually-transmitted-infections-stis-annual-data-tables

2. Public Health England, National STI surveillance data tables 2020 - Table 3. Available at: https://www.gov.uk/government/statistics/sexually-transmitted-infections-stis-annual-data-tables

3. Mitchell H, Allen H, Sonubi T, Kuyumdzhieva G, Harb A, Shah A, Glancy M, Checchi M, Milbourn H, Folkard K, Mohammed H and contributors. Sexually transmitted infections and screening for chlamydia in England, 2019. September **2020**. London: Public Health England. Available at: https://assets.publishing.service.gov.uk/government/uploads/system/uploads/attachment_data/file/914249/STI_NCSP_report_2019.pdf

4. Price MJ, Ades AE, Soldan K, Welton NJ, Macleod J, Simms I, DeAngelis D, Turner KM, Horner PJ. The natural history of Chlamydia trachomatis infection in women: a multi-parameter evidence synthesis. Health Technol Assess, **2016**; 20: 1-250.

5. Ratna N, Sonubi T, Glancy M, Sun S, Harb A, Checchi M, Milbourn H, Dunn J, Sinka K, Folkard K, Mohammed H and contributors. Sexually transmitted infections and screening for chlamydia in England, 2020. September **2021**. London: Public Health England. Available at: https://assets.publishing.service.gov.uk/government/uploads/system/uploads/attachment_data/file/1015176/STI_NCSP_report_2020.pdf

6. Finch D, Bibby J, Elwell-Sutton T. Briefing: Taking our Health for Granted. October **2018**. London: The Health Foundation. Available online: https://www.health.org.uk/publications/taking-our-health-for-granted

7. White C. Sexual health services on the brink. Brit Med J, **2017**; 359: j5395.

8. Turner KME, Zienkiewicz AK, Syred J, Looker KJ, de Sa J, Brady M, Free C, Holdsworth G, Baraitser P. Web-Based Activity Within a Sexual Health Economy: Observational Study. J Med Internet Res, **2018**; 20:e74.

9. Turner KME, Looker KJ, Syred J, Zienkiewicz A, Baraitser P. Online testing for sexually transmitted infections: A whole systems approach to predicting value. PLoS ONE, **2019**; 14: e0212420. doi: 10.1371/journal.pone.0212420

10. Turner KME, Round J, Horner PJ, Macleod J, Goldenberg S, Deol A, Adams EJ. An early evaluation of clinical and economic costs and benefits of implementing point of care NAAT tests for Chlamydia trachomatis and Neisseria gonorrhoea in genitourinary medicine clinics in England. Sex Transm Infect, **2014**; 90: 104–111. doi: 10.1136/sextrans-2013-051147

11. Adams EJ, Ehrlich A, Turner KME, Shah K, Macleod J, Goldenberg S, Meray RK, Pearce V, Horner PJ. Mapping patient pathways and estimating resource use for point of care versus standard testing and treatment of chlamydia and gonorrhoea in genitourinary medicine clinics in the UK. BMJ Open, **2014**; 4: e005322. doi: 10.1136/bmjopen-2014-005322

12. Mohiuddin S, Gardiner R, Crofts M, Muir P, Steer J, Turner J, Wheeler H, Hollingworth P, Horner PJ. Modelling patient flows and resource use within a sexual health clinic through discrete event

For peer review only - http://bmjopen.bmj.com/site/about/guidelines.xhtml

simulation to inform service redesign. BMJ Open, **2020**;10: e037084. doi: 10.1136/bmjopen-2020-037084

13. Huntington SE, Burns RM, Harding-Esch E, Harvey MJ, Hill-Tout R, Fuller SS, Adams EJ, Sadiq ST. Modelling based evaluation of the costs, benefits and cost-effectiveness of multipathogen point-of-care tests for sexually transmitted infections in symptomatic genitourinary medicine clinic attendees. BMJ Open, **2018**; 8: e020394. doi: 10.1136/bmjopen-2017-020394

14. Miners A. Is nucleic acid amplification point-of-care testing for chlamydia and gonorrhoea cost-effective? Sex Transm Infect, **2014**; 90: 82.

15. Natoli L, Guy RJ, Shephard M, Causer L, Badman SG, Hengel B, Tangey A, Ward J, Coburn T, Anderson D, Kaldor J, Maher L. "I Do Feel Like a Scientist at Times": A Qualitative Study of the Acceptability of Molecular Point-Of-Care Testing for Chlamydia and Gonorrhoea to Primary Care Professionals in a Remote High STI Burden Setting. PloS ONE, **2016**; 10: e0145993. doi: 10.1371/journal.pone.0145993

16. Rompalo AM, Yu-Hsiang H, Hogan T, Barnes M, Jett-Goheen M, Huppert JS, Gaydon CA. Point-of-care tests for sexually transmissible infections: what do 'end users' want? Sex Health, **2013**; 10: 541-545. doi: 10.1071/SH13047

17. Horwood J, Ingle SM, Burton D, Woodman-Bailey A, Horner PJ, Jeal N. Sexual health risks, service use, and views of rapid point-of-care testing among men who have sex with men attending saunas: a cross-sectional survey. Int J STD AIDS, **2016**; 27: 273-280. doi: 10.1177/0956462415580504

18. Lorenc T, Marrero-Guillamón I, Aggleton P, Cooper C, Llewellyn A, Lehmann A, Lindsay C. Promoting the uptake of HIV testing among men who have sex with men: systematic review of effectiveness and cost-effectiveness. Sex Transm Infect, **2011**; 87: 272-278. doi: 10.1136/sti.2010.048280

19. Llewellyn CD, Sakal C, Lagarde M, Pollard A, Miners AH. Testing for sexually transmitted infections among students: a discrete choice experiment of service preferences. BMJ Open, **2013**; 3. doi: 10.1136/bmjopen-2013-003240

20. Llewellyn C, Pollard A, Miners A, Richardson D, Fisher M, Cairns J, Smith H. Understanding patient choices for attending sexually transmitted infection testing services: a qualitative study. Sex Transm Infect, **2012**; 88: 504-509. doi: 10.1136/sextrans-2011-050344

21. Hologic Inc. Panther® System. https://www.hologic.com/hologic-products/diagnostic-solutions/panther-scalable-solutions/panther-system

22. Nicholls JE, Turner KME, North P, Ferguson R, May MT, Gough K, Macleod J, Muir P, Horner PJ. Cross-sectional study to evaluate Trichomonas vaginalis positivity in women tested for Neisseria gonorrhoeae and Chlamydia trachomatis, attending genitourinary medicine and primary care clinics in Bristol, South West England. Sex Transm Infect. **2018**;94: 93-99. doi: 10.1136/sextrans-2016-052942.

23. Mill Systems Limited. https://www.millsystems.com/millcare/

24. Simonton DK. Cross-sectional time series experiments: Some suggested statistical analyses. Psychol Bull, **1977**; 84: 489-502.

For peer review only - http://bmjopen.bmj.com/site/about/guidelines.xhtml

25. Bernal JL, Cummins S, Gasparrini A. The use of controls in interrupted time series studies of public health interventions. Int J Epi, **2018**; 47: 2082–2093. doi: 10.1093/ije/dyy135.
26. HM Treasury. GDP deflators at market prices, and money GDP December 2021 (Quarterly National Accounts). Available at: https://www.gov.uk/government/statistics/gdp-deflators-at-market-prices-and-money-gdp-december-2021-quarterly-national-accounts

26. HM Treasury. GDP deflators at market prices, and money GDP December 2021 (Quarterly National Accounts), 2021. Available at: https://www.gov.uk/government/statistics/gdp-deflators-at-market-prices-and-money-gdp-december-2021-quarterly-national-accounts

27. Joint Formulary Committee. British National Formulary. **2020**. Available at: http://www.medicinescomplete.com

28. Whitlock GG, Gibbons DC, Longford N, Harvey MJ, McOwan A, Adams EJ. Rapid testing and treatment for sexually transmitted infections improve patient care and yield public health benefits. Int J STD AIDS, **2018**; 29: 474–482.

29. Lorenc A, Kesten J, Brangan E, Horner PJ, Clarke M, Crofts M, Turner J, Muir P, Horwood J. What can be learnt from a qualitative evaluation of implementing a rapid sexual health testing, diagnosis and treatment service? BMJ Open, **2021**; 11: e050109. doi: 10.1136/bmjopen-2021-050109

30. de Vocht F, Katikireddi SV, McQuire C, Tilling K, Hickman M, Craig P. Conceptualising natural and quasi experiments in public health. BMC Med Res Methodol, **2021**; 21: 32. doi:10.1186/s12874-021-01224-x

For peer review only - http://bmjopen.bmj.com/site/about/guidelines.xhtml

Figure legends

Figure 1. Modelled outcome estimates for males. 'Panther' indicates the intervention-date representing the first week the Panther system was implemented for the male pathway: 12 November 2018.

Figure 2. Modelled outcome estimates for females. 'Panther for females' indicates the intervention-date representing the first week the Panther system was implemented for the female pathway: 29 May 2019.

Figure 3. Modelled estimates of staff capacity for males and females combined.

For peer review only

Figure 1. Modelled outcome estimates for males. ‘Panther’ indicates the intervention-date representing the first week the Panther system was implemented for the male pathway: 12 November 2018.

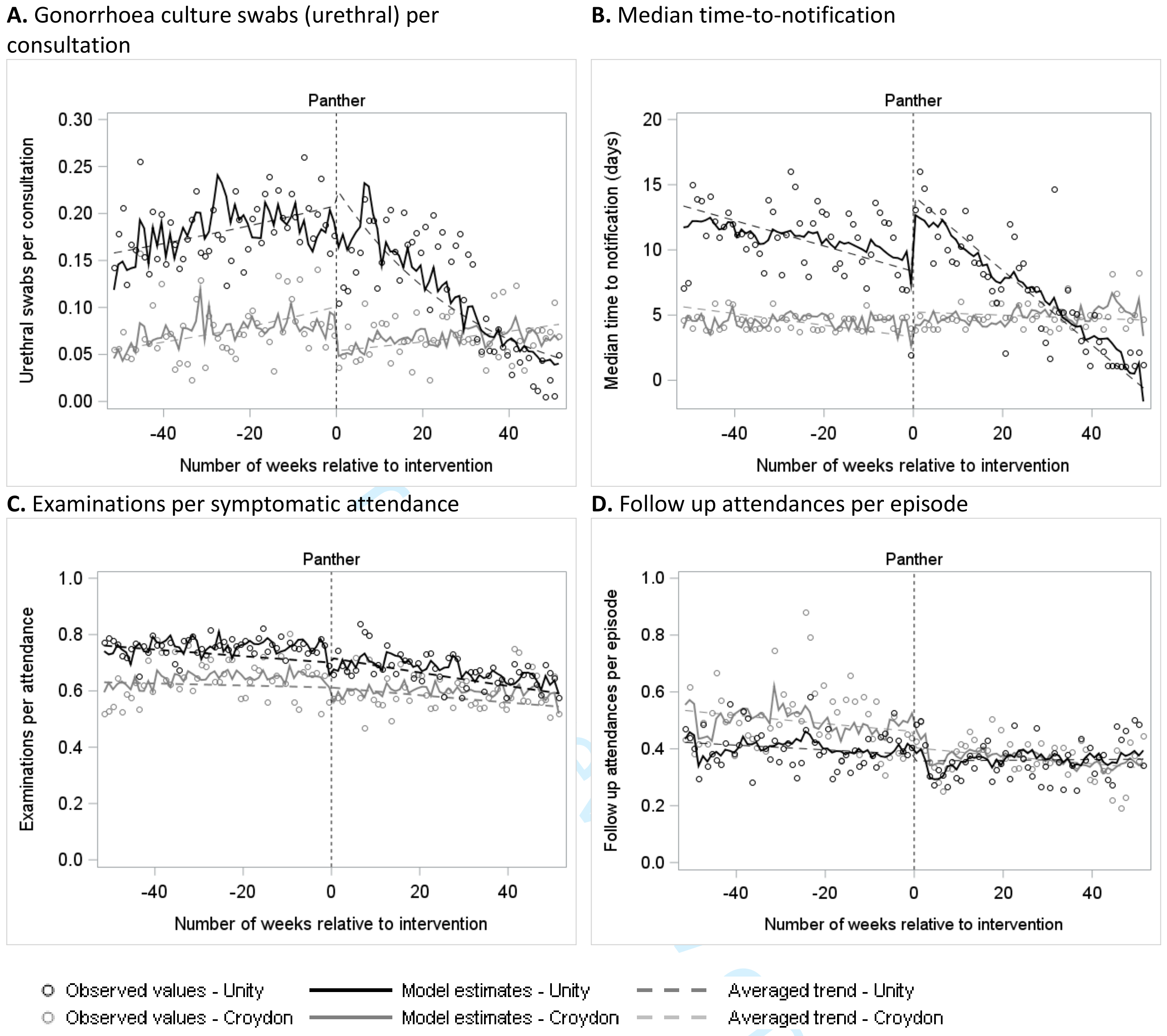

Figure 2. Modelled outcome estimates for females. 'Panther for females' indicates the intervention-date representing the first week the Panther system was implemented for the female pathway: 29 May 2019.

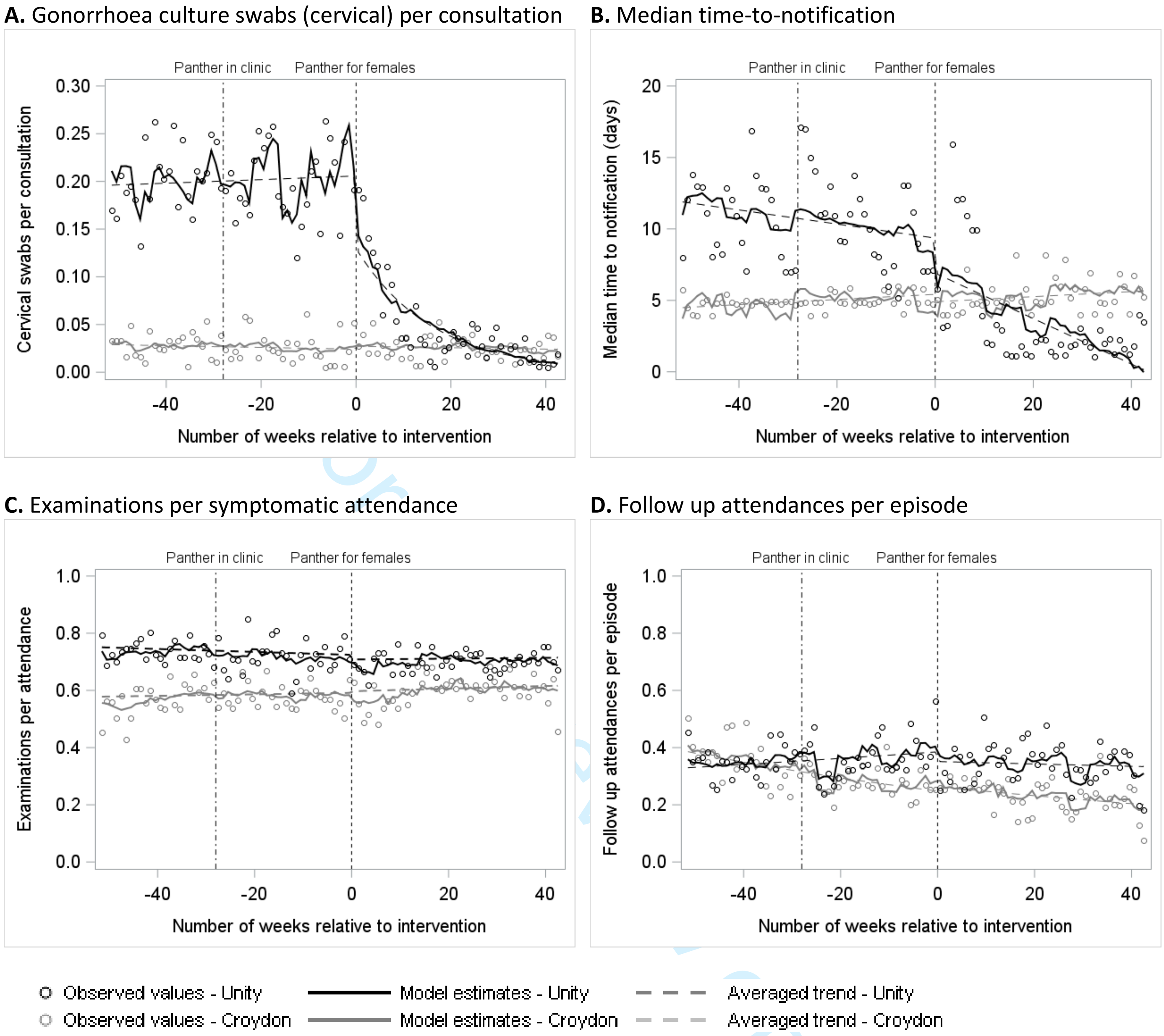

Figure 3. Modelled estimates of staff capacity for males and females combined.

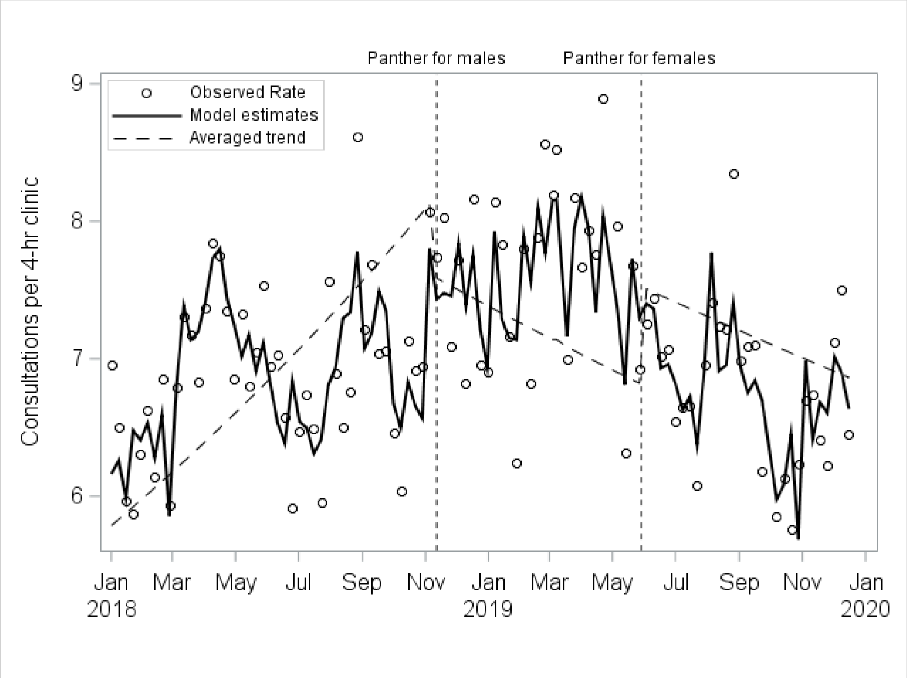

For peer review only

Supplementary material

The impact of rapid near-patient STI testing on service delivery outcomes: a controlled interrupted time series study

Scott R Walter, Joni Jackson, Gareth Myring, et al.

Figure S1. Male treatment pathway before (usual care pathway) and after (rapid pathway) implementation of the Panther rapid results system.

Figure S2. Overview of female rapid treatment pathway for asymptomatic and symptomatic patients before (usual care pathway) and after (rapid pathway) implementation of the Panther rapid results system.

Table S1. Definition of examination of any type based on a combination of two examination-related variables.

Figure S3. Diagrams to illustrate controlled interrupted time series variables for estimating A) changes at the time of intervention and B) changes in trends.

Table S2. Unit costs.

Table S3. Intervention-related model estimates for females and males from sensitivity analyses using generalised additive models.

Figure S4. Modelled outcome estimates for males based on sensitivity analyses using generalised additive models.

Figure S5. Modelled outcome estimates for females based on sensitivity analyses using generalised additive models.

Figure S6. Modelled estimates of staff capacity for males and females combined.

Definition of complex cases

For peer review only

Figure S1. Male treatment pathway before (usual care pathway) and after (rapid pathway) implementation of the Panther rapid results system. Reproduced from: Lorenc A, Kesten J, Brangan E, Horner PJ, Clarke M, Crofts M, Turner J, Muir P, Horwood J. What can be learnt from a qualitative evaluation of implementing a rapid sexual health testing, diagnosis and treatment service? BMJ Open, 2021; 11: e050109. doi: 10.1136/bmjopen-2021-050109.

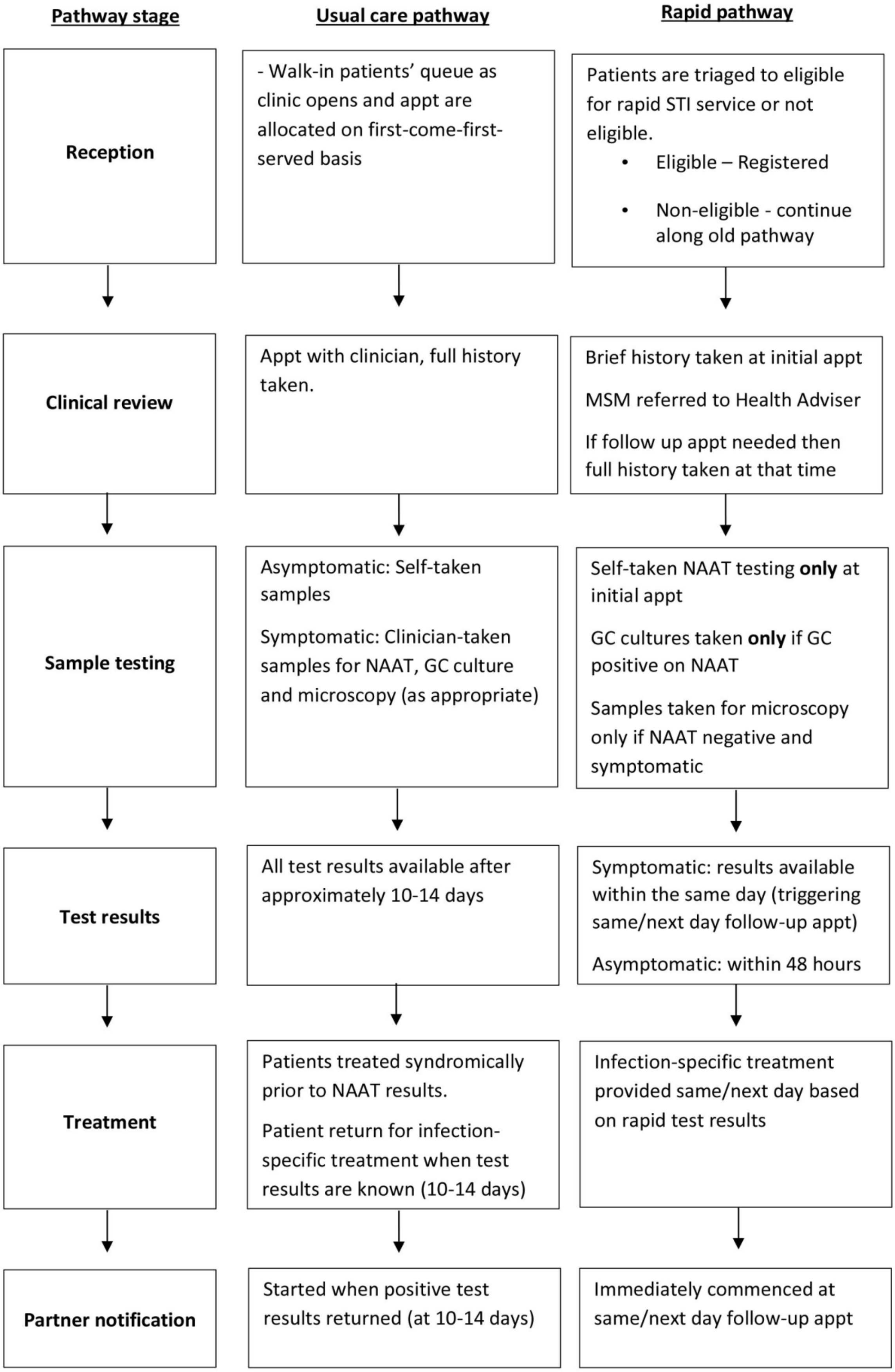

Figure S2. Overview of female rapid treatment pathway for asymptomatic and symptomatic patients before (usual care pathway) and after (rapid pathway) implementation of the Panther rapid results system. NAAT = Nucleic Acid Amplification Test GC = Gonorrhoea CT= Chlamydia, TV= *Trichomonas vaginalis*

Pathway stage	Usual care pathway	Rapid pathway: asymptomatic	Rapid pathway: symptomatic
**Reception**	Walk-in patients' queue as clinic opens and appointments are allocated on first-come-first-served basis.	Patients are triaged to rapid STI service or not:<br>● Asymptomatic and Eligible = Registered<br>● Asymptomatic and Non-eligible = continue along modified usual care pathway	Patients triaged to rapid STI service or not:<br>● Symptomatic and Eligible = Registered<br>● Symptomatic and non-eligible = modified usual care pathway (GC/CT and TV NAAT results <48 hrs) GC cultures only taken if GC NAAT positive or patient receives treatment for GC before results of GC NAAT available
**Clinical review**	Appt. with clinician, full history taken.	Brief history taken at initial appt.<br>If follow up appt. needed then full history taken at that time	History taken and no indications for visual inspection or pelvic examination required.<br>If follow up appt. needed then full history taken at that time
**Sample testing**	● Asymptomatic: Self-taken samples<br>● Symptomatic: Clinician-taken samples for CT/GC NAAT. If vaginal symptoms or pelvic pain then speculum exam and swabs taken for microscopy, TV and GC culture (as appropriate).	Self-taken NAAT testing ***only*** at initial appt<br>GC cultures taken ***only*** if GC positive on NAAT or if treatment as GC contact within 2 weeks of sexual intercourse with contact	Self-taken:<br>● Vaginal CT/GC/TV NAAT<br>● Vaginal swab for Gram staining<br>GC cultures taken ***only*** if GC positive on NAAT or if treatment as GC contact within 2 weeks of sexual intercourse with contact.
**Test results**	All test results available after approximately 10-14 days.	Results available within 48 hours.	Results of vaginal microscopy available in 20 mins and patient is then reviewed by clinician CT/GCTV NAAT results available within 48 hours
**Treatment**	Patients treated syndromically prior to NAAT results.<br>Patients return for infection-specific treatment when test results are known (10-14 days).	Infection-specific treatment provided same/next day based on rapid test results.	Infection-specific treatment provided same/next day based on rapid test results.
**Partner notification**	Started when positive test results returned (at 10-14 days).	Immediately commenced at same/next day follow-up appt.	Immediately commenced at same/next day follow-up appt.

For peer review only - http://bmjopen.bmj.com/site/about/guidelines.xhtml

Table S1. Definition of examination of any type based on a combination of two examination-related variables.

Categorical exam variable	Free text exam variable	Define as exam – MALES	Define as exam - FEMALES
Yes	Notes indicating exam	Yes	Yes
Yes	Missing	Yes	Yes
No	Notes indicating exam	Yes	Yes
No	Notes indication NO exam		
No	Missing		
External only	Notes indicating exam	Yes	Yes
External only	Notes indication NO exam		
External only	Missing		Yes
Speculum and external	Notes indicating exam	Yes	Yes
Speculum and external	Notes indication NO exam		
Speculum and external	Missing		Yes
Missing	Notes indicating exam	Yes	Yes
Missing	Notes indication NO exam		
Missing	Missing		

Notes: The categorical exam variable was intended for use with female patients but was sometimes used for males.

For peer review only

Figure S3. Diagrams to illustrate controlled interrupted time series variables for estimating A) changes at the time of intervention and B) changes in trends.

A. Changes at the time of intervention

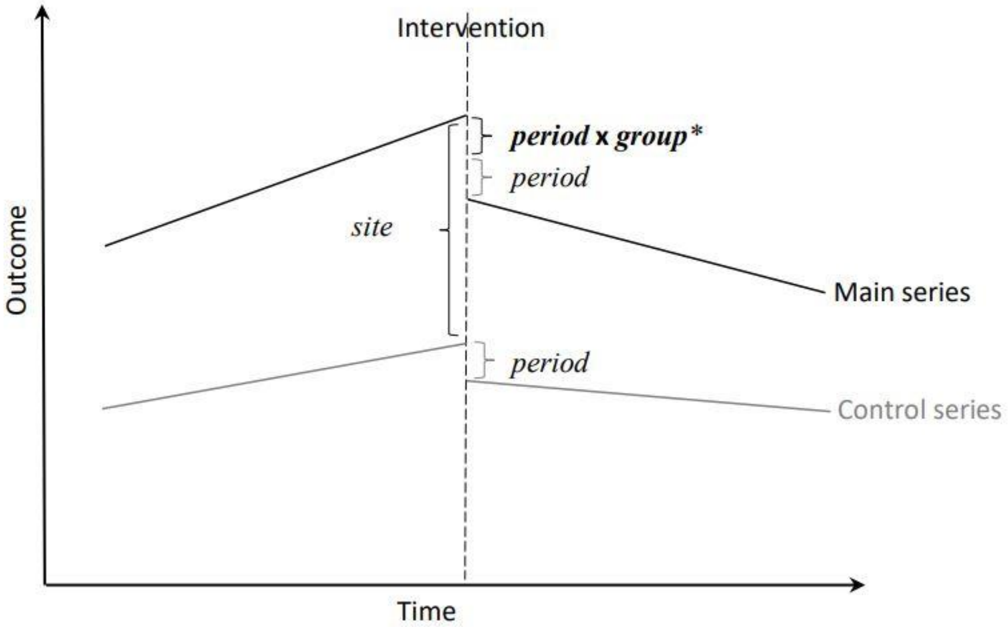

B. Trend changes

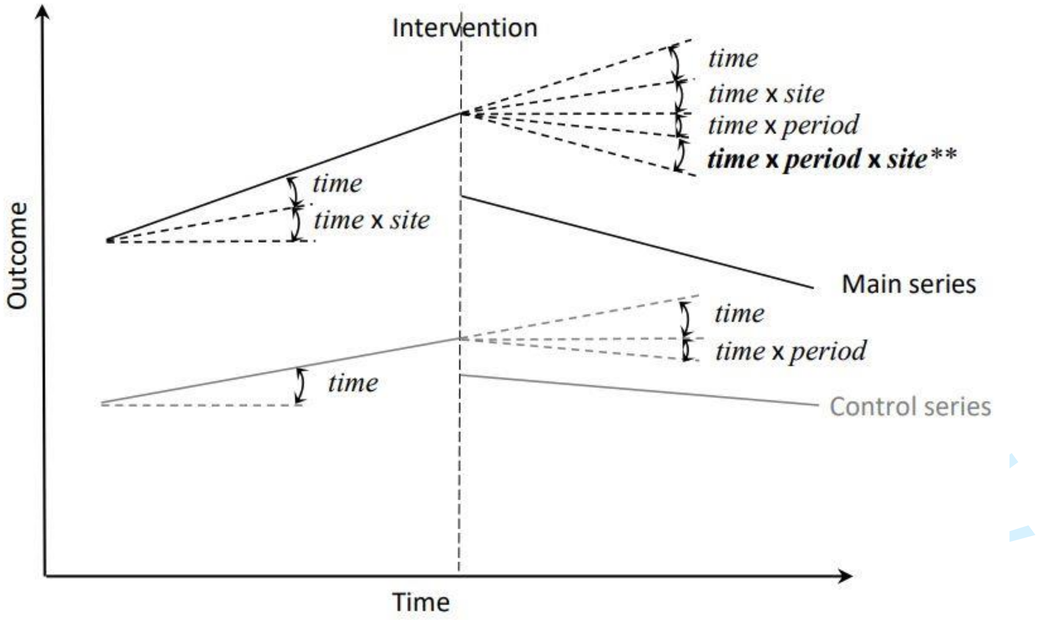

Note: *site* is a binary variable indicating either intervention or control sites; *period* is also binary indicating pre- or post-intervention periods; *time* is a continuous variable consecutively numbering each time unit (weeks in this study) with *time*=0 centred at the intervention

* *period* x *site* represents change in the intervention site at the time of intervention over and above any changes in the control site

** *time* x *period* x *site* represents change in trend for the intervention site over and above any trend changes in the control site

Table S2. Unit costs.

Unit costs of postal kit tests, and staff time[12] were from the literature and inflated to 2021 values using a UK government GDP deflator[26]. Local unit costs of diagnostic tests were provided by Unity SHS. Treatment costs were from the British National Formulary[27].

Resource use	Unit cost
**Tests**	
Lab CT/GC test	£8.10
POCT CT/GC	£9.48
GC culture swab	£6.13
Male postal kit returned	£4.44
Male postal kit not returned	£3.61
Female postal kit returned	£4.08
Female postal kit not returned	£3.24
**Consultation staff time**	
Follow-up	£9.33
Male non-complex	£29.03
Male complex	£46.54
MSM complex	£42.97
Female non-complex	£29.03
Female complex	£52.26
**Treatment**	
13.5mg Levonorgestrel IUS	£69.22
6mg norelgestromin and 600micrograms ethinylestradiol	£19.51
Aciclovir 400 mg (tds for 5 Days)	£0.79
Aciclovir 400mg (bd for 6 months)	£17.64
Aciclovir 400mg (bd for 3 months)	£8.82
Aciclovir 800mg (tds for 2 days)	£0.59
Amoxicillin 250 mg tds for 5 days	£0.98
Amoxicillin 500mg	£1.01
Anusol Cream	£2.49
Anusol Ointment	£2.49
Anusol Suppositories	£1.74
Aqueous Cream BP 100g Tube	£0.77
Aqueous Cream BP 500g Tub	£3.85
Azithromycin 1g (2 x 500mg tablets)	£0.81
Azithromycin 1g (4 x 250mg capsules)	£1.24
Azithromycin 1g stat, then 500mg od for 2 days	£1.21
Azithromycin 1g stat, then 500mg od for 4 days	£2.42
Azithromycin 2g o stat	£1.62
Benzathine Benzylpenicillin 2.4 million units on day 0	£9.50
Benzathine benzylpenicillin 2.4 million units at day 7	£9.50
Benzathine benzylpenicillin 2.4 million units at day14	£9.50
Betamethasone Valerate 0.1% w/w Cream	£1.47
Betamethasone Valerate 0.1% w/w Ointment	£1.84
Betamethasone Valerate Ointment (Betnovate RD)	£1.84
Cefixime 400mg (2 x 200mg)	£26.46
Ceftriaxone 1g	£3.62
Ceftriaxone 500 mg (2 x 250mg vials)	£4.60
Chlorphenamine	£2.21
Cilest 63 tablet pack	£4.65

For peer review only

Ciprofloxacin 500mg (2 x 250mg)	£0.31
Clindamycin 300mg bd for 7 days	£17.84
Clindamycin phosphate vaginal cream	£10.86
Clobetasol Propinate (0.05% w/w) Cream (Dermovate)	£2.69
Clobetasol Propionate (0.05% w/w) Ointment	£2.69
Clobetasone Butyrate Cream (Eumovate)	£1.86
Clobetasone Butyrate Ointment (Eumovate)	£1.86
Clobetasone Butyrate, Calcium oxtertracycline & Nystatin Cream (Trimovate)	£12.45
Clotrimazole 100mg Pessary	£0.64
Clotrimazole 200mg Pessary	£1.14
Clotrimazole 500 mg Pessary	£6.99
Clotrimazole Cream 1%	£1.36
Co-Amoxiclav 250/125 (contains PENICILLIN)	£2.03
Co-amxoxiclav 500/125 (contains PENICILLIN)	£2.53
Crotamiton 10% w/w cream	£2.50
Dermol Lotion 500	£6.04
Desogestrel 75 micrograms	£2.26
Doxycycline 100mg (bd for 14 days)	£3.67
Doxycycline 100mg (bd for 21 days)	£5.51
Doxycycline 100mg (bd for 28 days)	£7.35
Doxycycline 100mg (bd for 7 days)	£2.26
Doxycycline 200mg bd for 4 weeks	£5.51
Emtricitabine 200mg & Tenofovir Disproxil 245mg	£106.00
Emtricitabine 200mg & Tenofovir Disproxil 245mg (3 days)	£10.60
Emulsifying Ointment	£4.82
Erythromycin 250 mg	£8.95
Estradiol 0.5g gel	£5.08
Estradiol 1.0mg gel	£5.85
Estradiol 10 micrograms vaginal tablet	£16.72
Femodene 63 tablet pack	£6.73
Flucloxacillin	£1.41
Fluconazole 150mg	£0.91
Fusidic acid cream	£1.92
GENTAMICIN 240mg for IM injection	£4.13
GYNAEFIX IUD	£27.11
Gardasil 0.5ml - First Dose	£86.50
Gardasil 0.5mls - Second Dose	£86.50
Gardasil 0.5mls - Third Dose	£86.50
Gedarel 20/150	£5.08
Gedarel 30/150	£5.08
Hepatitis A & B Combined Vaccine (adult)	£31.18
Hepatitis A Vaccine (2nd at 6 months)	£16.77
Hepatitis A vaccine Day 0	£16.77
Hepatitis B Vaccine (final at 6 months)	£12.20
Hepatitis B Vaccine - Dose 1 - 10mcg or 20mcg	£12.20
Hepatitis B Vaccine - Dose 2 - 10mcg or 20mcg	£12.20
Hepatitis B Vaccine - Dose 3 - 10mcg or 20mcg	£12.20
Hepatitis B Vaccine - Dose 4 - 10mcg or 20mcg	£12.20
Hepatitis B Vaccine - Extra Dose - 10mcg or 20 mcg	£12.20
Hepatitis B Vaccine 10 mcg 1 month	£12.20

For peer review only

Hepatitis B Vaccine 10 mcg 12 months	£12.20
Hepatitis B Vaccine Day 7	£12.20
Hepatits B Vaccine Day 21	£12.20
Hydro-Caine 6mls	£10.50
Hydrocortisone Cream 1%	£1.40
Hydrocortisone Ointment 1%	£1.59
Ibuprofen 200mg	£1.03
Imiquimod 5%	£48.60
Itraconazole 100mg	£3.29
Levonorgestral and Ethinylestradiol 150microgram/30microgram	£2.60
Levonorgestrel 1.5 mg	£3.65
Levonorgestrel 30 micrograms	£0.92
Levosert 52mgs IUS	£66.00
Lidocaine 4% w/w cream	£2.98
Lidocaine 5% m/m Ointment	£8.28
Lidocaine HCL 1% in 2 mls injection	£0.25
Lidocaine HCL 1% in 3.5 mls injection	£0.30
Lidocaine HCL 1% in 5 mls injection	£0.30
Lidocaine HCL 1% in 8mls for IM inj (with IM penicillin) second dose	£0.10
Lidocaine HCL 1% in 8mls for IM injection (with IM penicillin for syphilis)	£0.10
Lidocaine HCL 1% in 8mls for IM injection (with IM penicillin) third dose	£0.10
Lidocaine HCL 2% in 2 mls injection	£0.27
Lidocaine HCL 2% in 5 mls injection	£0.32
Lignocaine 2% Gel	£2.99
Loestrin 20 63 Tablet Pack	£1.99
Loestrin 30 63 Tablet Pack	£1.99
Logynon	£2.60
Marvelon 63 Tablet Pack	£7.10
Mebendazole 100mg	£2.66
Medroxyprogesterone Acetate 104mg in 0.65mls sub cutaneous	£6.90
Medroxyprogesterone Acetate 150mg in 1ml	£6.01
Mefenamic Acid 250mg	£8.17
Mepivacaine Hydrochloride 3%	£0.44
Mepivicaine 3% in 2.2mls	£0.44
Mercilon 63 Tablet Pack	£8.44
Metronidazole 0.75% Vaginal Gel	£4.31
Metronidazole 2g stat dose (400 mg x 5)	£0.52
Metronidazole 400mg (bd for 5 days)	£1.03
Metronidazole 400mg bd for 10 days	£2.07
Miconazole Nitrate 2%w/w, hydrocortisone 1%w/w Cream (Daktocourt)	£2.49
Miconazole Nitrate Cream 20mg/g (Gyno-Daktarin)	£4.33
Miconazole nitrate 20mg per g	£4.33
Millinette 20/75	£5.41
Millinette 30/75	£4.12
Mini TT 380	£12.46
Mirena 52mg IUS	£88.00
Moxifloxacin 400mg od for 10 days	£19.08
Moxifloxacin 400mg od for 14 days	£26.71
Nexplanon 68mg implant	£83.43
Nitrofurantoin 50mg o qds 7 days	£5.08
Nitrofurantoin 50mg o qds for 3 days	£2.18

For peer review only

Nonoxinol-9	£11.00
Norethisterone 350 micrograms	£2.10
Norethisterone 350 micrograms 84 Tablet Pack	£2.10
Norimin 63 Tablet Pack	£2.28
Nova T 380	£15.20
Ofloxacin 200mg (one tablet twice daily for 14 days)	£12.54
Ofloxacin 200mg (one tablet twice daily for 7 days)	£6.27
Ofloxacin 200mg (two tablets twice daily for 14 days)	£25.09
Paediatric Hepatitis B Vaccine - Dose 1 - 10mcg	£12.20
Paediatric Hepatitis B Vaccine - Dose 2 - 10 mcg	£12.20
Paediatric Hepatitis B Vaccine - Dose 3 - 10 mcg	£12.20
Paediatric Hepatitis B Vaccine - Dose 4 - 10mcg	£12.20
Paracetamol 500mg	£0.86
Permethrin 5% w/w cream	£8.54
Podophyllotoxin 0.15% Cream	£17.83
Podophyllotoxin 0.5% Solution	£14.49
Raltegravir 400 mg bd for 3 days	£47.14
T- Safe 380A QL	£10.55
TT 380 Slimline	£12.46
Terbinafine Hydrochloride 1% Cream	£2.39
Trimethoprim 200mg	£1.16
Ulipristal Acetate 30mg	£14.05
Xylocaine 1% with adrenaline 1 :200,000	£1.77

For peer review only

Table S3. Intervention-related model estimates for females and males from sensitivity analyses using generalised additive models.

Outcome	Percent change at time of intervention (95% CI)	P-value for post-panther non-linearity of Unity data
**MALES – 12th November 2018**		
Gonorrhoea culture swabs per consultation	-16.6% (-30.1%, -0.5%)	<0.001
Time to notification	+63.3% (+31.4%, +102.8%)	0.03
**FEMALES – 29th May 2019**		
Gonorrhoea culture swabs per consultation	-11.1% (-29.8%, +12.6%)	<0.001
Time to notification	-14.5% (-34.0%, +10.8%)	<0.001

For peer review only

Figure S4. Modelled outcome estimates for males based on sensitivity analyses using generalised additive models. Both the overall time trend and the post-panther Unity trend were estimated as splines with three degrees of freedom. All other covariates treated as in the main analysis.

A. Gonorrhoea culture swabs (urethral) per consultation

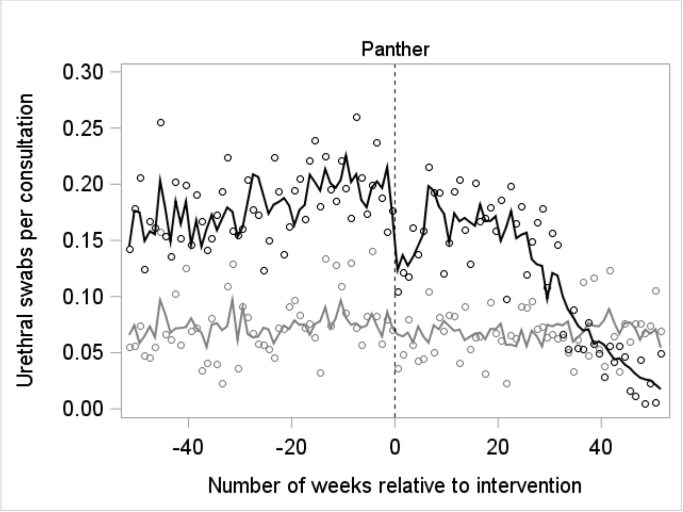

B. Median time to notification

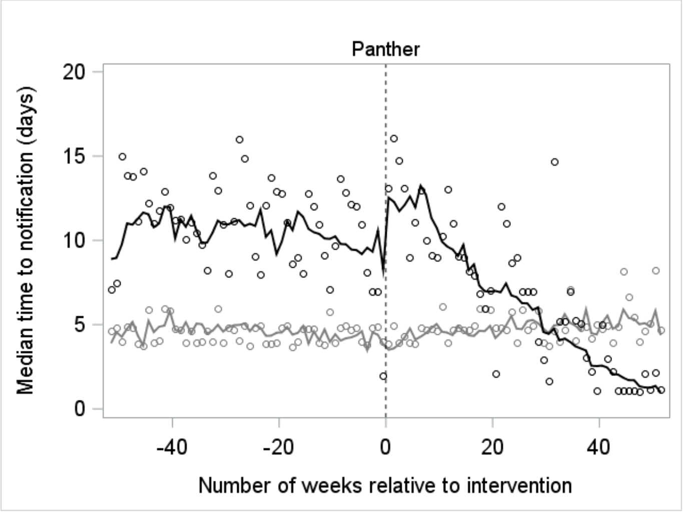

C. Examinations per symptomatic attendance

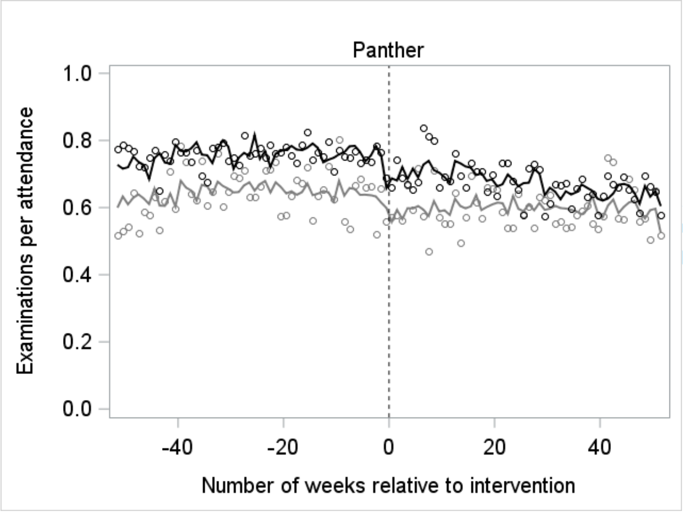

D. Follow up attendances per episode

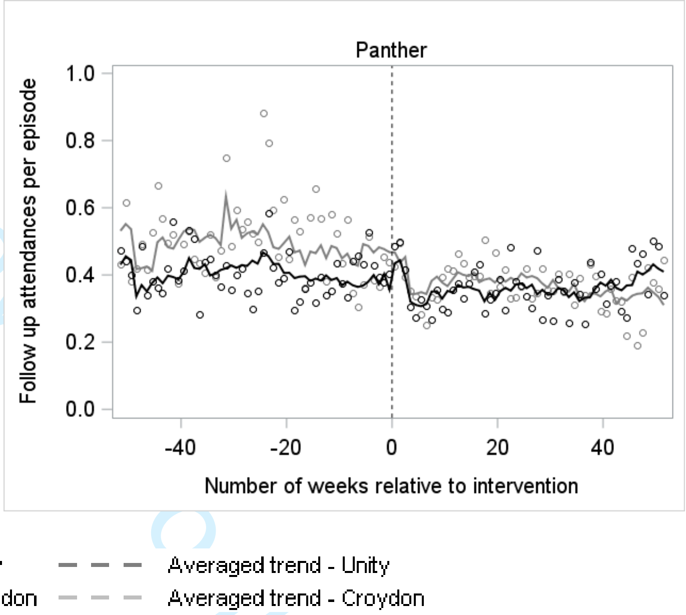

Figure S5. Modelled outcome estimates for females based on sensitivity analyses using generalised additive models. Both the overall time trend and the post-panther Unity trend were estimated as splines with three degrees of freedom. All other covariates treated as in the main analysis.

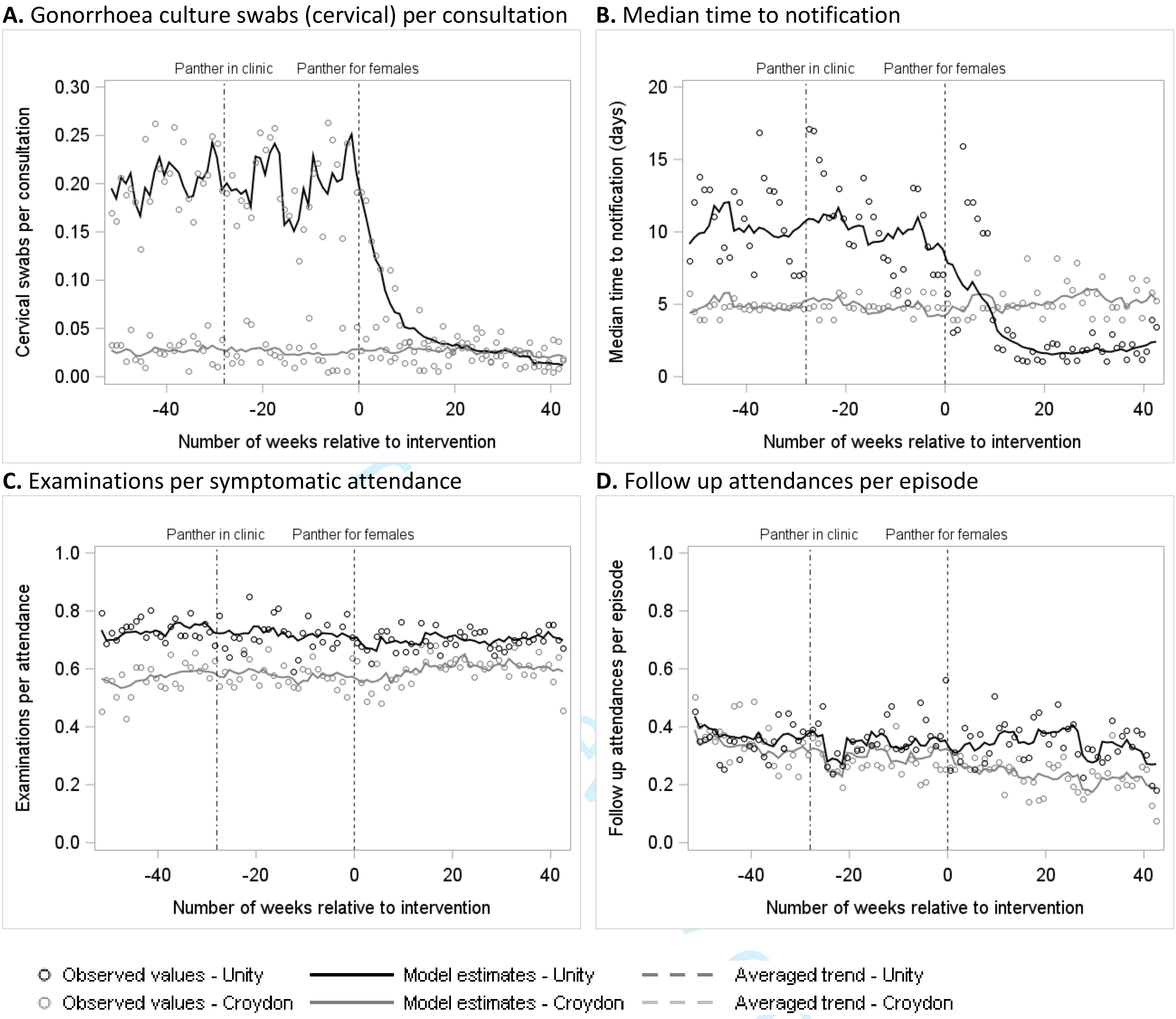

Figure S6. Modelled estimates of staff capacity for males and females combined. Time trends modelled with splines to allow for non-linearity. All other covariates treated as in the main analysis.

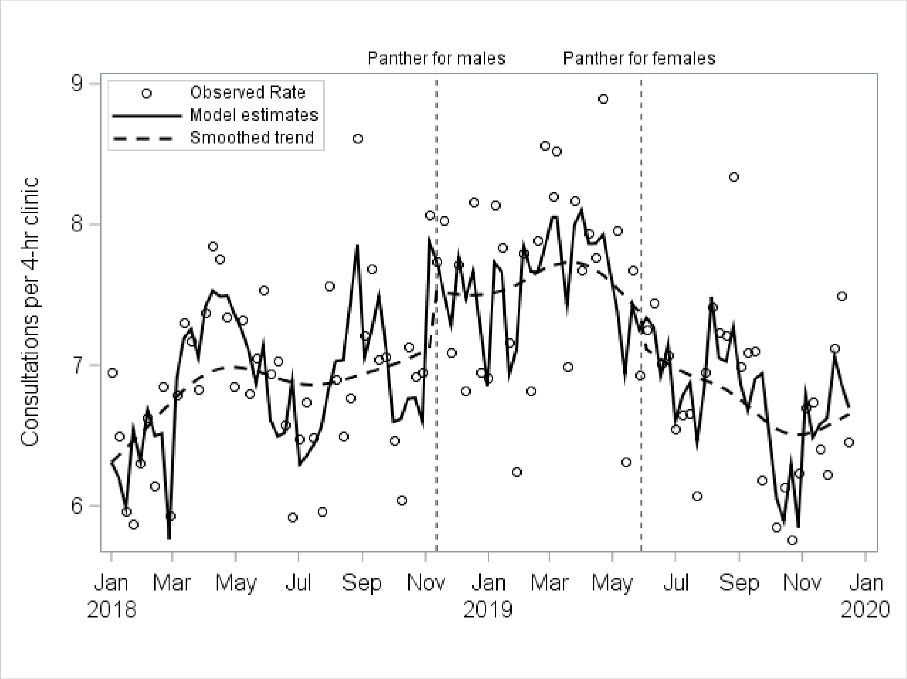

For peer review only

Definition of complex cases

Criteria for all patients:

a. Patients under 18 years of age
b. Have been/are currently exposed to child sexual exploitation, domestic violence, sexual assault
c. Has a current record of substance misuse
d. Has a current diagnosis of syphilis
e. Has current multiple diagnoses clinical diagnoses (GUMCAD coding B &/or C)
f. Has a history of/current diagnosis of genital herpes or had a swab taken for genital herpes
g. Has had post exposure prophylaxis after sexual exposure to HIV (PEPSE)
h. Needed an interpreter/use of translation service
i. Has current diagnosis of D2B on GUMCAD

Additional criteria for females:

i. Receive contraceptive care
ii. experienced pelvic pain, dyspareunia or post coital bleeding
iii. are pregnant
iv. experienced female genital mutilation.

Additional criteria for males:

v. are bisexual
vi. has sex with men
vii. Experienced testicular pain
viii. has a history/current record of chronic pelvic syndrome

For peer review only

The RECORD statement – checklist of items, extended from the STROBE statement, that should be reported in observational studies using routinely collected health data.

	Item No.	STROBE items	Location in manuscript where items are reported	RECORD items	Location in manuscript where items are reported
**Title and abstract**					
	1	(a) Indicate the study's design with a commonly used term in the title or the abstract (b) Provide in the abstract an informative and balanced summary of what was done and what was found	(a) p.1<br>(b) p.2	RECORD 1.1: The type of data used should be specified in the title or abstract. When possible, the name of the databases used should be included.<br><br>RECORD 1.2: If applicable, the geographic region and timeframe within which the study took place should be reported in the title or abstract.<br><br>RECORD 1.3: If linkage between databases was conducted for the study, this should be clearly stated in the title or abstract.	1.1, abstract p.2<br><br>1.2, abstract p.2<br><br>N/A
**Introduction**					
Background rationale	2	Explain the scientific background and rationale for the investigation being reported			Introduction pp.4-5
Objectives	3	State specific objectives, including any prespecified hypotheses			End of introduction p.5
**Methods**					
Study Design	4	Present key elements of study design early in the paper			Section 2.1, p.5
Setting	5	Describe the setting, locations, and relevant dates, including periods of recruitment, exposure, follow-up, and data collection			Methods pp.5-7

For peer review only

Participants	6	*(a) Cohort study* - Give the eligibility criteria, and the sources and methods of selection of participants. Describe methods of follow-up<br>*Case-control study* - Give the eligibility criteria, and the sources and methods of case ascertainment and control selection. Give the rationale for the choice of cases and controls<br>*Cross-sectional study* - Give the eligibility criteria, and the sources and methods of selection of participants<br><br>*(b) Cohort study* - For matched studies, give matching criteria and number of exposed and unexposed<br>*Case-control study* - For matched studies, give matching criteria and the number of controls per case		RECORD 6.1: The methods of study population selection (such as codes or algorithms used to identify subjects) should be listed in detail. If this is not possible, an explanation should be provided.<br><br>RECORD 6.2: Any validation studies of the codes or algorithms used to select the population should be referenced. If validation was conducted for this study and not published elsewhere, detailed methods and results should be provided.<br><br>RECORD 6.3: If the study involved linkage of databases, consider use of a flow diagram or other graphical display to demonstrate the data linkage process, including the number of individuals with linked data at each stage.	Table 1<br><br><br><br><br>N/A<br><br><br><br><br>N/A
Variables	7	Clearly define all outcomes, exposures, predictors, potential confounders, and effect modifiers. Give diagnostic criteria, if applicable.		RECORD 7.1: A complete list of codes and algorithms used to classify exposures, outcomes, confounders, and effect modifiers should be provided. If these cannot be reported, an explanation should be provided.	Outcomes – table 1<br>Confounders – section 2.5, pp.7-8
Data sources/ measurement	8	For each variable of interest, give sources of data and details of methods of assessment (measurement).<br>Describe comparability of assessment methods if there is more than one group			Sections 2.4 & 2.5, pp.6-9

For peer review only

Bias	9	Describe any efforts to address potential sources of bias			Section 2.5, pp.7-8
Study size	10	Explain how the study size was arrived at			Section 2.4, p.6
Quantitative variables	11	Explain how quantitative variables were handled in the analyses. If applicable, describe which groupings were chosen, and why			Sections 2.5 and 2.6, pp.7-9 Supplement Table S1
Statistical methods	12	(a) Describe all statistical methods, including those used to control for confounding<br>(b) Describe any methods used to examine subgroups and interactions<br>(c) Explain how missing data were addressed<br>(d) *Cohort study* - If applicable, explain how loss to follow-up was addressed<br>*Case-control study* - If applicable, explain how matching of cases and controls was addressed<br>*Cross-sectional study* - If applicable, describe analytical methods taking account of sampling strategy<br>(e) Describe any sensitivity analyses			Sections 2.5 and 2.6, pp.7-9
Data access and cleaning methods		..		RECORD 12.1: Authors should describe the extent to which the investigators had access to the database population used to create the study population.	12.1: Section 2.4, p.6<br><br>12.2: Section 2.4, p.7

For peer review only

				RECORD 12.2: Authors should provide information on the data cleaning methods used in the study.	
Linkage		..		RECORD 12.3: State whether the study included person-level, institutional-level, or other data linkage across two or more databases. The methods of linkage and methods of linkage quality evaluation should be provided.	N/A
**Results**					
Participants	13	(a) Report the numbers of individuals at each stage of the study (*e.g.*, numbers potentially eligible, examined for eligibility, confirmed eligible, included in the study, completing follow-up, and analysed)<br>(b) Give reasons for non-participation at each stage.<br>(c) Consider use of a flow diagram		RECORD 13.1: Describe in detail the selection of the persons included in the study (*i.e.,* study population selection) including filtering based on data quality, data availability and linkage. The selection of included persons can be described in the text and/or by means of the study flow diagram.	Results, first paragraph, p.9
Descriptive data	14	(a) Give characteristics of study participants (*e.g.*, demographic, clinical, social) and information on exposures and potential confounders<br>(b) Indicate the number of participants with missing data for each variable of interest<br>(c) *Cohort study* - summarise follow-up time (*e.g.*, average and total amount)			Results, first paragraph p.9 and Table 2.
Outcome data	15	*Cohort study* - Report numbers of outcome events or summary measures over time<br>*Case-control study* - Report numbers in each exposure			Table 2

For peer review only

		category, or summary measures of exposure<br>*Cross-sectional study* - Report numbers of outcome events or summary measures			
Main results	16	(a) Give unadjusted estimates and, if applicable, confounder-adjusted estimates and their precision (e.g., 95% confidence interval). Make clear which confounders were adjusted for and why they were included<br>(b) Report category boundaries when continuous variables were categorized<br>(c) If relevant, consider translating estimates of relative risk into absolute risk for a meaningful time period			Sections 3.1-3.3 (pp.9-11) and Tables 3 & 4.
Other analyses	17	Report other analyses done—e.g., analyses of subgroups and interactions, and sensitivity analyses			Sections 3.1-3.3 (pp.9-11) and supplement table S3, figures S4-S6.
**Discussion**					
Key results	18	Summarise key results with reference to study objectives			pp.11-13
Limitations	19	Discuss limitations of the study, taking into account sources of potential bias or imprecision. Discuss both direction and magnitude of any potential bias		RECORD 19.1: Discuss the implications of using data that were not created or collected to answer the specific research question(s). Include discussion of misclassification bias, unmeasured confounding, missing data, and changing eligibility over time, as they pertain to the study being reported.	Section 4.1, pp13-14.
Interpretation	20	Give a cautious overall interpretation of results considering objectives,			Section 4.2, pp.14-15

For peer review only

		limitations, multiplicity of analyses, results from similar studies, and other relevant evidence			
Generalisability	21	Discuss the generalisability (external validity) of the study results			Section 4.2, pp.14-15
**Other Information**					
Funding	22	Give the source of funding and the role of the funders for the present study and, if applicable, for the original study on which the present article is based			p.16
Accessibility of protocol, raw data, and programming code		..		RECORD 22.1: Authors should provide information on how to access any supplemental information such as the study protocol, raw data, or programming code.	See data statement, p.16

*Reference: Benchimol EI, Smeeth L, Guttmann A, Harron K, Moher D, Petersen I, Sørensen HT, von Elm E, Langan SM, the RECORD Working Committee. The REporting of studies Conducted using Observational Routinely-collected health Data (RECORD) Statement. *PLoS Medicine* 2015; in press.

*Checklist is protected under Creative Commons Attribution (CC BY) license.

For peer review only

BMJ Open

The impact of rapid near-patient STI testing on service delivery outcomes in an integrated sexual health service in the United Kingdom: a controlled interrupted time series study

Journal:	*BMJ Open*
Manuscript ID	bmjopen-2022-064664.R1
Article Type:	Original research
Date Submitted by the Author:	03-Oct-2022
Complete List of Authors:	Walter, Scott; University of Bristol Medical School, NIHR Applied Research Collaboration West<br>Jackson, Joni; NIHR ARC West, ARC West; University of Bristol Medical School, Populations Health Sciences<br>Myring, Gareth; University of Bristol School of Social and Community Medicine<br>Redaniel, Maria Theresa; University of Bristol, NIHR CLAHRC West; University of Bristol, School of Social and Community Medicine<br>Margelyte, Ruta; University of Bristol, School of Social and Community Medicine; NIHR Collaboration for Leadership in Applied Health Research and Care West,<br>Gardiner, Rebecca; University Hospitals Bristol and Weston NHS Foundation Trust, Unity Sexual Health; University Hospitals Bristol and Weston NHS Foundation Trust, Bristol Haematology and Oncology Centre<br>Clarke, Michael; University Hospitals Bristol and Weston NHS Foundation Trust, Unity Sexual Health<br>Crofts, Megan; University Hospitals Bristol and Weston NHS Foundation Trust, Unity Sexual Health<br>McLeod, Hugh; University of Bristol<br>Hollingworth, William; University of Bristol, School of Social and Community Medicine<br>Phillips, David; Croydon University Hospital, Croydon Sexual Health<br>Muir, Peter; Public Health England, Specialist Virology Centre, Public Health Laboratory Bristol<br>Steer, Jonathan; Public Health England, Bristol Public Health Laboratory<br>Turner, Jonathan; Public Health England, Bristol Public Health Laboratory<br>Horner, Paddy; University of Bristol, School of Social and Community Medicine; University Hospitals Bristol NHS Fouindation Trust, Bristol Sexual Health Centre<br>De Vocht, Frank; University of Bristol,
**Primary Subject Heading**:	Sexual health
Secondary Subject Heading:	Health services research
Keywords:	SEXUAL MEDICINE, Epidemiology < INFECTIOUS DISEASES, HEALTH ECONOMICS

SCHOLARONE™
Manuscripts

I, the Submitting Author has the right to grant and does grant on behalf of all authors of the Work (as defined in the below author licence), an exclusive licence and/or a non-exclusive licence for contributions from authors who are: i) UK Crown employees; ii) where BMJ has agreed a CC-BY licence shall apply, and/or iii) in accordance with the terms applicable for US Federal Government officers or employees acting as part of their official duties; on a worldwide, perpetual, irrevocable, royalty-free basis to BMJ Publishing Group Ltd ("BMJ") its licensees and where the relevant Journal is co-owned by BMJ to the co-owners of the Journal, to publish the Work in this journal and any other BMJ products and to exploit all rights, as set out in our licence.

The Submitting Author accepts and understands that any supply made under these terms is made by BMJ to the Submitting Author unless you are acting as an employee on behalf of your employer or a postgraduate student of an affiliated institution which is paying any applicable article publishing charge ("APC") for Open Access articles. Where the Submitting Author wishes to make the Work available on an Open Access basis (and intends to pay the relevant APC), the terms of reuse of such Open Access shall be governed by a Creative Commons licence – details of these licences and which Creative Commons licence will apply to this Work are set out in our licence referred to above.

Other than as permitted in any relevant BMJ Author's Self Archiving Policies, I confirm this Work has not been accepted for publication elsewhere, is not being considered for publication elsewhere and does not duplicate material already published. I confirm all authors consent to publication of this Work and authorise the granting of this licence.

For peer review only

The impact of rapid near-patient STI testing on service delivery outcomes in an integrated sexual health service in the United Kingdom: a controlled interrupted time series study

Scott R Walter[1,2], Joni Jackson[1,2], Gareth Myring[1,2], Maria Theresa Redaniel[1,2], Ruta Margelyte[1,2], Rebecca Gardiner[3,4], Michael D Clarke[4], Megan Crofts[4], Hugh McLeod[1,2], William Hollingworth[1,2], David Phillips[5], Peter Muir[6,7], Jonathan Steer[6], Jonathan Turner[6], Paddy J Horner[2,3,7]*, Frank de Vocht[1,2]*

*joint last authors

Affiliations

1. National Institute for Health Research, Applied Research Collaboration West (NIHR ARC West) University Hospitals Bristol and Weston NHS Foundation Trust, Bristol, UK.
2. Population Health Sciences, Bristol Medical School, University of Bristol, Bristol, UK.
3. Unity Sexual Health, University Hospitals Bristol and Weston NHS Foundation Trust, Bristol, UK.
4. Bristol Haematology and Oncology Centre, University Hospitals Bristol and Weston NHS Foundation Trust, Bristol, UK.
5. Croydon Sexual Health
6. Southwest Regional Laboratory, UK Health Security Agency, North Bristol NHS Trust, Bristol, UK.
7. National Institute for Health Research, Health Protection Research Unit in Evaluation of Interventions, University of Bristol, Bristol, UK.

Keywords: sexual health; chlamydia; gonorrhoea; rapid STI testing

Corresponding author:
Scott R Walter
National Institute for Health Research
Applied Research Collaboration West (NIHR ARC West)
University Hospitals Bristol and Weston NHS Foundation Trust
9th Floor, Whitefriars, Lewins Mead
Bristol, BS1 2NT, UK
scott.walter@bristol.ac.uk

Word count: 3644

For peer review only - http://bmjopen.bmj.com/site/about/guidelines.xhtml

Abstract

Objectives: To evaluate the impact of a new clinic-based rapid STI testing, diagnosis and treatment service on healthcare delivery and resource needs in an integrated sexual health service.

Design: Controlled interrupted time series study.

Setting: Two integrated sexual health services in UK: Unity Sexual Health in Bristol, UK (intervention site) and Croydon Sexual Health in London (control site).

Participants: Electronic patient records for all 58,418 attendances during the period one year before and one year after the intervention.

Intervention: Introduction of an in-clinic rapid testing system for gonorrhoea and chlamydia in combination with revised treatment pathways.

Outcome measures: Time-to-test notification, staff capacity, cost per episode of care and overall service costs. We also assessed rates of gonorrhoea culture swabs, follow-up attendances, and examinations.

Results: Time-to-notification and the rate of gonorrhoea swabs significantly decreased following implementation of the new system. There was no evidence of change in follow-up visits or examination rates for patients seen in clinic related to the new system. Staff capacity in clinics appeared to be maintained across the study period. Overall, the number of episodes per week was unchanged in the intervention site, and the mean cost per episode decreased by 7.5% (95%CI 5.7%, 9.3%).

Conclusions: The clear improvement in time-to-notification, while maintaining activity at a lower overall cost, suggests that the implementation of clinic-based testing had the intended impact, which bolsters the case for more widespread rollout in SHS.

For peer review only - http://bmjopen.bmj.com/site/about/guidelines.xhtml

Strengths and limitations of this study

- We used controlled interrupted time series models with confounder adjustment to estimate the effect of the intervention distinct from any background changes and independent of other time varying factors.
- Model validity was bolstered by using a relatively long time series with good temporal resolution.
- Data from both the main and control sites was derived from the same electronic patient record system.
- There was a general consensus between main and sensitivity analyses.
- Our study was limited by being non-randomised, having only one control site, and the follow up period for females being truncated by the impact of the Covid-19 pandemic.

For peer review only

1. Introduction

Sexually transmitted infection (STI) diagnoses are increasing in England with more than a 10% increase in new infections between 2016 and 2019[1]. Over the same period, a 19.2% increase in total consultations at sexual health services (SHS) was reported in England[2]. Open-access SHS providing rapid treatment and partner notification can reduce the risk of STI complications and infection spread[3,4,5]. Public Health England (now UK Health Security Agency) recommends that local SHS need to be available to both the general population and groups with greater sexual health needs[3]. Nevertheless, the central government's public health grant, including SHS funding, has steadily decreased since 2015[6,7]. Despite diminishing resources, continued provision of SHS has been achieved through increased efficiencies at clinic-based services and introduction of online services[8,9].

Another approach to improving efficiency while ensuring quality, could be the introduction of near-patient testing (NPT) for chlamydia and gonorrhoea. That is, testing where samples are taken at the time of consultation and results returned within a short timeframe (immediately or within hours). Potential benefits include earlier diagnosis and treatment, reduced risk of sequelae and onward transmission, and reduction in unnecessary treatments, as well as reduced costs and clinician time due to reduction in the need for gonorrhoea cultures, examinations and follow-up visits[10,11,12]. Although modelling studies suggest NPT can be cost-effective, this remains to be demonstrated in practice[10-14]. Research also suggests that reduced waiting times for STI test results may enhance patient acceptability[15,16] and increase testing uptake[17,18]. Importantly, patients have expressed preferences for earlier provision of results[19] due to the stress of waiting[20].

In November 2018, Unity Sexual Health (hereafter *the intervention site*), a UK specialist integrated SHS, implemented a rapid nucleic acid amplification (NAAT) STI testing, diagnosis and treatment service for chlamydia and gonorrhoea, using the Hologic 'Panther' diagnostic platform in a clinic-based satellite laboratory[21]. It can deliver results in 3.5 hours by eliminating sample batching

For peer review only

and transit times associated with microbiology laboratory testing. Integrated sexual health services provide the full range of contraception services in addition to STI and blood borne virus testing, treatment and management and health promotion and prevention.[22]

We used a quantitative approach to evaluate the impact of the new rapid testing process on service delivery and resource needs of the intervention site.

2. Methods

2.1 Setting and design

The intervention site is a provider of integrated SHS in the Bristol area of the United Kingdom, with about 40,000 attendances annually. In addition to in-clinic services, self-testing kits for chlamydia, gonorrhoea, syphilis and HIV ordered online by patients are provided by post. This postal testing kit service was provided by the intervention site for asymptomatic patients through its dedicated website and used the same NAAT testing platform as the rapid STI service. This was in place prior to the intervention and was increasingly used throughout the study period.

This study is a quasi-experimental, controlled interrupted time series (CITS) design that used routinely collected electronic patient record (EPR) data. The intervention time points were defined differently for males and females: rapid STI testing was introduced on 12 November 2018 for males and 29 May 2019 for females.

2.2 Rapid STI service model

Eligibility criteria and treatment pathways differed for males and females. A graphical overview of each pathway is provided in the supplement (Figures S1 and S2) with pre-intervention pathway included for reference. Additional changes were made to the SHS related to staff capacity. Rapid STI asymptomatic consultations were reduced to 15 minutes, while the number of allocated patients per staff member for the walk-in clinic remained the same.

For peer review only - http://bmjopen.bmj.com/site/about/guidelines.xhtml

2.2.1 Males

Male patients were eligible for the rapid STI pathway if they were asymptomatic or had urethritis symptoms. If asymptomatic, a brief history was taken prior to patient self-sampling for chlamydia and gonorrhoea and taking blood tests for HIV and syphilis. Men who have sex with men (MSM) were referred to a health adviser for health promotion, including discussion about testing for HIV and other STIs, and safer sex practices. Symptomatic men were asked to return four hours later when NAAT results were available. If positive, they received infection specific treatment; if negative a urethral smear was undertaken to diagnose non-gonococcal urethritis. Contacts of patients with gonorrhoea or chlamydia outside a two-week window were treated if NAAT-positive. Swabs for gonococcal culture and sensitivities were only taken after a NAAT-positive result for gonorrhoea or if gonococcal treatment was administered prior to the NAAT result.

2.2.2 Females

Female asymptomatic patients without contraception needs were eligible for the rapid drop-off service. Women with abnormal vaginal discharge, not requiring bimanual or speculum examination to exclude pathology, self-swabbed and were treated on the results of microscopy and clinical findings at the time of visit and informed that chlamydia and gonorrhoea NAAT test results would be available within 48 hours. They were termed symptomatic. For contraceptive needs, a clinical consultation was necessary to determine the need for examination. Trichomonas vaginalis (TV) culture was replaced with a more sensitive TV NAAT[23], also available within 48 hours. A gonococcal culture swab was only taken after a NAAT-positive result for gonorrhoea or if gonococcal treatment was administered prior to NAAT result.

2.3 Control site

For peer review only - http://bmjopen.bmj.com/site/about/guidelines.xhtml

Croydon Sexual Health, a similar integrated SHS in South London, was used as the control site to account for background changes unrelated to the intervention. This site has similar patient throughput (about 32,000 annual attendances) and uses the same EPR system.

2.4 Data

Fully anonymised individual patient data extracted from the intervention and control site EPR systems[23] comprised demographic information, sexual behaviour, mode of presentation and attendances to the clinic, diagnostic testing and treatment. Analyses were based on a census of attendance level records.

Time-to-notification was defined from the text message notification system[24]. This included text message type for identifying test results messages, time stamps and anonymised patient identifiers. Numbers of NAAT postal testing kits were extracted from the intervention site's records, while the control site did not implement these until after the study period.

Prior to analysis, data were checked for duplicates, implausible values and missingness. Individual variables were combined to generate indicator variables for complex cases, MSM, examinations, ethnic minority status. All time-related variables were derived from the date and time of each attendance.

For analysis, data were aggregated at weekly level over a two-year period centred at the intervention. For females, data were excluded from the first UK Covid-19-related lockdown (23 March 2020) due to changes in outcomes that could not be adequately accounted for in models. The study period for males was from 13 November 2017 to 10 November 2019, and for females 28 May 2018 to 22 March 2020.

2.5 Statistical analysis

There main study outcomes are detailed in Table 1. CITS models within a generalised linear modelling framework were applied to each outcome separately for males and females: ten models

For peer review only - http://bmjopen.bmj.com/site/about/guidelines.xhtml

in total. $Time$ was modelled as linear using consecutively numbered weeks, with $time = 0$ at the intervention point. A binary variable ($period$) representing pre- and post-intervention periods was defined by the respective male and female intervention dates.

Gonorrhoea culture swabs per consultation, follow-up attendances per care episode, examinations per symptomatic attendance and staff capacity were modelled as rates assuming a negative binomial distribution. These models generate rate ratios, presented as percentage changes. For time-to-notification, a normal distribution was assumed and results presented as differences in median time (days). This represents absolute measure of time including weekends as opposed to working days only.

Table 1. Definitions of main study outcomes.

Outcome measure	Definition
1. Rate of gonorrhoea culture swabs per consultation	Numerator: the number of GC swabs, urethral for male and cervical for female<br>Denominator: the number of consultations where these were defined as attendances for new, rebooked or walk-in patients
2. Time-to-notification	Median time from sample collection until the patient was notified of the test result via text message
3. Rate of examinations per symptomatic attendance	Numerator: the number of examinations of any type. This was based on a combination of variables used to record information about examinations (supplementary Table S1)<br>Denominator: all attendances where the patient was recorded as being symptomatic
4. Rate of follow up attendances per episode of care	Numerator: the number of follow up attendances occurring within 30 days of an initial consultation<br>Denominator: the number of episodes involving at least 1 consultation
5. Staff capacity – rate of patients seen per four-hour clinic	Numerator: number of patient consultations (any new, rebooked, walk-in or follow up attendance)<br>Denominator: number staff available for four-hour clinics

The main variables in the models were $time$, $period$ and $site$ (intervention vs. control) along with all two-way and three-way interactions, as per a CITS approach for estimating both a step change and slope change[25,26]. Two key terms in the models represent intervention-related changes over and above any control site changes. The interaction $period \times site$ captures a

For peer review only - http://bmjopen.bmj.com/site/about/guidelines.xhtml

differential step change for the intervention site compared to control site. While the three-way interaction term $time \times period \times site$ captures different degrees of pre-post trend change for the intervention site compared to control site (supplement Figure S3).

Additional covariates were included in the models: proportions of complex patients, symptomatic patients and patients from an ethnic minority, plus mean patient age and calendar month. Since models of examination rate only analysed symptomatic patients, the proportion of symptomatic patients was excluded as a covariate. The proportion of MSM was only included in models for males. Complex cases were defined differently for males and females (definition S1). This is based on the definition used by Mohiuddin et al.[12] designed to identify patients requiring longer and/or more involved consultations.

Data for staff capacity was only available for the intervention site and was modelled as an uncontrolled interrupted time series spanning the duration of available denominator data: 1 January 2018 to 22 December 2019. The denominator could not be separated by gender, so this outcome was analysed for females and males combined, allowing two change points as per the respective intervention dates.

Sensitivity analyses were conducted by fitting generalised additive models to account for potential non-linearity of trends. All analyses were conducted with the SAS System for Windows, version 9.4 (SAS Institute Inc.). Models were fitted using the GENMOD and GAM procedures.

2.6 Economic analysis

Postal testing kit data were combined with EPR data to estimate the total number of episodes per week (including those with negative postal tests and no clinic attendance). For estimating the difference in the mean number of episodes per week i) negative postal test episodes were assigned to weeks pro rata with asymptomatic episodes that included clinic attendance, and ii) the combined post-intervention analysis used data for the first 43 weeks only. Episode costs were estimated using unit costs of diagnostic tests provided by the intervention site, and postal kit tests and staff time

For peer review only - http://bmjopen.bmj.com/site/about/guidelines.xhtml

from the literature[12] inflated to 2021 values using a UK government GDP deflator[27]. Treatment costs were from the British National Formulary[28] (supplement Table S2). The cost of unreturned postal kits was allocated to episodes including a postal test result. Confidence intervals for differences in the number of episodes and cost per episode were calculated using the Normal approximation method.

Patient and public involvement

Three members of the public who had used the intervention site services as patients were involved in reviewing the proposed outcome measures and informed the study design.

3. Results

In the EHR intervention site data, 48,776 attendances for females and 34,413 for males were recorded during the study period, representing 32,482 and 22,073 episodes of care involving a clinic attendance, and 29,573 and 19,083 patients, respectively (Table 2). Patients were symptomatic in just over 20% of female attendances, and over 40% of male attendances. About 90% of female and 55% of male attendances were complex. Just over 30% of male attendances were by MSM.

3.1 Males

There was strong evidence of an adjusted step-increase for the intervention site relative to the control site (+89.1%, 95% confidence interval [CI] +37.1%, +160.6%, p<0.001) (Table 3 and Figure 1A). However, this was not observed in the sensitivity analysis allowing for non-linear trends (-16.6%, 95%CI -30.1%, -0.5%, p<0.001, supplement Table S3 and Figure S4A). This was followed by strong evidence of an adjusted downward change in post-intervention trend of -3.2% per week (95% CI -4.3%, -2.1%, p<0.001). The long-term result of these two effects was an overall decrease from 35-

For peer review only - http://bmjopen.bmj.com/site/about/guidelines.xhtml

For peer review only

50 swabs per week, pre-intervention, to below 10 at the end of the study period, translating to 849 swabs avoided over the post-intervention period.

Time-to-notification increased by an estimated 3.6 days (95% CI 1.7, 5.5 days, $p<0.001$) at the time of the intervention, relative to controls, and a similar increase was observed in the sensitivity analysis. However, this was followed by an overall long-term decrease of -0.2 days of notification time per week (95% CI -0.3, -0.2 days, $p<0.001$) through the post-intervention period. That is, the pre-intervention weekly median of around eight to nine days dropped to around 2 days after the intervention had been in place for a year (Figure 1B, supplement Figure S4B).

We found no evidence of a meaningful change in rates of examinations or follow-up attendances associated with the intervention (Table 3, Figures 1C and 1D, supplement Figures S4C and S4D).

For peer review only

For peer review only - http://bmjopen.bmj.com/site/about/guidelines.xhtml

Table 2. Summary of population characteristics and outcomes by site, gender and time period based on EPR data.

	**Intervention site**		**Control site**	
	**Pre**	**Post**	**Pre**	**Post**
**MALES**				
Total attendances, n	17626	16787	11920	12085
Total episodes of care, n	11445	10628	7946	8021
Total patients, n	9932	9151	6271	6335
Symptomatic attendances, n (%)	7307 (41.5%)	7084 (42.2%)	4735 (39.7%)	4556 (37.7%)
Complex attendances, n (%)	9869 (56.0%)	9259 (55.2)%	4458 (37.4%)	4940 (40.9%)
Ethnic minority attendances, n (%)	2834 (16.1%)	3025 (18.0%)	7244 (60.8%)	7311 (60.5%)
MSM attendances, n(%)	5300 (30.1%)	5418 (32.3%)	2529 (21.2%)	2849 (23.6%)
Mean age, years	30.2	30.8	34.9	35.1
Urethral GC swabs per consultation	0.18	0.11	0.08	0.07
Median time-to-notification	10.90	6.73	4.51	4.95
Examinations per symptomatic attendance	0.76	0.67	0.64	0.60
Follow up attendances per episode	0.40	0.36	0.50	0.37
**FEMALES**				
Total attendances	28487	20289	20931	16910
Total episodes of care	18616	13866	13971	11660
Total patients	16779	12794	11799	9902
Symptomatic attendances	6312 (22.2%)	4929 (24.3%)	6860 (32.8%)	5561 (32.9%)
Complex attendances	26022 (91.3%)	18173 (89.6%)	12328 (58.9%)	11221 (66.4%)
Ethnic minority attendances	3979 (14.0%)	3067 (15.1%)	12647 (60.4%)	10107 (59.8%)
Mean age	25.1	25.8	29.8	30.4
Cervical GC swabs per consultation	0.20	0.04	0.03	0.03
Median time-to-notification (median, IQR)	10.58	3.52	4.90	5.32
Examinations per symptomatic attendance	0.73	0.70	0.58	0.60
Follow up attendances per episode	0.36	0.34	0.31	0.23

For peer review only - http://bmjopen.bmj.com/site/about/guidelines.xhtml

Table 3. Step change and slope change estimates from controlled interrupted time series models by outcome and sex. Change estimates are shown for the intervention and control sites and for the relative change for intervention site compared to the control site. All estimates are shown as percentage changes, except for time-to-notification where change estimates are given in days.

Outcome	Change at time of intervention			Trend change following intervention		
	Intervention site	*Control site*	*Intervention vs. control*	*Intervention site*	*Control site*	*Intervention vs. control*
**MALES – 12th November 2018**						
**1. Gonorrhoea culture swabs per consultation**	+6.5%	-43.7%	**+89.1% (+37.1%, +160.9%)**	-3.6%	-0.3%	**-3.2% (-4.3%, -2.1%)**
**2. Time-to-notification**	+2.2 days	+5.8 days	**+3.6 days (+1.7, +5.5)**	-0.19 days	+0.03 days	**-0.2 days (-0.3, -0.2)**
**3. Examinations per symptomatic attendance**	+3.6%	-1.6%	+5.4% (-7.5%, +20.0%)	-0.21%	-0.16%	-0.04% (-0.5%, +0.4%)
**4. Follow up attendances per episode**	-9.0%	-11.9%	+3.3% (-14.6%, +24.9%)	+0.23%	-0.001%	+0.30% (+0.31%, +0.96%)
**FEMALES – 29th May 2019**						
**1. Gonorrhoea culture swabs per consultation**	-38.7%	+3.6%	**-40.8% (-61.6%, -8.8%)**	-6.1%	-0.1%	**-6.1% (-7.8%, -4.5%)**
**2. Time-to-notification**	-2.5 days	-0.4 days	-2.1 (-4.5, 0.3) days	-0.11 days	-0.0001 days	**-0.1 (-0.2, -0.0) days**
**3. Examinations per symptomatic attendance**	-1.3%	-2.2%	+1.0% (-11.4%, +15.1%)	+0.09%	+0.03%	+0.1% (-0.4%, +0.5%)
**4. Follow up attendances per episode**	-8.2%	+2.7%	-10.6% (-27.6%, +10.3%)	-0.42%	+0.22%	-0.64% (-1.41%, +0.14%)

Note: Results for outcome 5 (staff capacity) reported separately in the text.

For peer review only - http://bmjopen.bmj.com/site/about/guidelines.xhtml

3.2 Females

For females, there was evidence of a decrease in the rate of gonorrhoea culture (GC) swabs: -40.8% (95% CI -61.6%, -8.8%, p=0.02) at the time of intervention, adjusted for control changes (Table 3, Figure 2A). This was followed by a decrease in trend through the post-intervention period, with an adjusted change of -6.1% per week (95% CI -7.8%, -4.5%, p<0.001). These changes represent a decrease from an estimated 0.22 swabs per consultation (over 30 swabs per week) immediately before the intervention to 0.14 immediately after (20 to 25 per week) and down to 0.01 at the end of the study period (less than five per week). Over the 43-week post-intervention period, an estimated 1542 swabs were avoided.

For time-to-notification, there was some evidence of a decrease of 2.1 days (95% CI -4.5, 0.3 days, p=0.08, Figure 2B) at the time of the intervention, adjusted for the control group. There was stronger evidence of a downward change in trend, estimated at -0.1 days per week (95% CI -0.20, -0.0 days, p=0.01) over the post-intervention period. These results were confirmed by the sensitivity analyses (Figure S5). To illustrate, the estimated median time-to-notification was eight to nine days just before the intervention, but a year later notification time was around one day.

For rates of examinations and follow up visits, we saw no evidence of intervention-related change (Table 3, Figures 2C and 2D).

[Figures 1 and 2 about here]

3.3 Staff capacity

The main analysis of staff capacity showed evidence of a trend change at the time of the male intervention (-1.1% per week, 95%CI -1.7%, -0.5%, p<0.001) and a step change at the time of the female intervention (+14.3%, 95% CI +3.4%, +26.3%, p=0.009) (Figure 3). However, the sensitivity analysis showed step changes in the opposite direction to the main analysis (supplement Figure S6), suggesting inconclusive evidence of change.

[Figure 3 about here]

3.4 Episodes and costs

For peer review only - http://bmjopen.bmj.com/site/about/guidelines.xhtml

Overall, the intervention site experienced a substantial increase in the weekly number of asymptomatic negative episodes managed via postal test kits, particularly for males, while both asymptomatic negative episodes seen in the clinic and symptomatic episodes decreased (Table 4). The mean cost per symptomatic episode increased by 9.2% to £69.04, while this was outweighed by a decrease of 13.5% to £26.23 for costs per asymptomatic episode, resulting in a combined decrease of 7.5%. The total cost per week decreased by 4.7%, largely due to the reduction in both the number and cost of episodes for asymptomatic females who attended the clinic.

For peer review only

For peer review only - http://bmjopen.bmj.com/site/about/guidelines.xhtml

Table 4. Intervention site pre- and post-intervention estimates of mean number of episodes per week, mean cost per episode and mean cost per week.

	Male					Female					Total				
	pre*	post*	% change	95% CI		pre*	post**	% change	95% CI		pre*	post**	% change	95% CI	
Mean number per week															
Asymptomatic	190.2	223.1	17.3	9.5	25.1	356.2	350.7	-1.5	-7.9	4.9	546.3	573.4	5.0	0.0	9.9
Postal negatives	70.5	111.5	58.2	48.7	67.7	96.3	124.9	29.7	22.3	37.0	166.8	236.2	41.6	35.7	47.4
Other^	119.6	111.5	-6.8	-13.7	0.2	259.9	225.9	-13.1	-19.2	-7.0	379.5	337.2	-11.1	-15.8	-6.5
Symptomatic	92.7	85.0	-8.3	-13.9	-2.7	84.4	77.8	-7.8	-14.6	-1.1	176.7	163.4	-7.5	-11.8	-3.2
Total	282.8	308.0	8.9	2.6	15.2	440.2	429.0	-2.5	-8.7	3.6	723.0	736.8	1.9	-2.5	6.3
Cost per episode (£)															
Asymptomatic	36.47	30.92	-15.2	-19.1	-11.3	27.04	24.23	-10.4	-13.3	-7.5	30.31	26.23	-13.5	-15.9	-11.0
Symptomatic	63.09	69.56	10.3	6.7	13.8	63.36	67.65	6.8	4.3	9.2	63.22	69.04	9.2	6.9	11.5
Total	45.19	41.58	-8.0	-10.8	-5.2	33.98	32.14	-5.4	-7.7	-3.1	38.36	35.47	-7.5	-9.3	-5.7
Cost per week (£)															
Resource															
Postal kit	382	592	55.0	45.9	64.1	629	848	34.8	27.4	42.2	1010	1437	42.3	36.5	48.1
In clinic diagnostic test	1962	1886	-3.9	-9.8	2.1	1452	1213	-16.5	-22.9	-10.1	3413	3155	-7.6	-11.9	-3.3
Consultation staff time	7497	7349	-2.0	-7.3	3.4	9396	8583	-8.7	-15.0	-2.3	16893	15959	-5.5	-9.5	-1.5
Treatment	3024	2896	-4.2	-13.1	4.6	3534	3085	-12.7	-20.3	-5.1	6558	6014	-8.3	-14.4	-2.2
Symptom status															
Asymptomatic	6949	6883	-1.0	-8.5	6.6	9673	8448	-12.7	-18.9	-6.4	16622	15392	-7.4	-12.3	-2.5
Symptomatic	5915	5840	-1.3	-7.5	5.0	5338	5280	-1.1	-8.7	6.6	11253	11174	-0.7	-5.5	4.0
Total	12865	12723	-1.1	-6.7	4.5	15010	13728	-8.5	-14.4	-2.6	27875	26565	-4.7	-8.6	-0.8

* based on 52 week period.
** based on 43 week period
^ includes positive postal test kits

For peer review only

For peer review only - http://bmjopen.bmj.com/site/about/guidelines.xhtml

4. Discussion

We have quantitatively evaluated the impact of a first-of-its-kind rapid STI testing on service delivery in an integrated SHS. Previous NPT assessments have taken a mathematical modelling approach[11-13]. The only other direct assessment of a chlamydia and gonorrhoea NPT in practice related to a rapid testing service model for asymptomatic patients without contraception provision[29]. This is the first study to quantify the effect of rapid chlamydia and gonorrhoea NPT on gonorrhoea culture swabs, time-to-notification, examinations, follow-up visits, staff capacity, and costs.

The substantial long term post-intervention decrease in the rate at which gonorrhoea swabs were sent for culture, for both males and females, was expected to some extent since patients with negative rapid tests in the new pathway avoided the need for cultures. Adams et al. [11] identified reduced gonorrhoea cultures as a key part of NPT-related cost reduction, although there has been no direct or simulated assessment of expected change in the number of cultures.

The trajectory of the decline in gonorrhoea swab rates following the intervention differed between males and females. The sensitivity analysis capturing non-linear trends suggested substantial decreases for males began more than six months after the intervention, with the lowest rates at one year post-intervention (Figure S4A). In contrast, rates for females appeared to respond to the intervention almost immediately and stabilise at a much lower level within about six months (Figure S5A). The differing implementation timeframes may reflect several barriers to implementation with the initial rollout for males, including providing training to a large group staff with varying timetables exacerbated by understaffing and budget cuts; variable application of eligibility criteria for the new service; and iterative revision of the new system and pathway[30]. There may also have been some just-in-case culture testing in the early stages until staff confidence in the system was established. With these issues largely resolved when the system was implemented for females, the transition appeared both smoother and faster, and this concurs with staff experience.

For peer review only - http://bmjopen.bmj.com/site/about/guidelines.xhtml

The rate of gonorrhoea swabs at the control site was relatively low throughout the period due to a conservative approach, appropriate to local prevalence, in which samples for cultures were only taken for NAAT-positive patients or those with high likelihood of infection. In contrast, standard practice at the intervention site in the pre-intervention period was to take cultures from all symptomatic patients with symptoms and/or signs potentially consistent with gonorrhoea and from potential contacts in addition to a NAAT as recommended in national guidelines.[31]

We estimated that median time-to-notification decreased from more than a week down to one or two days over the post-intervention period. However, given that it was not possible to separate out all rapid test results (e.g. notifications labelled "all negative") and that we estimated real time rather than working days, the median time was likely lower, particularly for positive results. This is broadly consistent with findings from Whitlock et al. [29] who reported an average time-to-notification of 0.27 days for a new rapid NAAT testing service compared to 8.95 days for an off-site testing service for symptomatic patients.

The temporary increase in median time-to-notification for males after the intervention may result from the implementation challenges outlined above[30] in addition to a clinician-reported backlog in the early stages of transitioning to the new system. Once again, for males the transition appeared to take place over the full post-intervention period, while the equivalent period for females appeared faster with the lowest post-intervention sensitivity estimates occurring 21 weeks after the new system was implemented (supplement Figures S4B and S5B).

We observed no clear evidence of intervention-related changes in rates of examinations, follow up visits or staff capacity. All three were necessarily constructed from combinations of variables as there was no dedicated data field for each in the data. Although we did not detect a positive change, it is important to note that there was no evidence of a deleterious impact of the rapid testing service on any of these outcomes.

Staff capacity showed some evidence of intervention-related change, although the rate of patients seen per four-hour clinic was at similar levels at the end of the study period as at the start.

For peer review only - http://bmjopen.bmj.com/site/about/guidelines.xhtml

For asymptomatic patients, the provision of postal testing kits reduced the need for clinic attendance among those testing negative both for males and for females who did not have contraception needs. This combined with the introduction of shorter appointments more than likely increased staff capacity for this subgroup. Both also reduced the queueing time for walk-in clinics. Conversely, the reduced asymptomatic attendances meant that case-mix in the walk-in clinics became more demanding, with patients more likely to be symptomatic and/or complex[30], which may explain the lack of observed improvement in staff capacity during clinics. The lack of evidence for a capacity decrease through the implementation period despite a more demanding patient group and the growing numbers of asymptomatic patients being tested both suggest increased capacity of the SHS overall.

The change in management of asymptomatic clinical attendances, supported by the existing postal testing kit system, was a key component of the overall cost reduction following the introduction of the Panther technology, with decreases in both mean cost per asymptomatic episode (13.5%) and weekly asymptomatic costs (7.4%). Although the cost of symptomatic episodes increased, consistent with the reported increase in complexity of symptomatic patients in clinic, this was counteracted by a reduction in the number of weekly symptomatic attendances.

4.1 Strengths and limitations

We conducted a prospective real-time evaluation of a large integrated rapid STI service. We used a CITS framework with both a control site and confounder adjustment to estimate the effect of the intervention distinct from any background changes and independent of other time varying factors. This was bolstered by using a relatively long time series with good temporal resolution. The robustness of our analysis was supported by both sites using the same EPR system and the general consensus between main and sensitivity analyses.

In light of the target trial framework for natural experiments[32], our study was limited by being non-randomised, having only one control site, relying on the construction of certain outcomes

For peer review only - http://bmjopen.bmj.com/site/about/guidelines.xhtml

from multiple variables, and the impact of the Covid-19 pandemic on the follow up period for females. The unit costs were based on data provided by the intervention site and estimates from literature, and commissioners will need to assess their applicability to their locality.

4.2 Implications and conclusions

Several studies have suggested that NPT benefits include earlier diagnosis and treatment, reduced risk of sequelae and onward transmission, reduction in unnecessary treatments, earlier partner notification and reduced anxiety [10,29].

This quantitative assessment of the first UK implementation of rapid chlamydia and gonorrhoea testing within an integrated service revealed clear benefits, namely: reduced gonorrhoea culture swabs and shortened time-to-notification. These improvements, while maintaining activity at a lower overall cost, suggests that the introduction of clinic-based rapid testing had the intended impact, and this is in line with previous NPT modelling studies [10,11]. The qualitative evaluation of this rapid STI service also reported that patients valued faster results and avoiding unnecessary treatment, and that the better targeting of infection-specific treatment improved antimicrobial stewardship[30]. Although this was an evaluation of an integrated SHS providing contraception care in addition to testing, treatment and prevention services, it is likely the findings would be applicable to sexual health services which do not provide contraception care.

These results provide real-life evidence to support the benefits of a rapid testing service anticipated by modelling studies and strengthen the case for more widespread rollout in SHS.

For peer review only - http://bmjopen.bmj.com/site/about/guidelines.xhtml

Acknowledgements: The authors would like to thank Ed Hulse at Mill Systems for his indispensable assistance with the data extracts.

Funding statement: This research was funded by the National Institute for Health Research (NIHR) Applied Research Collaboration West (ARC West) at University Hospitals Bristol and Weston NHS Foundation Trust (core NIHR infrastructure funded: NIHR200181). The views expressed are those of the authors and not necessarily those of the NIHR or the Department of Health and Social Care. FdV is partly funded by the NIHR School for Public Health Research.

Competing interests: There are no competing interests to declare for any of the authors.

Data sharing statement: Anonymised individual-level data for this study comes from the electronic patient record system of the Unity Sexual Health and Croydon Sexual Health services (data controllers). Our data sharing agreement with the data controllers prohibits sharing data extracts outside of the University of Bristol research team. The data is available upon request from the data controllers.

Licence statement: I, the Submitting Author has the right to grant and does grant on behalf of all authors of the Work (as defined in the below author licence), an exclusive licence and/or a non-exclusive licence for contributions from authors who are: i) UK Crown employees; ii) where BMJ has agreed a CC-BY licence shall apply, and/or iii) in accordance with the terms applicable for US Federal Government officers or employees acting as part of their official duties; on a worldwide, perpetual, irrevocable, royalty-free basis to BMJ Publishing Group Ltd ("BMJ") its licensees and where the relevant Journal is co-owned by BMJ to the co-owners of the Journal, to publish the Work in BMJ Open and any other BMJ products and to exploit all rights, as set out in our licence.

The Submitting Author accepts and understands that any supply made under these terms is made by BMJ to the Submitting Author unless you are acting as an employee on behalf of your employer or a postgraduate student of an affiliated institution which is paying any applicable article publishing charge ("APC") for Open Access articles. Where the Submitting Author wishes to make the Work available on an Open Access basis (and intends to pay the relevant APC), the terms of reuse of such Open Access shall be governed by a Creative Commons licence – details of these licences and which Creative Commons licence will apply to this Work are set out in our licence referred to above.

Ethics approval: This study was approved by the Health Research Authority (South West) Research Ethics Committee, reference 18/SW/0090.

Contributorship statement: PH, MTR, FdV and HM conceptualized the evaluation; MTR and FdV are quantitative evaluation leads; WH and HM are health economic evaluation leads; SRW, JJ, RM and MTR acquired the analysis datasets; SRW conducted the time series analysis with supoprt from JJ, RM, MTR, PH and FdV; GM conducted the cost-effectiveness analysis with support from HM and WH; RG, MDC, MC, DP, PM, JS and JT advised on the study methodology, analysis and interpretation of results; SRW wrote the initial draft of the manuscript; all authors reviewed and edited the manuscript for content and approved the submission.

For peer review only

References

1. Public Health England, National STI surveillance data tables 2020 - Table 4. Available at: https://www.gov.uk/government/statistics/sexually-transmitted-infections-stis-annual-data-tables

2. Public Health England, National STI surveillance data tables 2020 - Table 3. Available at: https://www.gov.uk/government/statistics/sexually-transmitted-infections-stis-annual-data-tables

3. Mitchell H, Allen H, Sonubi T, Kuyumdzhieva G, Harb A, Shah A, Glancy M, Checchi M, Milbourn H, Folkard K, Mohammed H and contributors. Sexually transmitted infections and screening for chlamydia in England, 2019. September **2020**. London: Public Health England. Available at: https://assets.publishing.service.gov.uk/government/uploads/system/uploads/attachment_data/file/914249/STI_NCSP_report_2019.pdf

4. Price MJ, Ades AE, Soldan K, Welton NJ, Macleod J, Simms I, DeAngelis D, Turner KM, Horner PJ. The natural history of Chlamydia trachomatis infection in women: a multi-parameter evidence synthesis. Health Technol Assess, **2016**; 20: 1-250.

5. Ratna N, Sonubi T, Glancy M, Sun S, Harb A, Checchi M, Milbourn H, Dunn J, Sinka K, Folkard K, Mohammed H and contributors. Sexually transmitted infections and screening for chlamydia in England, 2020. September **2021**. London: Public Health England. Available at: https://assets.publishing.service.gov.uk/government/uploads/system/uploads/attachment_data/file/1015176/STI_NCSP_report_2020.pdf

6. Finch D, Bibby J, Elwell-Sutton T. Briefing: Taking our Health for Granted. October **2018**. London: The Health Foundation. Available online: https://www.health.org.uk/publications/taking-our-health-for-granted

7. White C. Sexual health services on the brink. Brit Med J, **2017**; 359: j5395.

8. Turner KME, Zienkiewicz AK, Syred J, Looker KJ, de Sa J, Brady M, Free C, Holdsworth G, Baraitser P. Web-Based Activity Within a Sexual Health Economy: Observational Study. J Med Internet Res, **2018**; 20:e74.

9. Turner KME, Looker KJ, Syred J, Zienkiewicz A, Baraitser P. Online testing for sexually transmitted infections: A whole systems approach to predicting value. PLoS ONE, **2019**; 14: e0212420. doi: 10.1371/journal.pone.0212420

10. Turner KME, Round J, Horner PJ, Macleod J, Goldenberg S, Deol A, Adams EJ. An early evaluation of clinical and economic costs and benefits of implementing point of care NAAT tests for Chlamydia trachomatis and Neisseria gonorrhoea in genitourinary medicine clinics in England. Sex Transm Infect, **2014**; 90: 104–111. doi: 10.1136/sextrans-2013-051147

11. Adams EJ, Ehrlich A, Turner KME, Shah K, Macleod J, Goldenberg S, Meray RK, Pearce V, Horner PJ. Mapping patient pathways and estimating resource use for point of care versus standard testing and treatment of chlamydia and gonorrhoea in genitourinary medicine clinics in the UK. BMJ Open, **2014**; 4: e005322. doi: 10.1136/bmjopen-2014-005322

12. Mohiuddin S, Gardiner R, Crofts M, Muir P, Steer J, Turner J, Wheeler H, Hollingworth P, Horner PJ. Modelling patient flows and resource use within a sexual health clinic through discrete event

For peer review only - http://bmjopen.bmj.com/site/about/guidelines.xhtml

simulation to inform service redesign. BMJ Open, **2020**;10: e037084. doi: 10.1136/bmjopen-2020-037084

13. Huntington SE, Burns RM, Harding-Esch E, Harvey MJ, Hill-Tout R, Fuller SS, Adams EJ, Sadiq ST. Modelling based evaluation of the costs, benefits and cost-effectiveness of multipathogen point-of-care tests for sexually transmitted infections in symptomatic genitourinary medicine clinic attendees. BMJ Open, **2018**; 8: e020394. doi: 10.1136/bmjopen-2017-020394

14. Miners A. Is nucleic acid amplification point-of-care testing for chlamydia and gonorrhoea cost-effective? Sex Transm Infect, **2014**; 90: 82.

15. Natoli L, Guy RJ, Shephard M, Causer L, Badman SG, Hengel B, Tangey A, Ward J, Coburn T, Anderson D, Kaldor J, Maher L. "I Do Feel Like a Scientist at Times": A Qualitative Study of the Acceptability of Molecular Point-Of-Care Testing for Chlamydia and Gonorrhoea to Primary Care Professionals in a Remote High STI Burden Setting. PloS ONE, **2016**; 10: e0145993. doi: 10.1371/journal.pone.0145993

16. Rompalo AM, Yu-Hsiang H, Hogan T, Barnes M, Jett-Goheen M, Huppert JS, Gaydon CA. Point-of-care tests for sexually transmissible infections: what do 'end users' want? Sex Health, **2013**; 10: 541-545. doi: 10.1071/SH13047

17. Horwood J, Ingle SM, Burton D, Woodman-Bailey A, Horner PJ, Jeal N. Sexual health risks, service use, and views of rapid point-of-care testing among men who have sex with men attending saunas: a cross-sectional survey. Int J STD AIDS, **2016**; 27: 273-280. doi: 10.1177/0956462415580504

18. Lorenc T, Marrero-Guillamón I, Aggleton P, Cooper C, Llewellyn A, Lehmann A, Lindsay C. Promoting the uptake of HIV testing among men who have sex with men: systematic review of effectiveness and cost-effectiveness. Sex Transm Infect, **2011**; 87: 272-278. doi: 10.1136/sti.2010.048280

19. Llewellyn CD, Sakal C, Lagarde M, Pollard A, Miners AH. Testing for sexually transmitted infections among students: a discrete choice experiment of service preferences. BMJ Open, **2013**; 3. doi: 10.1136/bmjopen-2013-003240

20. Llewellyn C, Pollard A, Miners A, Richardson D, Fisher M, Cairns J, Smith H. Understanding patient choices for attending sexually transmitted infection testing services: a qualitative study. Sex Transm Infect, **2012**; 88: 504-509. doi: 10.1136/sextrans-2011-050344

21. Hologic Inc. Panther® System. https://www.hologic.com/hologic-products/diagnostic-solutions/panther-scalable-solutions/panther-system

22. Department of Health and Social Care, Public Health England. Integrated Sexual Health Services: A suggested national service specification. August 2018: London. Available at: https://assets.publishing.service.gov.uk/government/uploads/system/uploads/attachment_data/file/731140/integrated-sexual-health-services-specification.pdf

23. Nicholls JE, Turner KME, North P, Ferguson R, May MT, Gough K, Macleod J, Muir P, Horner PJ. Cross-sectional study to evaluate Trichomonas vaginalis positivity in women tested for Neisseria gonorrhoeae and Chlamydia trachomatis, attending genitourinary medicine and primary care clinics in Bristol, South West England. Sex Transm Infect. **2018**;94: 93-99. doi: 10.1136/sextrans-2016-052942.

For peer review only - http://bmjopen.bmj.com/site/about/guidelines.xhtml

24. Mill Systems Limited. https://www.millsystems.com/millcare/

25. Simonton DK. Cross-sectional time series experiments: Some suggested statistical analyses. Psychol Bull, **1977**; 84: 489-502.

26. Bernal JL, Cummins S, Gasparrini A. The use of controls in interrupted time series studies of public health interventions. Int J Epi, **2018**; 47: 2082–2093. doi: 10.1093/ije/dyy135.

27. HM Treasury. GDP deflators at market prices, and money GDP December 2021 (Quarterly National Accounts). Available at: https://www.gov.uk/government/statistics/gdp-deflators-at-market-prices-and-money-gdp-december-2021-quarterly-national-accounts

28. Joint Formulary Committee. British National Formulary. **2020**. Available at: http://www.medicinescomplete.com

29. Whitlock GG, Gibbons DC, Longford N, Harvey MJ, McOwan A, Adams EJ. Rapid testing and treatment for sexually transmitted infections improve patient care and yield public health benefits. Int J STD AIDS, **2018**; 29: 474–482.

30. Lorenc A, Kesten J, Brangan E, Horner PJ, Clarke M, Crofts M, Turner J, Muir P, Horwood J. What can be learnt from a qualitative evaluation of implementing a rapid sexual health testing, diagnosis and treatment service? BMJ Open, **2021**; 11: e050109. doi: 10.1136/bmjopen-2021-050109

31. Fifer H, Saunders J, Soni S, Tariq Sadiq S, FitzGerald M. 2018 UK national guideline for the management of infection with *Neisseria gonorrhoeae*. Int J STD AIDS, **2019**; 31: 4-15.

32. de Vocht F, Katikireddi SV, McQuire C, Tilling K, Hickman M, Craig P. Conceptualising natural and quasi experiments in public health. BMC Med Res Methodol, **2021**; 21: 32. doi:10.1186/s12874-021-01224-x

For peer review only

For peer review only - http://bmjopen.bmj.com/site/about/guidelines.xhtml

Figure legends

Figure 1. Modelled outcome estimates for males. 'Panther' indicates the intervention-date representing the first week the Panther system was implemented for the male pathway: 12 November 2018.

Figure 2. Modelled outcome estimates for females. 'Panther for females' indicates the intervention-date representing the first week the Panther system was implemented for the female pathway: 29 May 2019.

Figure 3. Modelled estimates of staff capacity for males and females combined.

For peer review only

For peer review only - http://bmjopen.bmj.com/site/about/guidelines.xhtml

Figure 1. Modelled outcome estimates for males. 'Panther' indicates the intervention-date representing the first week the Panther system was implemented for the male pathway: 12 November 2018.

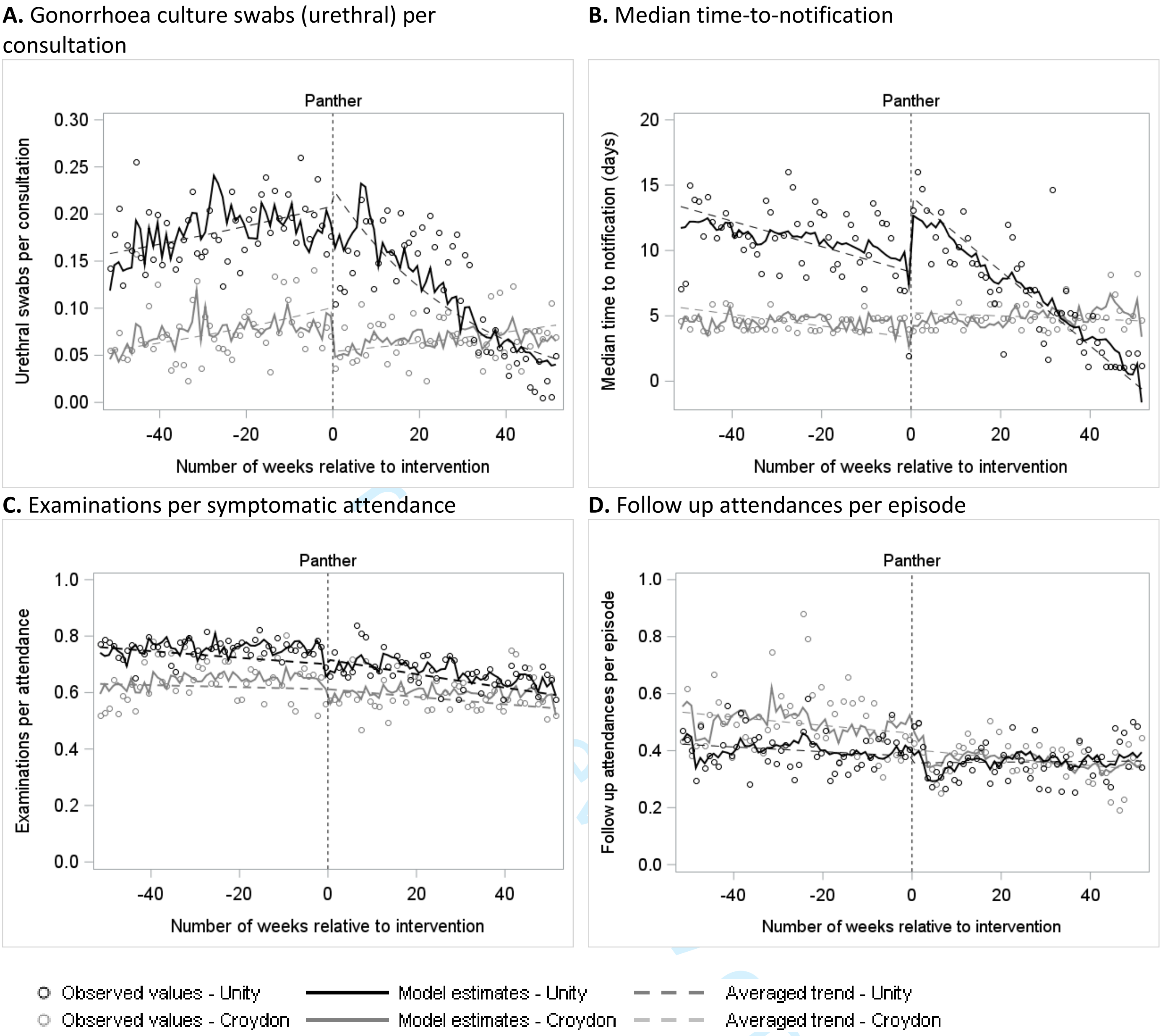

Figure 2. Modelled outcome estimates for females. ‘Panther for females’ indicates the intervention-date representing the first week the Panther system was implemented for the female pathway: 29 May 2019.

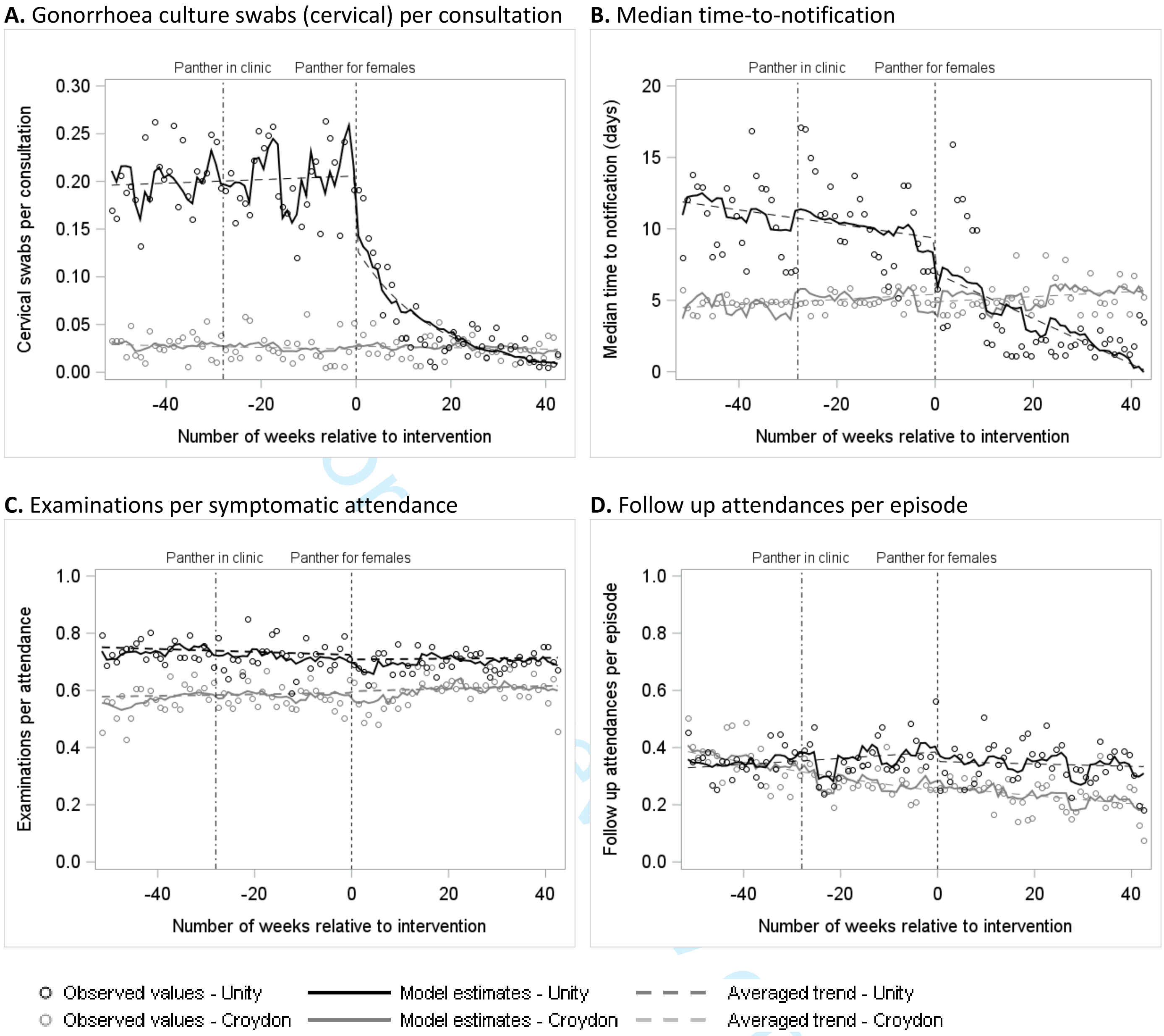

Figure 3. Modelled estimates of staff capacity for males and females combined.

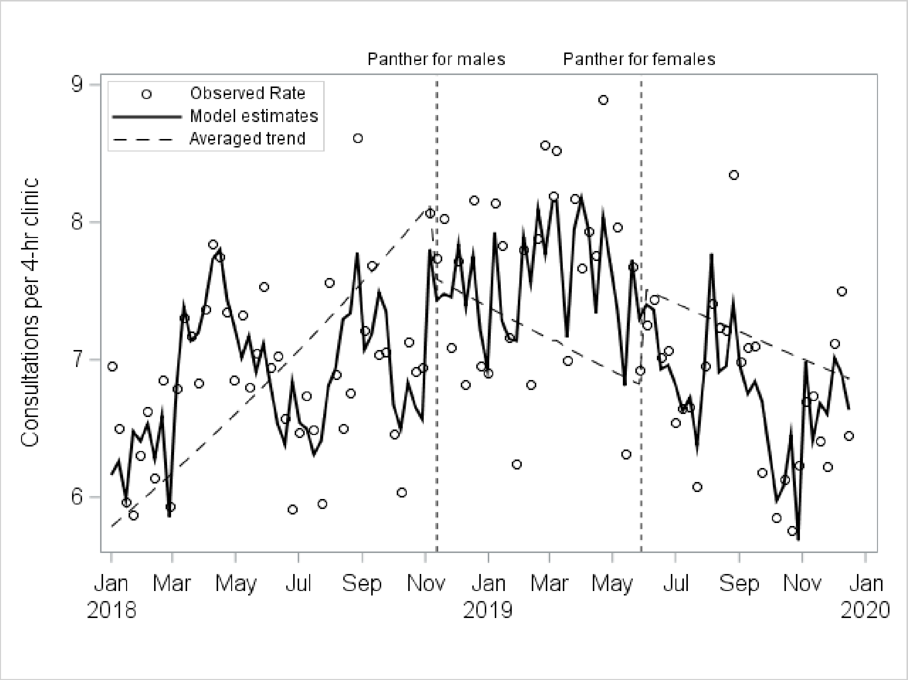

For peer review only

Supplementary material

The impact of rapid near-patient STI testing on service delivery outcomes in an integrated sexual health service in the United Kingdom: a controlled interrupted time series study

Scott R Walter, Joni Jackson, Gareth Myring, et al.

Contents:

Figure S1. Male treatment pathway before (usual care pathway) and after (rapid pathway) implementation of the Panther rapid results system.

Figure S2. Overview of female rapid treatment pathway for asymptomatic and symptomatic patients before (usual care pathway) and after (rapid pathway) implementation of the Panther rapid results system.

Table S1. Definition of examination of any type based on a combination of two examination-related variables.

Figure S3. Diagrams to illustrate controlled interrupted time series variables for estimating A) changes at the time of intervention and B) changes in trends.

Table S2. Unit costs.

Table S3. Intervention-related model estimates for females and males from sensitivity analyses using generalised additive models.

Figure S4. Modelled outcome estimates for males based on sensitivity analyses using generalised additive models.

Figure S5. Modelled outcome estimates for females based on sensitivity analyses using generalised additive models.

Figure S6. Modelled estimates of staff capacity for males and females combined.

Definition of complex cases

Definition of ethnic minority

For peer review only

Figure S1. Male treatment pathway before (usual care pathway) and after (rapid pathway) implementation of the Panther rapid results system. Reproduced from: Lorenc A, Kesten J, Brangan E, Horner PJ, Clarke M, Crofts M, Turner J, Muir P, Horwood J. What can be learnt from a qualitative evaluation of implementing a rapid sexual health testing, diagnosis and treatment service? BMJ Open, 2021; 11: e050109. doi: 10.1136/bmjopen-2021-050109.

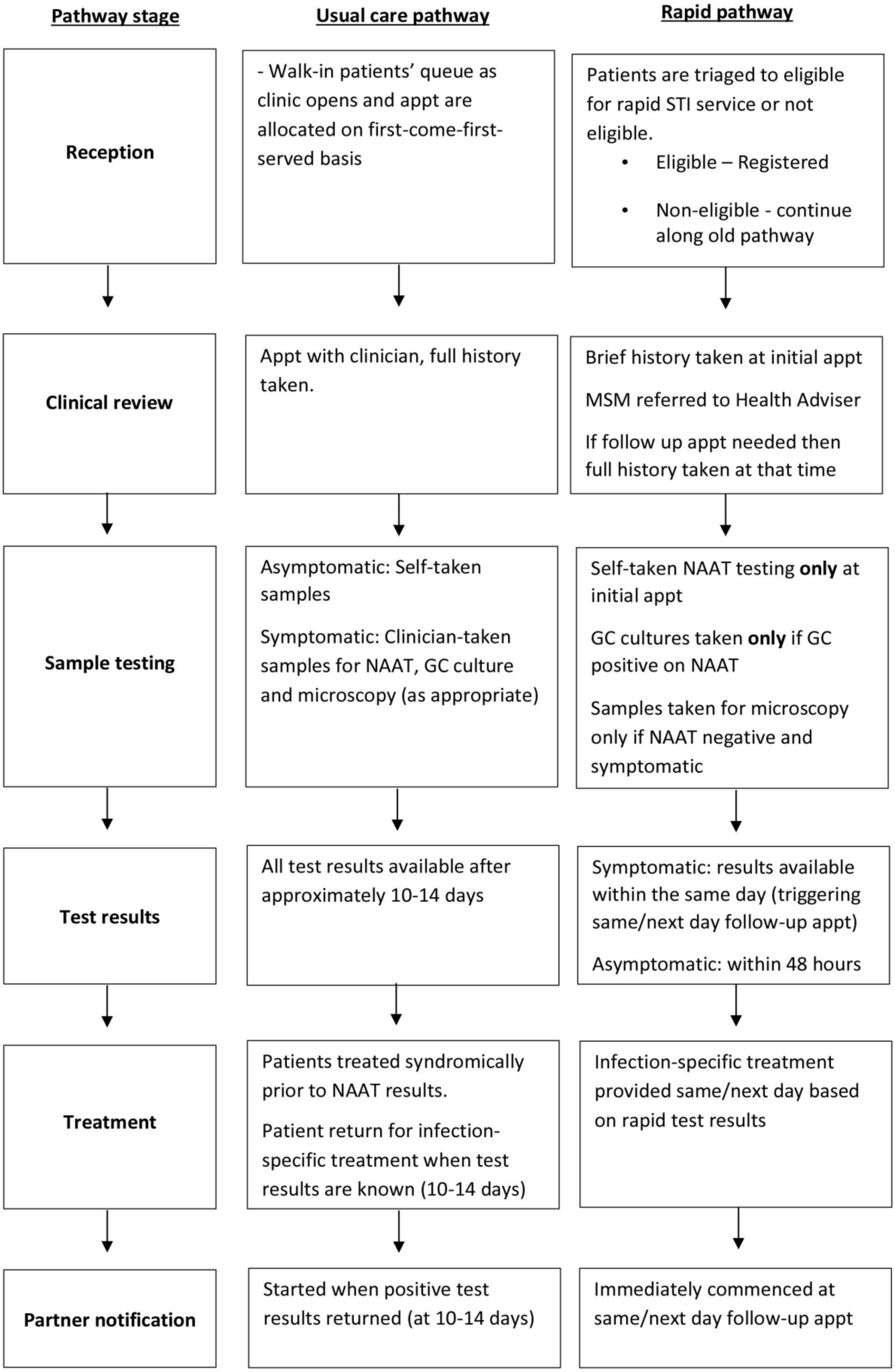

Figure S2. Overview of female rapid treatment pathway for asymptomatic and symptomatic patients before (usual care pathway) and after (rapid pathway) implementation of the Panther rapid results system. NAAT = Nucleic Acid Amplification Test GC = Gonorrhoea CT= Chlamydia, TV= *Trichomonas vaginalis*

Pathway stage	Usual care pathway	Rapid pathway: asymptomatic	Rapid pathway: symptomatic
**Reception**	Walk-in patients' queue as clinic opens and appointments are allocated on first-come-first-served basis.	Patients are triaged to rapid STI service or not:<br>● Asymptomatic and Eligible = Registered<br>● Asymptomatic and Non-eligible = continue along modified usual care pathway	Patients triaged to rapid STI service or not:<br>● Symptomatic and Eligible = Registered<br>● Symptomatic and non-eligible = modified usual care pathway (GC/CT and TV NAAT results <48 hrs) GC cultures only taken if GC NAAT positive or patient receives treatment for GC before results of GC NAAT available
**Clinical review**	Appt. with clinician, full history taken.	Brief history taken at initial appt.<br>If follow up appt. needed then full history taken at that time	History taken and no indications for visual inspection or pelvic examination required.<br>If follow up appt. needed then full history taken at that time
**Sample testing**	● Asymptomatic: Self-taken samples<br>● Symptomatic: Clinician-taken samples for CT/GC NAAT. If vaginal symptoms or pelvic pain then speculum exam and swabs taken for microscopy, TV and GC culture (as appropriate).	Self-taken NAAT testing ***only*** at initial appt<br>GC cultures taken ***only*** if GC positive on NAAT or if treatment as GC contact within 2 weeks of sexual intercourse with contact	Self-taken:<br>● Vaginal CT/GC/TV NAAT<br>● Vaginal swab for Gram staining<br>GC cultures taken ***only*** if GC positive on NAAT or if treatment as GC contact within 2 weeks of sexual intercourse with contact.
**Test results**	All test results available after approximately 10-14 days.	Results available within 48 hours.	Results of vaginal microscopy available in 20 mins and patient is then reviewed by clinician CT/GCTV NAAT results available within 48 hours
**Treatment**	Patients treated syndromically prior to NAAT results.<br>Patients return for infection-specific treatment when test results are known (10-14 days).	Infection-specific treatment provided same/next day based on rapid test results.	Infection-specific treatment provided same/next day based on rapid test results.
**Partner notification**	Started when positive test results returned (at 10-14 days).	Immediately commenced at same/next day follow-up appt.	Immediately commenced at same/next day follow-up appt.

For peer review only - http://bmjopen.bmj.com/site/about/guidelines.xhtml

Table S1. Definition of examination of any type based on a combination of two examination-related variables.

Categorical exam variable	Free text exam variable	Define as exam – MALES	Define as exam - FEMALES
Yes	Notes indicating exam	Yes	Yes
Yes	Missing	Yes	Yes
No	Notes indicating exam	Yes	Yes
No	Notes indication NO exam		
No	Missing		
External only	Notes indicating exam	Yes	Yes
External only	Notes indication NO exam		
External only	Missing		Yes
Speculum and external	Notes indicating exam	Yes	Yes
Speculum and external	Notes indication NO exam		
Speculum and external	Missing		Yes
Missing	Notes indicating exam	Yes	Yes
Missing	Notes indication NO exam		
Missing	Missing		

Notes: The categorical exam variable was intended for use with female patients but was sometimes used for males.

For peer review only

Figure S3. Diagrams to illustrate controlled interrupted time series variables for estimating A) changes at the time of intervention and B) changes in trends.

A. Changes at the time of intervention

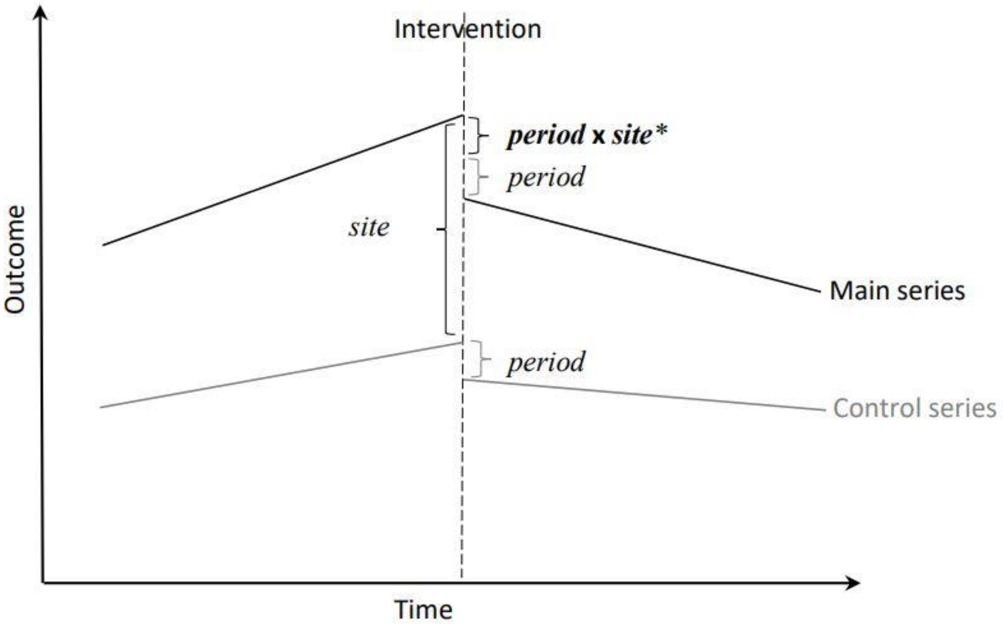

B. Trend changes

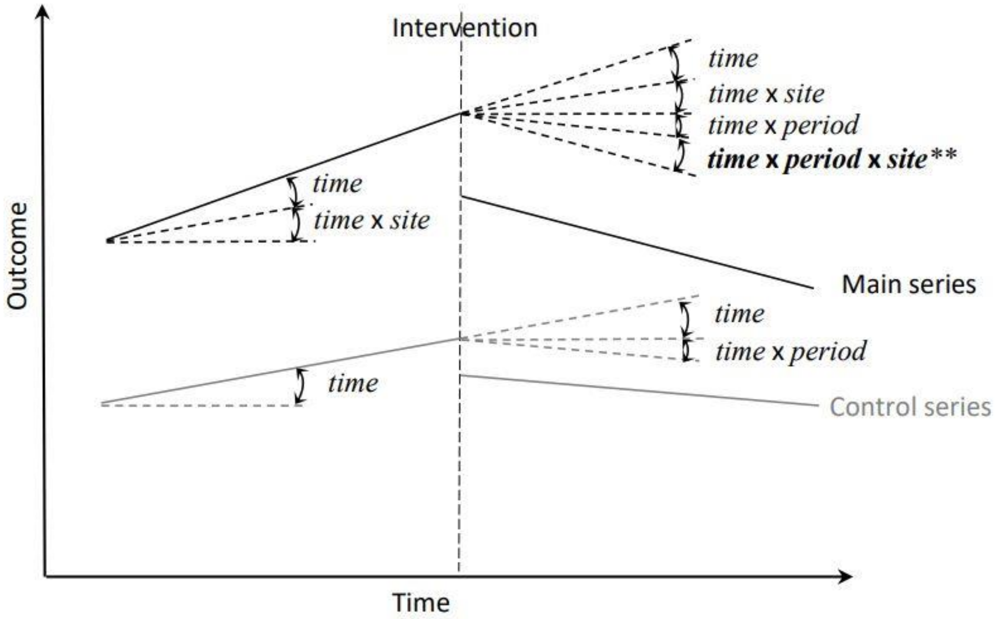

Note: *site* is a binary variable indicating either intervention or control sites; *period* is also binary indicating pre- or post-intervention periods; *time* is a continuous variable consecutively numbering each time unit (weeks in this study) with *time*=0 centred at the intervention

* *period* x *site* represents change in the intervention site at the time of intervention over and above any changes in the control site

** *time* x *period* x *site* represents change in trend for the intervention site over and above any trend changes in the control site

Table S2. Unit costs.

Unit costs of postal kit tests, and staff time[12] were from the literature and inflated to 2021 values using a UK government GDP deflator[26]. Local unit costs of diagnostic tests were provided by the intervention site. Treatment costs were from the British National Formulary[27].

Resource use	Unit cost
**Tests**	
Lab CT/GC test	£8.10
POCT CT/GC	£9.48
GC culture swab	£6.13
Male postal kit returned	£4.44
Male postal kit not returned	£3.61
Female postal kit returned	£4.08
Female postal kit not returned	£3.24
**Consultation staff time**	
Follow-up	£9.33
Male non-complex	£29.03
Male complex	£46.54
MSM complex	£42.97
Female non-complex	£29.03
Female complex	£52.26
**Treatment**	
13.5mg Levonorgestrel IUS	£69.22
6mg norelgestromin and 600micrograms ethinylestradiol	£19.51
Aciclovir 400 mg (tds for 5 Days)	£0.79
Aciclovir 400mg (bd for 6 months)	£17.64
Aciclovir 400mg (bd for 3 months)	£8.82
Aciclovir 800mg (tds for 2 days)	£0.59
Amoxicillin 250 mg tds for 5 days	£0.98
Amoxicillin 500mg	£1.01
Anusol Cream	£2.49
Anusol Ointment	£2.49
Anusol Suppositories	£1.74
Aqueous Cream BP 100g Tube	£0.77
Aqueous Cream BP 500g Tub	£3.85
Azithromycin 1g (2 x 500mg tablets)	£0.81
Azithromycin 1g (4 x 250mg capsules)	£1.24
Azithromycin 1g stat, then 500mg od for 2 days	£1.21
Azithromycin 1g stat, then 500mg od for 4 days	£2.42
Azithromycin 2g o stat	£1.62
Benzathine Benzylpenicillin 2.4 million units on day 0	£9.50
Benzathine benzylpenicillin 2.4 million units at day 7	£9.50
Benzathine benzylpenicillin 2.4 million units at day14	£9.50
Betamethasone Valerate 0.1% w/w Cream	£1.47
Betamethasone Valerate 0.1% w/w Ointment	£1.84
Betamethasone Valerate Ointment (Betnovate RD)	£1.84
Cefixime 400mg (2 x 200mg)	£26.46
Ceftriaxone 1g	£3.62
Ceftriaxone 500 mg (2 x 250mg vials)	£4.60
Chlorphenamine	£2.21
Cilest 63 tablet pack	£4.65

For peer review only

Ciprofloxacin 500mg (2 x 250mg)	£0.31
Clindamycin 300mg bd for 7 days	£17.84
Clindamycin phosphate vaginal cream	£10.86
Clobetasol Propinate (0.05% w/w) Cream (Dermovate)	£2.69
Clobetasol Propionate (0.05% w/w) Ointment	£2.69
Clobetasone Butyrate Cream (Eumovate)	£1.86
Clobetasone Butyrate Ointment (Eumovate)	£1.86
Clobetasone Butyrate, Calcium oxtertracycline & Nystatin Cream (Trimovate)	£12.45
Clotrimazole 100mg Pessary	£0.64
Clotrimazole 200mg Pessary	£1.14
Clotrimazole 500 mg Pessary	£6.99
Clotrimazole Cream 1%	£1.36
Co-Amoxiclav 250/125 (contains PENICILLIN)	£2.03
Co-amxoxiclav 500/125 (contains PENICILLIN)	£2.53
Crotamiton 10% w/w cream	£2.50
Dermol Lotion 500	£6.04
Desogestrel 75 micrograms	£2.26
Doxycycline 100mg (bd for 14 days)	£3.67
Doxycycline 100mg (bd for 21 days)	£5.51
Doxycycline 100mg (bd for 28 days)	£7.35
Doxycycline 100mg (bd for 7 days)	£2.26
Doxycycline 200mg bd for 4 weeks	£5.51
Emtricitabine 200mg & Tenofovir Disproxil 245mg	£106.00
Emtricitabine 200mg & Tenofovir Disproxil 245mg (3 days)	£10.60
Emulsifying Ointment	£4.82
Erythromycin 250 mg	£8.95
Estradiol 0.5g gel	£5.08
Estradiol 1.0mg gel	£5.85
Estradiol 10 micrograms vaginal tablet	£16.72
Femodene 63 tablet pack	£6.73
Flucloxacillin	£1.41
Fluconazole 150mg	£0.91
Fusidic acid cream	£1.92
GENTAMICIN 240mg for IM injection	£4.13
GYNAEFIX IUD	£27.11
Gardasil 0.5ml - First Dose	£86.50
Gardasil 0.5mls - Second Dose	£86.50
Gardasil 0.5mls - Third Dose	£86.50
Gedarel 20/150	£5.08
Gedarel 30/150	£5.08
Hepatitis A & B Combined Vaccine (adult)	£31.18
Hepatitis A Vaccine (2nd at 6 months)	£16.77
Hepatitis A vaccine Day 0	£16.77
Hepatitis B Vaccine (final at 6 months)	£12.20
Hepatitis B Vaccine - Dose 1 - 10mcg or 20mcg	£12.20
Hepatitis B Vaccine - Dose 2 - 10mcg or 20mcg	£12.20
Hepatitis B Vaccine - Dose 3 - 10mcg or 20mcg	£12.20
Hepatitis B Vaccine - Dose 4 - 10mcg or 20mcg	£12.20
Hepatitis B Vaccine - Extra Dose - 10mcg or 20 mcg	£12.20
Hepatitis B Vaccine 10 mcg 1 month	£12.20

For peer review only

Hepatitis B Vaccine 10 mcg 12 months	£12.20
Hepatitis B Vaccine Day 7	£12.20
Hepatits B Vaccine Day 21	£12.20
Hydro-Caine 6mls	£10.50
Hydrocortisone Cream 1%	£1.40
Hydrocortisone Ointment 1%	£1.59
Ibuprofen 200mg	£1.03
Imiquimod 5%	£48.60
Itraconazole 100mg	£3.29
Levonorgestral and Ethinylestradiol 150microgram/30microgram	£2.60
Levonorgestrel 1.5 mg	£3.65
Levonorgestrel 30 micrograms	£0.92
Levosert 52mgs IUS	£66.00
Lidocaine 4% w/w cream	£2.98
Lidocaine 5% m/m Ointment	£8.28
Lidocaine HCL 1% in 2 mls injection	£0.25
Lidocaine HCL 1% in 3.5 mls injection	£0.30
Lidocaine HCL 1% in 5 mls injection	£0.30
Lidocaine HCL 1% in 8mls for IM inj (with IM penicillin) second dose	£0.10
Lidocaine HCL 1% in 8mls for IM injection (with IM penicillin for syphilis)	£0.10
Lidocaine HCL 1% in 8mls for IM injection (with IM penicillin) third dose	£0.10
Lidocaine HCL 2% in 2 mls injection	£0.27
Lidocaine HCL 2% in 5 mls injection	£0.32
Lignocaine 2% Gel	£2.99
Loestrin 20 63 Tablet Pack	£1.99
Loestrin 30 63 Tablet Pack	£1.99
Logynon	£2.60
Marvelon 63 Tablet Pack	£7.10
Mebendazole 100mg	£2.66
Medroxyprogesterone Acetate 104mg in 0.65mls sub cutaneous	£6.90
Medroxyprogesterone Acetate 150mg in 1ml	£6.01
Mefenamic Acid 250mg	£8.17
Mepivacaine Hydrochloride 3%	£0.44
Mepivicaine 3% in 2.2mls	£0.44
Mercilon 63 Tablet Pack	£8.44
Metronidazole 0.75% Vaginal Gel	£4.31
Metronidazole 2g stat dose (400 mg x 5)	£0.52
Metronidazole 400mg (bd for 5 days)	£1.03
Metronidazole 400mg bd for 10 days	£2.07
Miconazole Nitrate 2%w/w, hydrocortisone 1%w/w Cream (Daktocourt)	£2.49
Miconazole Nitrate Cream 20mg/g (Gyno-Daktarin)	£4.33
Miconazole nitrate 20mg per g	£4.33
Millinette 20/75	£5.41
Millinette 30/75	£4.12
Mini TT 380	£12.46
Mirena 52mg IUS	£88.00
Moxifloxacin 400mg od for 10 days	£19.08
Moxifloxacin 400mg od for 14 days	£26.71
Nexplanon 68mg implant	£83.43
Nitrofurantoin 50mg o qds 7 days	£5.08
Nitrofurantoin 50mg o qds for 3 days	£2.18

For peer review only

Nonoxinol-9	£11.00
Norethisterone 350 micrograms	£2.10
Norethisterone 350 micrograms 84 Tablet Pack	£2.10
Norimin 63 Tablet Pack	£2.28
Nova T 380	£15.20
Ofloxacin 200mg (one tablet twice daily for 14 days)	£12.54
Ofloxacin 200mg (one tablet twice daily for 7 days)	£6.27
Ofloxacin 200mg (two tablets twice daily for 14 days)	£25.09
Paediatric Hepatitis B Vaccine - Dose 1 - 10mcg	£12.20
Paediatric Hepatitis B Vaccine - Dose 2 - 10 mcg	£12.20
Paediatric Hepatitis B Vaccine - Dose 3 - 10 mcg	£12.20
Paediatric Hepatitis B Vaccine - Dose 4 - 10mcg	£12.20
Paracetamol 500mg	£0.86
Permethrin 5% w/w cream	£8.54
Podophyllotoxin 0.15% Cream	£17.83
Podophyllotoxin 0.5% Solution	£14.49
Raltegravir 400 mg bd for 3 days	£47.14
T- Safe 380A QL	£10.55
TT 380 Slimline	£12.46
Terbinafine Hydrochloride 1% Cream	£2.39
Trimethoprim 200mg	£1.16
Ulipristal Acetate 30mg	£14.05
Xylocaine 1% with adrenaline 1 :200,000	£1.77

For peer review only

Table S3. Intervention-related model estimates for females and males from sensitivity analyses using generalised additive models.

Outcome	Change at time of intervention (95% CI)	P-value for post-panther non-linearity of intervention site data
**MALES – 12th November 2018**		
Gonorrhoea culture swabs per consultation	-16.6% (-30.1%, -0.5%)	<0.001
Time to notification	+0.4 days (+0.27, +0.71)	0.03
**FEMALES – 29th May 2019**		
Gonorrhoea culture swabs per consultation	-11.1% (-29.8%, +12.6%)	<0.001
Time to notification	-0.16 days (-0.42%, +0.10)	<0.001

For peer review only

Figure S4. Modelled outcome estimates for males based on sensitivity analyses using generalised additive models. Both the overall time trend and the post-panther intervention site trend were estimated as splines with three degrees of freedom. All other covariates treated as in the main analysis.

A. Gonorrhoea culture swabs (urethral) per consultation

B. Median time to notification

C. Examinations per symptomatic attendance

D. Follow up attendances per episode

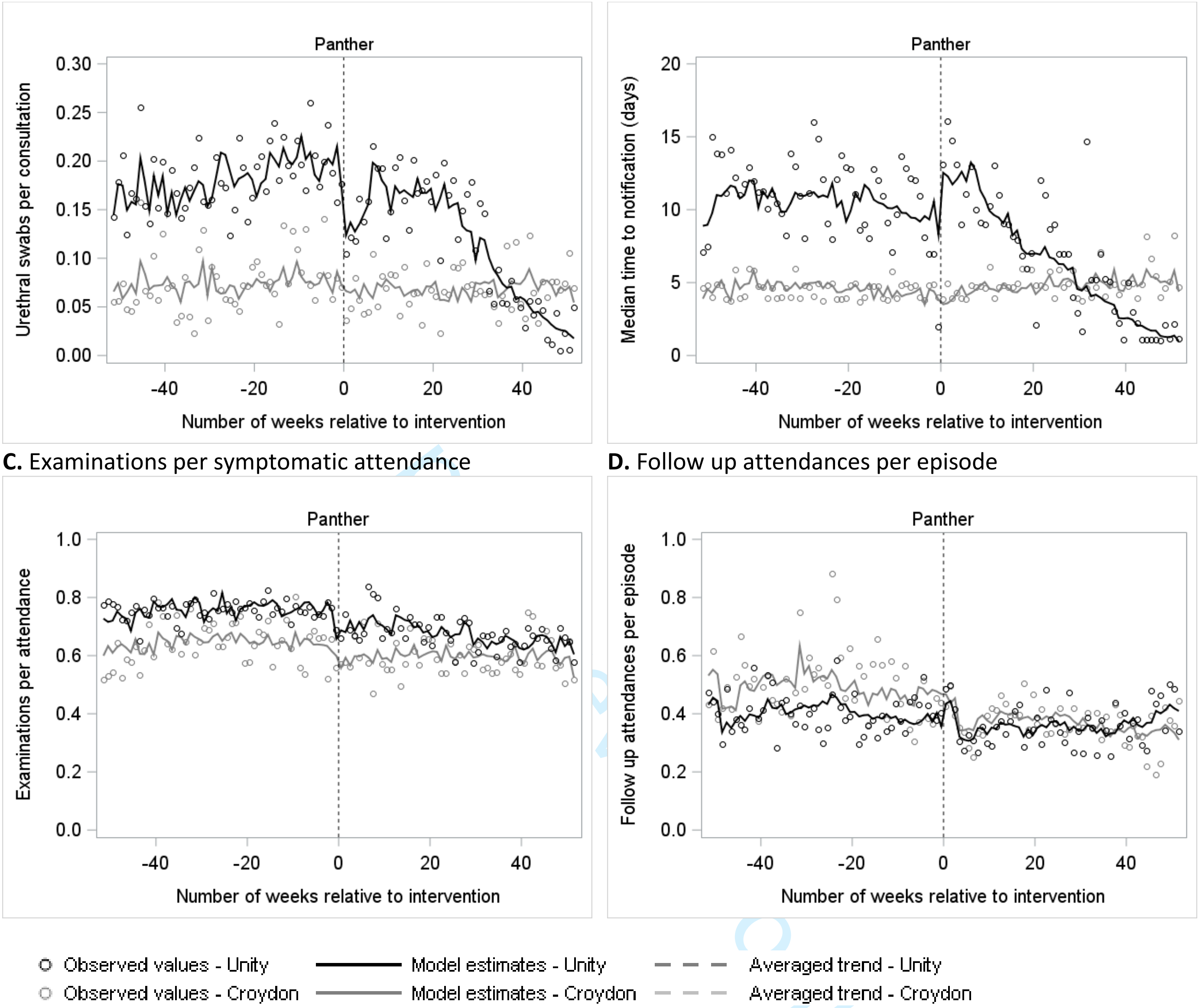

Figure S5. Modelled outcome estimates for females based on sensitivity analyses using generalised additive models. Both the overall time trend and the post-panther intervention site trend were estimated as splines with three degrees of freedom. All other covariates treated as in the main analysis.

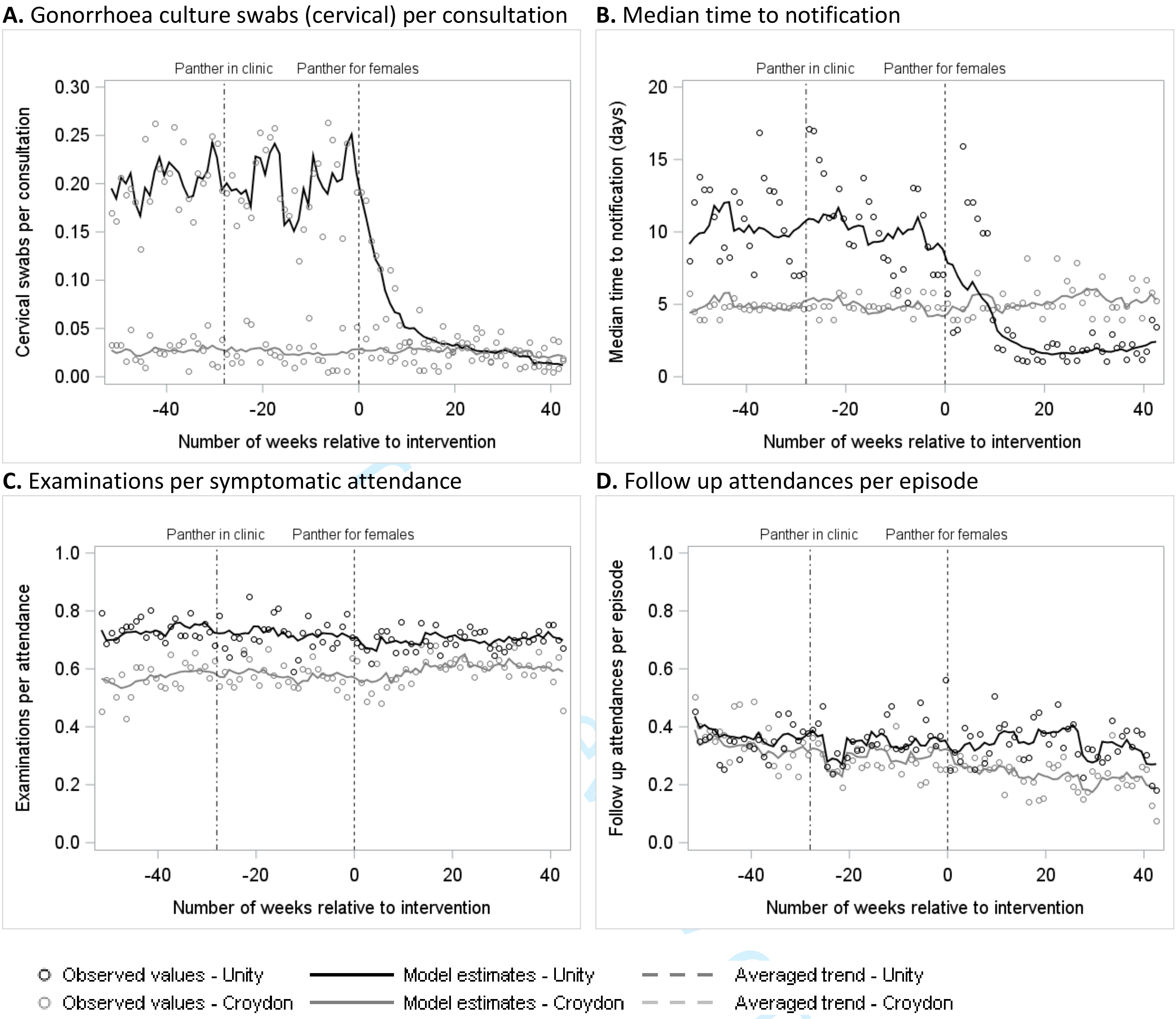

Figure S6. Modelled estimates of staff capacity for males and females combined. Time trends modelled with splines to allow for non-linearity. All other covariates treated as in the main analysis.

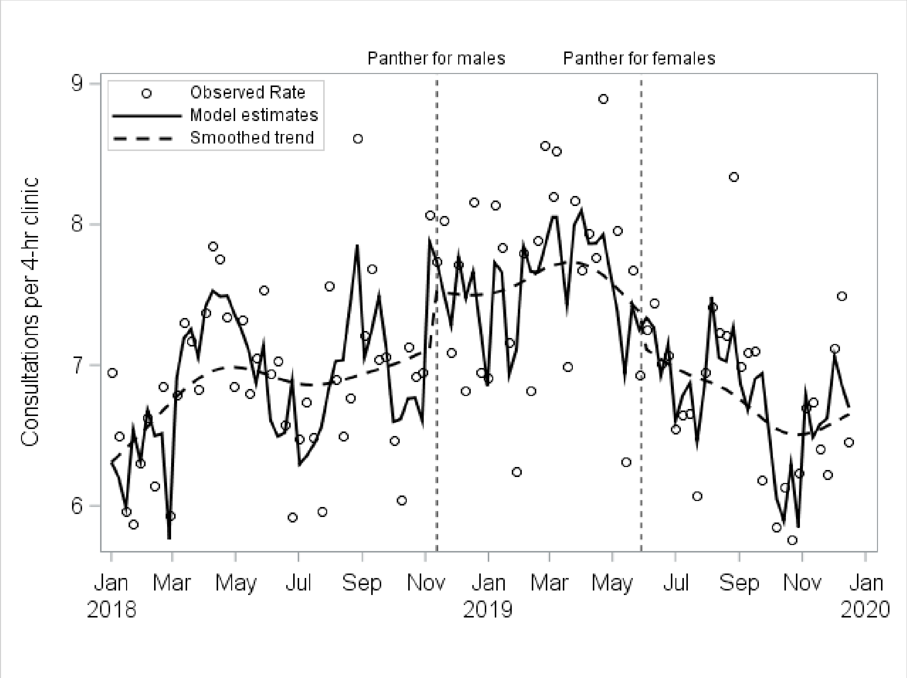

For peer review only

Definition of complex cases

Criteria for all patients:

a. Patients under 18 years of age
b. Have been/are currently exposed to child sexual exploitation, domestic violence, sexual assault
c. Has a current record of substance misuse
d. Has a current diagnosis of syphilis
e. Has current multiple diagnoses clinical diagnoses (GUMCAD coding B &/or C)
f. Has a history of/current diagnosis of genital herpes or had a swab taken for genital herpes
g. Has had post exposure prophylaxis after sexual exposure to HIV (PEPSE)
h. Needed an interpreter/use of translation service
i. Has current diagnosis of D2B on GUMCAD

Additional criteria for females:

i. Receive contraceptive care
ii. experienced pelvic pain, dyspareunia or post coital bleeding
iii. are pregnant
iv. experienced female genital mutilation.

Additional criteria for males:

v. are bisexual
vi. has sex with men
vii. Experienced testicular pain
viii. has a history/current record of chronic pelvic syndrome

For peer review only

Definition of an ethnic minority

This is self-reported at patient registration using the nationally-defined categories embedded within the patient record system.

Categories included in our definition are:

African

Caribbean

Any other black background

White and black Caribbean

White and black African

Indian

Pakistani

Bangladeshi

Any other Asian background

White and Asian

Chinese

Any other mixed background

Any other ethnic group

For peer review only

The RECORD statement – checklist of items, extended from the STROBE statement, that should be reported in observational studies using routinely collected health data.

	Item No.	STROBE items	Location in manuscript where items are reported	RECORD items	Location in manuscript where items are reported
**Title and abstract**					
	1	(a) Indicate the study's design with a commonly used term in the title or the abstract (b) Provide in the abstract an informative and balanced summary of what was done and what was found	(a) p.1<br>(b) p.2	RECORD 1.1: The type of data used should be specified in the title or abstract. When possible, the name of the databases used should be included.<br><br>RECORD 1.2: If applicable, the geographic region and timeframe within which the study took place should be reported in the title or abstract.<br><br>RECORD 1.3: If linkage between databases was conducted for the study, this should be clearly stated in the title or abstract.	1.1, abstract p.2<br><br>1.2, abstract p.2<br><br>N/A
**Introduction**					
Background rationale	2	Explain the scientific background and rationale for the investigation being reported			Introduction pp.4-5
Objectives	3	State specific objectives, including any prespecified hypotheses			End of introduction p.5
**Methods**					
Study Design	4	Present key elements of study design early in the paper			Section 2.1, p.5
Setting	5	Describe the setting, locations, and relevant dates, including periods of recruitment, exposure, follow-up, and data collection			Methods pp.5-7

For peer review only

Participants	6	*(a) Cohort study* - Give the eligibility criteria, and the sources and methods of selection of participants. Describe methods of follow-up<br>*Case-control study* - Give the eligibility criteria, and the sources and methods of case ascertainment and control selection. Give the rationale for the choice of cases and controls<br>*Cross-sectional study* - Give the eligibility criteria, and the sources and methods of selection of participants<br><br>*(b) Cohort study* - For matched studies, give matching criteria and number of exposed and unexposed<br>*Case-control study* - For matched studies, give matching criteria and the number of controls per case		RECORD 6.1: The methods of study population selection (such as codes or algorithms used to identify subjects) should be listed in detail. If this is not possible, an explanation should be provided.<br><br>RECORD 6.2: Any validation studies of the codes or algorithms used to select the population should be referenced. If validation was conducted for this study and not published elsewhere, detailed methods and results should be provided.<br><br>RECORD 6.3: If the study involved linkage of databases, consider use of a flow diagram or other graphical display to demonstrate the data linkage process, including the number of individuals with linked data at each stage.	Table 1<br><br>N/A<br><br>N/A
Variables	7	Clearly define all outcomes, exposures, predictors, potential confounders, and effect modifiers. Give diagnostic criteria, if applicable.		RECORD 7.1: A complete list of codes and algorithms used to classify exposures, outcomes, confounders, and effect modifiers should be provided. If these cannot be reported, an explanation should be provided.	Outcomes – table 1<br>Confounders – section 2.5, pp.7-8
Data sources/ measurement	8	For each variable of interest, give sources of data and details of methods of assessment (measurement).<br>Describe comparability of assessment methods if there is more than one group			Sections 2.4 & 2.5, pp.6-9

For peer review only

Bias	9	Describe any efforts to address potential sources of bias			Section 2.5, pp.7-8
Study size	10	Explain how the study size was arrived at			Section 2.4, p.6
Quantitative variables	11	Explain how quantitative variables were handled in the analyses. If applicable, describe which groupings were chosen, and why			Sections 2.5 and 2.6, pp.7-9 Supplement Table S1
Statistical methods	12	(a) Describe all statistical methods, including those used to control for confounding<br>(b) Describe any methods used to examine subgroups and interactions<br>(c) Explain how missing data were addressed<br>(d) *Cohort study* - If applicable, explain how loss to follow-up was addressed<br>*Case-control study* - If applicable, explain how matching of cases and controls was addressed<br>*Cross-sectional study* - If applicable, describe analytical methods taking account of sampling strategy<br>(e) Describe any sensitivity analyses			Sections 2.5 and 2.6, pp.7-9
Data access and cleaning methods		..		RECORD 12.1: Authors should describe the extent to which the investigators had access to the database population used to create the study population.	12.1: Section 2.4, p.6<br><br>12.2: Section 2.4, p.7

For peer review only

				RECORD 12.2: Authors should provide information on the data cleaning methods used in the study.	
Linkage		..		RECORD 12.3: State whether the study included person-level, institutional-level, or other data linkage across two or more databases. The methods of linkage and methods of linkage quality evaluation should be provided.	N/A
**Results**					
Participants	13	(a) Report the numbers of individuals at each stage of the study (*e.g.*, numbers potentially eligible, examined for eligibility, confirmed eligible, included in the study, completing follow-up, and analysed)<br>(b) Give reasons for non-participation at each stage.<br>(c) Consider use of a flow diagram		RECORD 13.1: Describe in detail the selection of the persons included in the study (*i.e.,* study population selection) including filtering based on data quality, data availability and linkage. The selection of included persons can be described in the text and/or by means of the study flow diagram.	Results, first paragraph, p.9
Descriptive data	14	(a) Give characteristics of study participants (*e.g.*, demographic, clinical, social) and information on exposures and potential confounders<br>(b) Indicate the number of participants with missing data for each variable of interest<br>(c) *Cohort study* - summarise follow-up time (*e.g.*, average and total amount)			Results, first paragraph p.9 and Table 2.
Outcome data	15	*Cohort study* - Report numbers of outcome events or summary measures over time<br>*Case-control study* - Report numbers in each exposure			Table 2

For peer review only

		category, or summary measures of exposure<br>*Cross-sectional study* - Report numbers of outcome events or summary measures			
Main results	16	(a) Give unadjusted estimates and, if applicable, confounder-adjusted estimates and their precision (e.g., 95% confidence interval). Make clear which confounders were adjusted for and why they were included<br>(b) Report category boundaries when continuous variables were categorized<br>(c) If relevant, consider translating estimates of relative risk into absolute risk for a meaningful time period			Sections 3.1-3.3 (pp.9-11) and Tables 3 & 4.
Other analyses	17	Report other analyses done—e.g., analyses of subgroups and interactions, and sensitivity analyses			Sections 3.1-3.3 (pp.9-11) and supplement table S3, figures S4-S6.
**Discussion**					
Key results	18	Summarise key results with reference to study objectives			pp.11-13
Limitations	19	Discuss limitations of the study, taking into account sources of potential bias or imprecision. Discuss both direction and magnitude of any potential bias		RECORD 19.1: Discuss the implications of using data that were not created or collected to answer the specific research question(s). Include discussion of misclassification bias, unmeasured confounding, missing data, and changing eligibility over time, as they pertain to the study being reported.	Section 4.1, pp13-14.
Interpretation	20	Give a cautious overall interpretation of results considering objectives,			Section 4.2, pp.14-15

		limitations, multiplicity of analyses, results from similar studies, and other relevant evidence			
Generalisability	21	Discuss the generalisability (external validity) of the study results			Section 4.2, pp.14-15
**Other Information**					
Funding	22	Give the source of funding and the role of the funders for the present study and, if applicable, for the original study on which the present article is based			p.16
Accessibility of protocol, raw data, and programming code		..		RECORD 22.1: Authors should provide information on how to access any supplemental information such as the study protocol, raw data, or programming code.	See data statement, p.16

*Reference: Benchimol EI, Smeeth L, Guttmann A, Harron K, Moher D, Petersen I, Sørensen HT, von Elm E, Langan SM, the RECORD Working Committee. The REporting of studies Conducted using Observational Routinely-collected health Data (RECORD) Statement. *PLoS Medicine* 2015; in press.

*Checklist is protected under Creative Commons Attribution (CC BY) license.

For peer review only

BMJ Open

The impact of rapid near-patient STI testing on service delivery outcomes in an integrated sexual health service in the United Kingdom: a controlled interrupted time series study

Journal:	*BMJ Open*
Manuscript ID	bmjopen-2022-064664.R2
Article Type:	Original research
Date Submitted by the Author:	13-Dec-2022
Complete List of Authors:	Walter, Scott; University of Bristol Medical School, NIHR Applied Research Collaboration West<br>Jackson, Joni; NIHR ARC West, ARC West; University of Bristol Medical School, Populations Health Sciences<br>Myring, Gareth; University of Bristol School of Social and Community Medicine<br>Redaniel, Maria Theresa; University of Bristol, NIHR CLAHRC West; University of Bristol, School of Social and Community Medicine<br>Margelyte, Ruta; University of Bristol, School of Social and Community Medicine; NIHR Collaboration for Leadership in Applied Health Research and Care West,<br>Gardiner, Rebecca; University Hospitals Bristol and Weston NHS Foundation Trust, Unity Sexual Health; University Hospitals Bristol and Weston NHS Foundation Trust, Bristol Haematology and Oncology Centre<br>Clarke, Michael; University Hospitals Bristol and Weston NHS Foundation Trust, Unity Sexual Health<br>Crofts, Megan; University Hospitals Bristol and Weston NHS Foundation Trust, Unity Sexual Health<br>McLeod, Hugh; University of Bristol<br>Hollingworth, William; University of Bristol, School of Social and Community Medicine<br>Phillips, David; Croydon University Hospital, Croydon Sexual Health<br>Muir, Peter; Public Health England, Specialist Virology Centre, Public Health Laboratory Bristol<br>Steer, Jonathan; Public Health England, Bristol Public Health Laboratory<br>Turner, Jonathan; Public Health England, Bristol Public Health Laboratory<br>Horner, Paddy; University of Bristol, School of Social and Community Medicine; University Hospitals Bristol NHS Fouindation Trust, Bristol Sexual Health Centre<br>De Vocht, Frank; University of Bristol,
**Primary Subject Heading**:	Sexual health
Secondary Subject Heading:	Health services research
Keywords:	SEXUAL MEDICINE, Epidemiology < INFECTIOUS DISEASES, HEALTH ECONOMICS

SCHOLARONE™
Manuscripts

I, the Submitting Author has the right to grant and does grant on behalf of all authors of the Work (as defined in the below author licence), an exclusive licence and/or a non-exclusive licence for contributions from authors who are: i) UK Crown employees; ii) where BMJ has agreed a CC-BY licence shall apply, and/or iii) in accordance with the terms applicable for US Federal Government officers or employees acting as part of their official duties; on a worldwide, perpetual, irrevocable, royalty-free basis to BMJ Publishing Group Ltd ("BMJ") its licensees and where the relevant Journal is co-owned by BMJ to the co-owners of the Journal, to publish the Work in this journal and any other BMJ products and to exploit all rights, as set out in our licence.

The Submitting Author accepts and understands that any supply made under these terms is made by BMJ to the Submitting Author unless you are acting as an employee on behalf of your employer or a postgraduate student of an affiliated institution which is paying any applicable article publishing charge ("APC") for Open Access articles. Where the Submitting Author wishes to make the Work available on an Open Access basis (and intends to pay the relevant APC), the terms of reuse of such Open Access shall be governed by a Creative Commons licence – details of these licences and which Creative Commons licence will apply to this Work are set out in our licence referred to above.

Other than as permitted in any relevant BMJ Author's Self Archiving Policies, I confirm this Work has not been accepted for publication elsewhere, is not being considered for publication elsewhere and does not duplicate material already published. I confirm all authors consent to publication of this Work and authorise the granting of this licence.

For peer review only

The impact of rapid near-patient STI testing on service delivery outcomes in an integrated sexual health service in the United Kingdom: a controlled interrupted time series study

Scott R Walter[1,2], Joni Jackson[1,2], Gareth Myring[1,2], Maria Theresa Redaniel[1,2], Ruta Margelyte[1,2], Rebecca Gardiner[3,4], Michael D Clarke[4], Megan Crofts[4], Hugh McLeod[1,2], William Hollingworth[1,2], David Phillips[5], Peter Muir[6,7], Jonathan Steer[6], Jonathan Turner[6], Paddy J Horner[2,3,7]*, Frank de Vocht[1,2]*

*joint last authors

Affiliations

1. National Institute for Health Research, Applied Research Collaboration West (NIHR ARC West) University Hospitals Bristol and Weston NHS Foundation Trust, Bristol, UK.
2. Population Health Sciences, Bristol Medical School, University of Bristol, Bristol, UK.
3. Unity Sexual Health, University Hospitals Bristol and Weston NHS Foundation Trust, Bristol, UK.
4. Bristol Haematology and Oncology Centre, University Hospitals Bristol and Weston NHS Foundation Trust, Bristol, UK.
5. Croydon Sexual Health
6. Southwest Regional Laboratory, UK Health Security Agency, North Bristol NHS Trust, Bristol, UK.
7. National Institute for Health Research, Health Protection Research Unit in Evaluation of Interventions, University of Bristol, Bristol, UK.

Keywords: sexual health; chlamydia; gonorrhoea; rapid STI testing

Corresponding author:
Scott R Walter
National Institute for Health Research
Applied Research Collaboration West (NIHR ARC West)
University Hospitals Bristol and Weston NHS Foundation Trust
9th Floor, Whitefriars, Lewins Mead
Bristol, BS1 2NT, UK
scott.walter@bristol.ac.uk

Word count: 3768

For peer review only

For peer review only - http://bmjopen.bmj.com/site/about/guidelines.xhtml

Abstract

Objectives: To evaluate the impact of a new clinic-based rapid STI testing, diagnosis and treatment service on healthcare delivery and resource needs in an integrated sexual health service.

Design: Controlled interrupted time series study.

Setting: Two integrated sexual health services in UK: Unity Sexual Health in Bristol, UK (intervention site) and Croydon Sexual Health in London (control site).

Participants: Electronic patient records for all 58,418 attendances during the period one year before and one year after the intervention.

Intervention: Introduction of an in-clinic rapid testing system for gonorrhoea and chlamydia in combination with revised treatment pathways.

Outcome measures: Time-to-test notification, staff capacity, cost per episode of care and overall service costs. We also assessed rates of gonorrhoea culture swabs, follow-up attendances, and examinations.

Results: Time-to-notification and the rate of gonorrhoea swabs significantly decreased following implementation of the new system. There was no evidence of change in follow-up visits or examination rates for patients seen in clinic related to the new system. Staff capacity in clinics appeared to be maintained across the study period. Overall, the number of episodes per week was unchanged in the intervention site, and the mean cost per episode decreased by 7.5% (95%CI 5.7%, 9.3%).

Conclusions: The clear improvement in time-to-notification, while maintaining activity at a lower overall cost, suggests that the implementation of clinic-based testing had the intended impact, which bolsters the case for more widespread rollout in SHS.

For peer review only - http://bmjopen.bmj.com/site/about/guidelines.xhtml

Strengths and limitations of this study

- We used controlled interrupted time series models with confounder adjustment to estimate the effect of the intervention distinct from any background changes and independent of other time varying factors.
- Model validity was bolstered by using a relatively long time series with good temporal resolution.
- Data from both the main and control sites was derived from the same electronic patient record system.
- There was a general consensus between main and sensitivity analyses.
- Our study was limited by being non-randomised, having only one control site, and the follow up period for females being truncated by the impact of the Covid-19 pandemic.

For peer review only

For peer review only - http://bmjopen.bmj.com/site/about/guidelines.xhtml

1. Introduction

Sexually transmitted infection (STI) diagnoses are increasing in England with more than a 10% increase in new infections between 2016 and 2019[1]. Over the same period, a 19.2% increase in total consultations at sexual health services (SHS) was reported in England[2]. Open-access SHS providing rapid treatment and partner notification can reduce the risk of STI complications and infection spread[3,4,5]. Public Health England (now UK Health Security Agency [UKHSA]) recommends that local SHS need to be available to both the general population and groups with greater sexual health needs[3]. Nevertheless, the central government's public health grant, including SHS funding, has steadily decreased since 2015[6,7]. Despite diminishing resources, continued provision of SHS has been achieved through increased efficiencies at clinic-based services and introduction of online services[8,9].

Another approach to improving efficiency while ensuring quality, could be the introduction of near-patient testing (NPT) for chlamydia and gonorrhoea. That is, testing where samples are taken at the time of consultation and results returned within a short timeframe (immediately or within hours). Potential benefits include earlier diagnosis and treatment, reduced risk of sequelae and onward transmission, and reduction in unnecessary treatments, as well as reduced costs and clinician time due to reduction in the need for gonorrhoea cultures, examinations and follow-up visits[10,11,12]. Although modelling studies suggest NPT can be cost-effective, this remains to be demonstrated in practice[10-14]. Research also suggests that reduced waiting times for STI test results may enhance patient acceptability[15,16] and increase testing uptake[17,18]. Importantly, patients have expressed preferences for earlier provision of results[19] due to the stress of waiting[20].

In November 2018, Unity Sexual Health (hereafter *the intervention site*), a UK specialist integrated SHS, implemented a rapid nucleic acid amplification (NAAT) STI testing, diagnosis and treatment service for chlamydia and gonorrhoea, using the Hologic 'Panther' diagnostic platform in a clinic-based satellite laboratory[21]. It can deliver results in 3.5 hours by eliminating sample batching

For peer review only

and transit times associated with microbiology laboratory testing. Integrated sexual health services provide the full range of contraception services in addition to STI and blood borne virus testing, treatment and management and health promotion and prevention.[22]

We used a quantitative approach to evaluate the impact of the new rapid testing process on service delivery and resource needs of the intervention site.

2. Methods

2.1 Setting and design

The intervention site is a provider of integrated SHS in the Bristol area of the United Kingdom, with about 40,000 attendances annually. In addition to in-clinic services, self-testing kits for chlamydia, gonorrhoea, syphilis and HIV ordered online by patients are provided by post. This postal testing kit service was provided by the intervention site for asymptomatic patients through its dedicated website and used the same NAAT testing platform as the rapid STI service. This was in place prior to the intervention and was increasingly used throughout the study period.

This study is a quasi-experimental, controlled interrupted time series (CITS) design that used routinely collected electronic patient record (EPR) data. The intervention time points were defined differently for males and females: rapid STI testing was introduced on 12 November 2018 for males and 29 May 2019 for females.

For peer review only

2.2 Rapid STI service model

Eligibility criteria and treatment pathways differed for males and females. A graphical overview of each pathway is provided in the supplement (Figures S1 and S2) with pre-intervention pathway included for reference. Additional changes were made to the SHS related to staff capacity. Rapid STI asymptomatic consultations were reduced to 15 minutes, while the number of allocated patients per staff member for the walk-in clinic remained the same.

For peer review only - http://bmjopen.bmj.com/site/about/guidelines.xhtml

2.2.1 Rapid STI testing

The collection, processing and analysis of specimens with the Aptima Combo 2 (Hologic) NAAT at the intervention site, which detects both *Chlamydia trachomatis* and *Neisseria gonorrhoeae* and the Aptima TV *Trichomonas vaginalis* NAAT, followed the manufacturer's instructions and national guidelines. Quality control measures were the same as those in the central UKHSA South West Regional Laboratory and complied with national standards. The testing was undertaken by a dedicated technician employed by UKHSA experienced in using the Hologic Panther platform (further details in supplement).

2.2.2 Males

Male patients were eligible for the rapid STI pathway if they were asymptomatic or had urethritis symptoms. If asymptomatic, a brief history was taken prior to patient self-sampling for chlamydia and gonorrhoea and taking blood tests for HIV and syphilis. Men who have sex with men (MSM) were referred to a health adviser for health promotion, including discussion about testing for HIV and other STIs, and safer sex practices. Symptomatic men were asked to return four hours later when NAAT results were available. If positive, they received infection specific treatment; if negative a urethral smear was undertaken to diagnose non-gonococcal urethritis. Contacts of patients with gonorrhoea or chlamydia outside a two-week window were treated if NAAT-positive. Swabs for gonococcal culture and sensitivities were only taken after a NAAT-positive result for gonorrhoea or if gonococcal treatment was administered prior to the NAAT result.

2.2.3 Females

Female asymptomatic patients without contraception needs were eligible for the rapid drop-off service. Women with abnormal vaginal discharge, not requiring bimanual or speculum examination to exclude pathology, self-swabbed and were treated on the results of microscopy and clinical findings at the time of visit and informed that chlamydia and gonorrhoea NAAT test results would be

For peer review only - http://bmjopen.bmj.com/site/about/guidelines.xhtml

available within 48 hours. They were termed symptomatic. For contraceptive needs, a clinical consultation was necessary to determine the need for examination. Trichomonas vaginalis (TV) culture was replaced with a more sensitive TV NAAT[23], also available within 48 hours. A gonococcal culture swab was only taken after a NAAT-positive result for gonorrhoea or if gonococcal treatment was administered prior to NAAT result.

2.3 Control site

Croydon Sexual Health, a similar integrated SHS in South London, was used as the control site to account for background changes unrelated to the intervention. This site has similar patient throughput (about 32,000 annual attendances) and uses the same EPR system.

2.4 Data

Fully anonymised individual patient data extracted from the intervention and control site EPR systems[23] comprised demographic information, sexual behaviour, mode of presentation and attendances to the clinic, diagnostic testing and treatment. Analyses were based on a census of attendance level records.

Time-to-notification was defined from the text message notification system[24]. This included text message type for identifying test results messages, time stamps and anonymised patient identifiers. Numbers of NAAT postal testing kits were extracted from the intervention site's records, while the control site did not implement these until after the study period.

Prior to analysis, data were checked for duplicates, implausible values and missingness. Individual variables were combined to generate indicator variables for complex cases, MSM, examinations, ethnic minority status. All time-related variables were derived from the date and time of each attendance.

For analysis, data were aggregated at weekly level over a two-year period centred at the intervention. For females, data were excluded from the first UK Covid-19-related lockdown (23

For peer review only - http://bmjopen.bmj.com/site/about/guidelines.xhtml

March 2020) due to changes in outcomes that could not be adequately accounted for in models. The study period for males was from 13 November 2017 to 10 November 2019, and for females 28 May 2018 to 22 March 2020.

2.5 Statistical analysis

There main study outcomes are detailed in Table 1. CITS models within a generalised linear modelling framework were applied to each outcome separately for males and females: ten models in total. $Time$ was modelled as linear using consecutively numbered weeks, with $time = 0$ at the intervention point. A binary variable ($period$) representing pre- and post-intervention periods was defined by the respective male and female intervention dates.

Gonorrhoea culture swabs per consultation, follow-up attendances per care episode, examinations per symptomatic attendance and staff capacity were modelled as rates assuming a negative binomial distribution. These models generate rate ratios, presented as percentage changes. For time-to-notification, a normal distribution was assumed and results presented as differences in median time (days). This represents absolute measure of time including weekends as opposed to working days only.

Table 1. Definitions of main study outcomes.

Outcome measure	Definition
1. Rate of gonorrhoea culture swabs per consultation	Numerator: the number of GC swabs, urethral for male and cervical for female<br>Denominator: the number of consultations where these were defined as attendances for new, rebooked or walk-in patients
2. Time-to-notification	Median time from sample collection until the patient was notified of the test result via text message
3. Rate of examinations per symptomatic attendance	Numerator: the number of examinations of any type. This was based on a combination of variables used to record information about examinations (supplementary Table S1)<br>Denominator: all attendances where the patient was recorded as being symptomatic
4. Rate of follow up attendances per episode of care	Numerator: the number of follow up attendances occurring within 30 days of an initial consultation<br>Denominator: the number of episodes involving at least 1 consultation

For peer review only - http://bmjopen.bmj.com/site/about/guidelines.xhtml

5. Staff capacity – rate of patients seen per four-hour clinic	Numerator: number of patient consultations (any new, rebooked, walk-in or follow up attendance)<br>Denominator: number staff available for four-hour clinics

The main variables in the models were $time$, $period$ and $site$ (intervention vs. control) along with all two-way and three-way interactions, as per a CITS approach for estimating both a step change and slope change[25,26]. Two key terms in the models represent intervention-related changes over and above any control site changes. The interaction $period \times site$ captures a differential step change for the intervention site compared to control site. While the three-way interaction term $time \times period \times site$ captures different degrees of pre-post trend change for the intervention site compared to control site (supplement Figure S3).

Additional covariates were included in the models: proportions of complex patients, symptomatic patients and patients from an ethnic minority, plus mean patient age and calendar month. Since models of examination rate only analysed symptomatic patients, the proportion of symptomatic patients was excluded as a covariate. The proportion of MSM was only included in models for males. Complex cases were defined differently for males and females (definition S1). This is based on the definition used by Mohiuddin et al.[12] designed to identify patients requiring longer and/or more involved consultations.

Data for staff capacity was only available for the intervention site and was modelled as an uncontrolled interrupted time series spanning the duration of available denominator data: 1 January 2018 to 22 December 2019. The denominator could not be separated by gender, so this outcome was analysed for females and males combined, allowing two change points as per the respective intervention dates.

Sensitivity analyses were conducted by fitting generalised additive models to account for potential non-linearity of trends. All analyses were conducted with the SAS System for Windows, version 9.4 (SAS Institute Inc.). Models were fitted using the GENMOD and GAM procedures.

For peer review only - http://bmjopen.bmj.com/site/about/guidelines.xhtml

2.6 Economic analysis

Postal testing kit data were combined with EPR data to estimate the total number of episodes per week (including those with negative postal tests and no clinic attendance). For estimating the difference in the mean number of episodes per week i) negative postal test episodes were assigned to weeks pro rata with asymptomatic episodes that included clinic attendance, and ii) the combined post-intervention analysis used data for the first 43 weeks only. Episode costs were estimated using unit costs of diagnostic tests provided by the intervention site, and postal kit tests and staff time from the literature[12] inflated to 2021 values using a UK government GDP deflator[27]. Treatment costs were from the British National Formulary[28] (supplement Table S2). The cost of unreturned postal kits was allocated to episodes including a postal test result. Confidence intervals for differences in the number of episodes and cost per episode were calculated using the Normal approximation method.

Patient and public involvement

Three members of the public who had used the intervention site services as patients were involved in reviewing the proposed outcome measures and informed the study design.

3. Results

In the EHR intervention site data, 48,776 attendances for females and 34,413 for males were recorded during the study period, representing 32,482 and 22,073 episodes of care involving a clinic attendance, and 29,573 and 19,083 patients, respectively (Table 2). Patients were symptomatic in just over 20% of female attendances, and over 40% of male attendances. About 90% of female and 55% of male attendances were complex. Just over 30% of male attendances were by MSM.

For peer review only - http://bmjopen.bmj.com/site/about/guidelines.xhtml

3.1 Males

There was strong evidence of an adjusted step-increase for the intervention site relative to the control site (+89.1%, 95% confidence interval [CI] +37.1%, +160.6%, p<0.001) (Table 3 and Figure 1A). However, this was not observed in the sensitivity analysis allowing for non-linear trends (-16.6%, 95%CI -30.1%, -0.5%, p<0.001, supplement Table S3 and Figure S4A). This was followed by strong evidence of an adjusted downward change in post-intervention trend of -3.2% per week (95% CI -4.3%, -2.1%, p<0.001). The long-term result of these two effects was an overall decrease from 35-50 swabs per week, pre-intervention, to below 10 at the end of the study period, translating to 849 swabs avoided over the post-intervention period.

Time-to-notification increased by an estimated 3.6 days (95% CI 1.7, 5.5 days, p<0.001) at the time of the intervention, relative to controls, and a similar increase was observed in the sensitivity analysis. However, this was followed by an overall long-term decrease of -0.2 days of notification time per week (95% CI -0.3, -0.2 days, p<0.001) through the post-intervention period. That is, the pre-intervention weekly median of around eight to nine days dropped to around 2 days after the intervention had been in place for a year (Figure 1B, supplement Figure S4B).

We found no evidence of a meaningful change in rates of examinations or follow-up attendances associated with the intervention (Table 3, Figures 1C and 1D, supplement Figures S4C and S4D).

For peer review only - http://bmjopen.bmj.com/site/about/guidelines.xhtml

Table 2. Summary of population characteristics and outcomes by site, gender and time period based on EPR data.

	**Intervention site**		**Control site**	
	**Pre**	**Post**	**Pre**	**Post**
**MALES**				
Total attendances, n	17626	16787	11920	12085
Total episodes of care, n	11445	10628	7946	8021
Total patients, n	9932	9151	6271	6335
Symptomatic attendances, n (%)	7307 (41.5%)	7084 (42.2%)	4735 (39.7%)	4556 (37.7%)
Complex attendances, n (%)	9869 (56.0%)	9259 (55.2)%	4458 (37.4%)	4940 (40.9%)
Ethnic minority attendances, n (%)	2834 (16.1%)	3025 (18.0%)	7244 (60.8%)	7311 (60.5%)
MSM attendances, n(%)	5300 (30.1%)	5418 (32.3%)	2529 (21.2%)	2849 (23.6%)
Mean age, years	30.2	30.8	34.9	35.1
Urethral GC swabs per consultation	0.18	0.11	0.08	0.07
Median time-to-notification	10.90	6.73	4.51	4.95
Examinations per symptomatic attendance	0.76	0.67	0.64	0.60
Follow up attendances per episode	0.40	0.36	0.50	0.37
**FEMALES**				
Total attendances	28487	20289	20931	16910
Total episodes of care	18616	13866	13971	11660
Total patients	16779	12794	11799	9902
Symptomatic attendances	6312 (22.2%)	4929 (24.3%)	6860 (32.8%)	5561 (32.9%)
Complex attendances	26022 (91.3%)	18173 (89.6%)	12328 (58.9%)	11221 (66.4%)
Ethnic minority attendances	3979 (14.0%)	3067 (15.1%)	12647 (60.4%)	10107 (59.8%)
Mean age	25.1	25.8	29.8	30.4
Cervical GC swabs per consultation	0.20	0.04	0.03	0.03
Median time-to-notification (median, IQR)	10.58	3.52	4.90	5.32
Examinations per symptomatic attendance	0.73	0.70	0.58	0.60
Follow up attendances per episode	0.36	0.34	0.31	0.23

For peer review only - http://bmjopen.bmj.com/site/about/guidelines.xhtml

Table 3. Step change and slope change estimates from controlled interrupted time series models by outcome and sex. Change estimates are shown for the intervention and control sites and for the relative change for intervention site compared to the control site. All estimates are shown as percentage changes, except for time-to-notification where change estimates are given in days.

Outcome	Change at time of intervention			Trend change following intervention		
	Intervention site	*Control site*	*Intervention vs. control*	*Intervention site*	*Control site*	*Intervention vs. control*
**MALES – 12th November 2018**						
**1. Gonorrhoea culture swabs per consultation**	+6.5%	-43.7%	**+89.1% (+37.1%, +160.9%)**	-3.6%	-0.3%	**-3.2% (-4.3%, -2.1%)**
**2. Time-to-notification**	+2.2 days	+5.8 days	**+3.6 days (+1.7, +5.5)**	-0.19 days	+0.03 days	**-0.2 days (-0.3, -0.2)**
**3. Examinations per symptomatic attendance**	+3.6%	-1.6%	+5.4% (-7.5%, +20.0%)	-0.21%	-0.16%	-0.04% (-0.5%, +0.4%)
**4. Follow up attendances per episode**	-9.0%	-11.9%	+3.3% (-14.6%, +24.9%)	+0.23%	-0.001%	+0.30% (+0.31%, +0.96%)
**FEMALES – 29th May 2019**						
**1. Gonorrhoea culture swabs per consultation**	-38.7%	+3.6%	**-40.8% (-61.6%, -8.8%)**	-6.1%	-0.1%	**-6.1% (-7.8%, -4.5%)**
**2. Time-to-notification**	-2.5 days	-0.4 days	-2.1 (-4.5, 0.3) days	-0.11 days	-0.0001 days	**-0.1 (-0.2, -0.0) days**
**3. Examinations per symptomatic attendance**	-1.3%	-2.2%	+1.0% (-11.4%, +15.1%)	+0.09%	+0.03%	+0.1% (-0.4%, +0.5%)
**4. Follow up attendances per episode**	-8.2%	+2.7%	-10.6% (-27.6%, +10.3%)	-0.42%	+0.22%	-0.64% (-1.41%, +0.14%)

Note: Results for outcome 5 (staff capacity) reported separately in the text.

For peer review only - http://bmjopen.bmj.com/site/about/guidelines.xhtml

3.2 Females

For females, there was evidence of a decrease in the rate of gonorrhoea culture (GC) swabs: -40.8% (95% CI -61.6%, -8.8%, p=0.02) at the time of intervention, adjusted for control changes (Table 3, Figure 2A). This was followed by a decrease in trend through the post-intervention period, with an adjusted change of -6.1% per week (95% CI -7.8%, -4.5%, p<0.001). These changes represent a decrease from an estimated 0.22 swabs per consultation (over 30 swabs per week) immediately before the intervention to 0.14 immediately after (20 to 25 per week) and down to 0.01 at the end of the study period (less than five per week). Over the 43-week post-intervention period, an estimated 1542 swabs were avoided.

For time-to-notification, there was some evidence of a decrease of 2.1 days (95% CI -4.5, 0.3 days, p=0.08, Figure 2B) at the time of the intervention, adjusted for the control group. There was stronger evidence of a downward change in trend, estimated at -0.1 days per week (95% CI -0.20, -0.0 days, p=0.01) over the post-intervention period. These results were confirmed by the sensitivity analyses (Figure S5). To illustrate, the estimated median time-to-notification was eight to nine days just before the intervention, but a year later notification time was around one day.

For rates of examinations and follow up visits, we saw no evidence of intervention-related change (Table 3, Figures 2C and 2D).

[Figures 1 and 2 about here]

3.3 Staff capacity

The main analysis of staff capacity showed evidence of a trend change at the time of the male intervention (-1.1% per week, 95%CI -1.7%, -0.5%, p<0.001) and a step change at the time of the female intervention (+14.3%, 95% CI +3.4%, +26.3%, p=0.009) (Figure 3). However, the sensitivity analysis showed step changes in the opposite direction to the main analysis (supplement Figure S6), suggesting inconclusive evidence of change.

[Figure 3 about here]

3.4 Episodes and costs

For peer review only - http://bmjopen.bmj.com/site/about/guidelines.xhtml

Overall, the intervention site experienced a substantial increase in the weekly number of asymptomatic negative episodes managed via postal test kits, particularly for males, while both asymptomatic negative episodes seen in the clinic and symptomatic episodes decreased (Table 4). The mean cost per symptomatic episode increased by 9.2% to £69.04, while this was outweighed by a decrease of 13.5% to £26.23 for costs per asymptomatic episode, resulting in a combined decrease of 7.5%. The total cost per week decreased by 4.7%, largely due to the reduction in both the number and cost of episodes for asymptomatic females who attended the clinic.

For peer review only

For peer review only - http://bmjopen.bmj.com/site/about/guidelines.xhtml

Table 4. Intervention site pre- and post-intervention estimates of mean number of episodes per week, mean cost per episode and mean cost per week.

	Male					Female					Total				
	pre*	post*	% change	95% CI		pre*	post**	% change	95% CI		pre*	post**	% change	95% CI	
Mean number per week															
Asymptomatic	190.2	223.1	17.3	9.5	25.1	356.2	350.7	-1.5	-7.9	4.9	546.3	573.4	5.0	0.0	9.9
Postal negatives	70.5	111.5	58.2	48.7	67.7	96.3	124.9	29.7	22.3	37.0	166.8	236.2	41.6	35.7	47.4
Other^	119.6	111.5	-6.8	-13.7	0.2	259.9	225.9	-13.1	-19.2	-7.0	379.5	337.2	-11.1	-15.8	-6.5
Symptomatic	92.7	85.0	-8.3	-13.9	-2.7	84.4	77.8	-7.8	-14.6	-1.1	176.7	163.4	-7.5	-11.8	-3.2
Total	282.8	308.0	8.9	2.6	15.2	440.2	429.0	-2.5	-8.7	3.6	723.0	736.8	1.9	-2.5	6.3
Cost per episode (£)															
Asymptomatic	36.47	30.92	-15.2	-19.1	-11.3	27.04	24.23	-10.4	-13.3	-7.5	30.31	26.23	-13.5	-15.9	-11.0
Symptomatic	63.09	69.56	10.3	6.7	13.8	63.36	67.65	6.8	4.3	9.2	63.22	69.04	9.2	6.9	11.5
Total	45.19	41.58	-8.0	-10.8	-5.2	33.98	32.14	-5.4	-7.7	-3.1	38.36	35.47	-7.5	-9.3	-5.7
Cost per week (£)															
Resource															
Postal kit	382	592	55.0	45.9	64.1	629	848	34.8	27.4	42.2	1010	1437	42.3	36.5	48.1
In clinic diagnostic test	1962	1886	-3.9	-9.8	2.1	1452	1213	-16.5	-22.9	-10.1	3413	3155	-7.6	-11.9	-3.3
Consultation staff time	7497	7349	-2.0	-7.3	3.4	9396	8583	-8.7	-15.0	-2.3	16893	15959	-5.5	-9.5	-1.5
Treatment	3024	2896	-4.2	-13.1	4.6	3534	3085	-12.7	-20.3	-5.1	6558	6014	-8.3	-14.4	-2.2
Symptom status															
Asymptomatic	6949	6883	-1.0	-8.5	6.6	9673	8448	-12.7	-18.9	-6.4	16622	15392	-7.4	-12.3	-2.5
Symptomatic	5915	5840	-1.3	-7.5	5.0	5338	5280	-1.1	-8.7	6.6	11253	11174	-0.7	-5.5	4.0
Total	12865	12723	-1.1	-6.7	4.5	15010	13728	-8.5	-14.4	-2.6	27875	26565	-4.7	-8.6	-0.8

* based on 52 week period.
** based on 43 week period
^ includes positive postal test kits

For peer review only - http://bmjopen.bmj.com/site/about/guidelines.xhtml

4. Discussion

We have quantitatively evaluated the impact of a first-of-its-kind rapid STI testing on service delivery in an integrated SHS. Previous NPT assessments have taken a mathematical modelling approach[11-13]. The only other direct assessment of a chlamydia and gonorrhoea NPT in practice related to a rapid testing service model for asymptomatic patients without contraception provision[29]. This is the first study to quantify the effect of rapid chlamydia and gonorrhoea NPT on gonorrhoea culture swabs, time-to-notification, examinations, follow-up visits, staff capacity, and costs.

The substantial long term post-intervention decrease in the rate at which gonorrhoea swabs were sent for culture, for both males and females, was expected to some extent since patients with negative rapid tests in the new pathway avoided the need for cultures. Adams et al. [11] identified reduced gonorrhoea cultures as a key part of NPT-related cost reduction, although there has been no direct or simulated assessment of expected change in the number of cultures.

The trajectory of the decline in gonorrhoea swab rates following the intervention differed between males and females. The sensitivity analysis capturing non-linear trends suggested substantial decreases for males began more than six months after the intervention, with the lowest rates at one year post-intervention (Figure S4A). In contrast, rates for females appeared to respond to the intervention almost immediately and stabilise at a much lower level within about six months (Figure S5A). The differing implementation timeframes may reflect several barriers to implementation with the initial rollout for males, including providing training to a large group staff with varying timetables exacerbated by understaffing and budget cuts; variable application of eligibility criteria for the new service; and iterative revision of the new system and pathway[30]. There may also have been some just-in-case culture testing in the early stages until staff confidence in the system was established. With these issues largely resolved when the system was implemented for females, the transition appeared both smoother and faster, and this concurs with staff experience.

For peer review only - http://bmjopen.bmj.com/site/about/guidelines.xhtml

The rate of gonorrhoea swabs at the control site was relatively low throughout the period due to a conservative approach, appropriate to local prevalence, in which samples for cultures were only taken for NAAT-positive patients or those with high likelihood of infection. In contrast, standard practice at the intervention site in the pre-intervention period was to take cultures from all symptomatic patients with symptoms and/or signs potentially consistent with gonorrhoea and from potential contacts in addition to a NAAT as recommended in national guidelines.[31]

We estimated that median time-to-notification decreased from more than a week down to one or two days over the post-intervention period. However, given that it was not possible to separate out all rapid test results (e.g. notifications labelled “all negative”) and that we estimated real time rather than working days, the median time was likely lower, particularly for positive results. This is broadly consistent with findings from Whitlock et al. [29] who reported an average time-to-notification of 0.27 days for a new rapid NAAT testing service compared to 8.95 days for an off-site testing service for symptomatic patients.

The temporary increase in median time-to-notification for males after the intervention may result from the implementation challenges outlined above[30] in addition to a clinician-reported backlog in the early stages of transitioning to the new system. Once again, for males the transition appeared to take place over the full post-intervention period, while the equivalent period for females appeared faster with the lowest post-intervention sensitivity estimates occurring 21 weeks after the new system was implemented (supplement Figures S4B and S5B).

We observed no clear evidence of intervention-related changes in rates of examinations, follow up visits or staff capacity. All three were necessarily constructed from combinations of variables as there was no dedicated data field for each in the data. Although we did not detect a positive change, it is important to note that there was no evidence of a deleterious impact of the rapid testing service on any of these outcomes.

Staff capacity showed some evidence of intervention-related change, although the rate of patients seen per four-hour clinic was at similar levels at the end of the study period as at the start.

For peer review only - http://bmjopen.bmj.com/site/about/guidelines.xhtml

For asymptomatic patients, the provision of postal testing kits reduced the need for clinic attendance among those testing negative both for males and for females who did not have contraception needs. This combined with the introduction of shorter appointments more than likely increased staff capacity for this subgroup. Both also reduced the queueing time for walk-in clinics. Conversely, the reduced asymptomatic attendances meant that case-mix in the walk-in clinics became more demanding, with patients more likely to be symptomatic and/or complex[30], which may explain the lack of observed improvement in staff capacity during clinics. The lack of evidence for a capacity decrease through the implementation period despite a more demanding patient group and the growing numbers of asymptomatic patients being tested both suggest increased capacity of the SHS overall.

The change in management of asymptomatic clinical attendances, supported by the existing postal testing kit system, was a key component of the overall cost reduction following the introduction of the Panther technology, with decreases in both mean cost per asymptomatic episode (13.5%) and weekly asymptomatic costs (7.4%). Although the cost of symptomatic episodes increased, consistent with the reported increase in complexity of symptomatic patients in clinic, this was counteracted by a reduction in the number of weekly symptomatic attendances.

4.1 Strengths and limitations

We conducted a prospective real-time evaluation of a large integrated rapid STI service. We used a CITS framework with both a control site and confounder adjustment to estimate the effect of the intervention distinct from any background changes and independent of other time varying factors. This was bolstered by using a relatively long time series with good temporal resolution. The robustness of our analysis was supported by both sites using the same EPR system and the general consensus between main and sensitivity analyses.

In light of the target trial framework for natural experiments[32], our study was limited by being non-randomised, having only one control site, relying on the construction of certain outcomes

For peer review only - http://bmjopen.bmj.com/site/about/guidelines.xhtml

from multiple variables, and the impact of the Covid-19 pandemic on the follow up period for females. The unit costs were based on data provided by the intervention site and estimates from literature, and commissioners will need to assess their applicability to their locality.

4.2 Implications and conclusions

Several studies have suggested that NPT benefits include earlier diagnosis and treatment, reduced risk of sequelae and onward transmission, reduction in unnecessary treatments, earlier partner notification and reduced anxiety [10,29].

This quantitative assessment of the first UK implementation of rapid chlamydia and gonorrhoea testing within an integrated service revealed clear benefits, namely: reduced gonorrhoea culture swabs and shortened time-to-notification. These improvements, while maintaining activity at a lower overall cost, suggests that the introduction of clinic-based rapid testing had the intended impact, and this is in line with previous NPT modelling studies [10,11]. The qualitative evaluation of this rapid STI service also reported that patients valued faster results and avoiding unnecessary treatment, and that the better targeting of infection-specific treatment improved antimicrobial stewardship[30]. Although this was an evaluation of an integrated SHS providing contraception care in addition to testing, treatment and prevention services, it is likely the findings would be applicable to sexual health services which do not provide contraception care.

These results provide real-life evidence to support the benefits of a rapid testing service anticipated by modelling studies and strengthen the case for more widespread rollout in SHS.

For peer review only

For peer review only - http://bmjopen.bmj.com/site/about/guidelines.xhtml

Acknowledgements: The authors would like to thank Ed Hulse at Mill Systems for his indispensable assistance with the data extracts.

Funding statement: This research was funded by the National Institute for Health Research (NIHR) Applied Research Collaboration West (ARC West) at University Hospitals Bristol and Weston NHS Foundation Trust (core NIHR infrastructure funded: NIHR200181). The views expressed are those of the authors and not necessarily those of the NIHR or the Department of Health and Social Care. FdV is partly funded by the NIHR School for Public Health Research.

Competing interests: There are no competing interests to declare for any of the authors.

Data sharing statement: Anonymised individual-level data for this study comes from the electronic patient record system of the Unity Sexual Health and Croydon Sexual Health services (data controllers). Our data sharing agreement with the data controllers prohibits sharing data extracts outside of the University of Bristol research team. The data is available upon request from the data controllers.

Licence statement: I, the Submitting Author has the right to grant and does grant on behalf of all authors of the Work (as defined in the below author licence), an exclusive licence and/or a non-exclusive licence for contributions from authors who are: i) UK Crown employees; ii) where BMJ has agreed a CC-BY licence shall apply, and/or iii) in accordance with the terms applicable for US Federal Government officers or employees acting as part of their official duties; on a worldwide, perpetual, irrevocable, royalty-free basis to BMJ Publishing Group Ltd ("BMJ") its licensees and where the relevant Journal is co-owned by BMJ to the co-owners of the Journal, to publish the Work in BMJ Open and any other BMJ products and to exploit all rights, as set out in our licence.

The Submitting Author accepts and understands that any supply made under these terms is made by BMJ to the Submitting Author unless you are acting as an employee on behalf of your employer or a postgraduate student of an affiliated institution which is paying any applicable article publishing charge ("APC") for Open Access articles. Where the Submitting Author wishes to make the Work available on an Open Access basis (and intends to pay the relevant APC), the terms of reuse of such Open Access shall be governed by a Creative Commons licence – details of these licences and which Creative Commons licence will apply to this Work are set out in our licence referred to above.

Ethics approval: This study was approved by the Health Research Authority (South West) Research Ethics Committee, reference 18/SW/0090.

Contributorship statement: PH, MTR, FdV and HM conceptualized the evaluation; MTR and FdV are quantitative evaluation leads; WH and HM are health economic evaluation leads; SRW, JJ, RM and MTR acquired the analysis datasets; SRW conducted the time series analysis with supoprt from JJ, RM, MTR, PH and FdV; GM conducted the cost-effectiveness analysis with support from HM and WH; RG, MDC, MC, DP, PM, JS and JT advised on the study methodology, analysis and interpretation of results; SRW wrote the initial draft of the manuscript; all authors reviewed and edited the manuscript for content and approved the submission.

For peer review only - http://bmjopen.bmj.com/site/about/guidelines.xhtml

References

1. Public Health England, National STI surveillance data tables 2020 - Table 4. Available at: https://www.gov.uk/government/statistics/sexually-transmitted-infections-stis-annual-data-tables

2. Public Health England, National STI surveillance data tables 2020 - Table 3. Available at: https://www.gov.uk/government/statistics/sexually-transmitted-infections-stis-annual-data-tables

3. Mitchell H, Allen H, Sonubi T, Kuyumdzhieva G, Harb A, Shah A, Glancy M, Checchi M, Milbourn H, Folkard K, Mohammed H and contributors. Sexually transmitted infections and screening for chlamydia in England, 2019. September **2020**. London: Public Health England. Available at: https://assets.publishing.service.gov.uk/government/uploads/system/uploads/attachment_data/file/914249/STI_NCSP_report_2019.pdf

4. Price MJ, Ades AE, Soldan K, Welton NJ, Macleod J, Simms I, DeAngelis D, Turner KM, Horner PJ. The natural history of Chlamydia trachomatis infection in women: a multi-parameter evidence synthesis. Health Technol Assess, **2016**; 20: 1-250.

5. Ratna N, Sonubi T, Glancy M, Sun S, Harb A, Checchi M, Milbourn H, Dunn J, Sinka K, Folkard K, Mohammed H and contributors. Sexually transmitted infections and screening for chlamydia in England, 2020. September **2021**. London: Public Health England. Available at: https://assets.publishing.service.gov.uk/government/uploads/system/uploads/attachment_data/file/1015176/STI_NCSP_report_2020.pdf

6. Finch D, Bibby J, Elwell-Sutton T. Briefing: Taking our Health for Granted. October **2018**. London: The Health Foundation. Available online: https://www.health.org.uk/publications/taking-our-health-for-granted

7. White C. Sexual health services on the brink. Brit Med J, **2017**; 359: j5395.

8. Turner KME, Zienkiewicz AK, Syred J, Looker KJ, de Sa J, Brady M, Free C, Holdsworth G, Baraitser P. Web-Based Activity Within a Sexual Health Economy: Observational Study. J Med Internet Res, **2018**; 20:e74.

9. Turner KME, Looker KJ, Syred J, Zienkiewicz A, Baraitser P. Online testing for sexually transmitted infections: A whole systems approach to predicting value. PLoS ONE, **2019**; 14: e0212420. doi: 10.1371/journal.pone.0212420

10. Turner KME, Round J, Horner PJ, Macleod J, Goldenberg S, Deol A, Adams EJ. An early evaluation of clinical and economic costs and benefits of implementing point of care NAAT tests for Chlamydia trachomatis and Neisseria gonorrhoea in genitourinary medicine clinics in England. Sex Transm Infect, **2014**; 90: 104–111. doi: 10.1136/sextrans-2013-051147

11. Adams EJ, Ehrlich A, Turner KME, Shah K, Macleod J, Goldenberg S, Meray RK, Pearce V, Horner PJ. Mapping patient pathways and estimating resource use for point of care versus standard testing and treatment of chlamydia and gonorrhoea in genitourinary medicine clinics in the UK. BMJ Open, **2014**; 4: e005322. doi: 10.1136/bmjopen-2014-005322

12. Mohiuddin S, Gardiner R, Crofts M, Muir P, Steer J, Turner J, Wheeler H, Hollingworth P, Horner PJ. Modelling patient flows and resource use within a sexual health clinic through discrete event

For peer review only - http://bmjopen.bmj.com/site/about/guidelines.xhtml

simulation to inform service redesign. BMJ Open, **2020**;10: e037084. doi: 10.1136/bmjopen-2020-037084

13. Huntington SE, Burns RM, Harding-Esch E, Harvey MJ, Hill-Tout R, Fuller SS, Adams EJ, Sadiq ST. Modelling based evaluation of the costs, benefits and cost-effectiveness of multipathogen point-of-care tests for sexually transmitted infections in symptomatic genitourinary medicine clinic attendees. BMJ Open, **2018**; 8: e020394. doi: 10.1136/bmjopen-2017-020394

14. Miners A. Is nucleic acid amplification point-of-care testing for chlamydia and gonorrhoea cost-effective? Sex Transm Infect, **2014**; 90: 82.

15. Natoli L, Guy RJ, Shephard M, Causer L, Badman SG, Hengel B, Tangey A, Ward J, Coburn T, Anderson D, Kaldor J, Maher L. "I Do Feel Like a Scientist at Times": A Qualitative Study of the Acceptability of Molecular Point-Of-Care Testing for Chlamydia and Gonorrhoea to Primary Care Professionals in a Remote High STI Burden Setting. PloS ONE, **2016**; 10: e0145993. doi: 10.1371/journal.pone.0145993

16. Rompalo AM, Yu-Hsiang H, Hogan T, Barnes M, Jett-Goheen M, Huppert JS, Gaydon CA. Point-of-care tests for sexually transmissible infections: what do 'end users' want? Sex Health, **2013**; 10: 541-545. doi: 10.1071/SH13047

17. Horwood J, Ingle SM, Burton D, Woodman-Bailey A, Horner PJ, Jeal N. Sexual health risks, service use, and views of rapid point-of-care testing among men who have sex with men attending saunas: a cross-sectional survey. Int J STD AIDS, **2016**; 27: 273-280. doi: 10.1177/0956462415580504

18. Lorenc T, Marrero-Guillamón I, Aggleton P, Cooper C, Llewellyn A, Lehmann A, Lindsay C. Promoting the uptake of HIV testing among men who have sex with men: systematic review of effectiveness and cost-effectiveness. Sex Transm Infect, **2011**; 87: 272-278. doi: 10.1136/sti.2010.048280

19. Llewellyn CD, Sakal C, Lagarde M, Pollard A, Miners AH. Testing for sexually transmitted infections among students: a discrete choice experiment of service preferences. BMJ Open, **2013**; 3. doi: 10.1136/bmjopen-2013-003240

20. Llewellyn C, Pollard A, Miners A, Richardson D, Fisher M, Cairns J, Smith H. Understanding patient choices for attending sexually transmitted infection testing services: a qualitative study. Sex Transm Infect, **2012**; 88: 504-509. doi: 10.1136/sextrans-2011-050344

21. Hologic Inc. Panther® System. https://www.hologic.com/hologic-products/diagnostic-solutions/panther-scalable-solutions/panther-system

22. Department of Health and Social Care, Public Health England. Integrated Sexual Health Services: A suggested national service specification. August 2018: London. Available at: https://assets.publishing.service.gov.uk/government/uploads/system/uploads/attachment_data/file/731140/integrated-sexual-health-services-specification.pdf

23. Nicholls JE, Turner KME, North P, Ferguson R, May MT, Gough K, Macleod J, Muir P, Horner PJ. Cross-sectional study to evaluate Trichomonas vaginalis positivity in women tested for Neisseria gonorrhoeae and Chlamydia trachomatis, attending genitourinary medicine and primary care clinics in Bristol, South West England. Sex Transm Infect. **2018**;94: 93-99. doi: 10.1136/sextrans-2016-052942.

For peer review only - http://bmjopen.bmj.com/site/about/guidelines.xhtml

24. Mill Systems Limited. https://www.millsystems.com/millcare/

25. Simonton DK. Cross-sectional time series experiments: Some suggested statistical analyses. Psychol Bull, **1977**; 84: 489-502.

26. Bernal JL, Cummins S, Gasparrini A. The use of controls in interrupted time series studies of public health interventions. Int J Epi, **2018**; 47: 2082–2093. doi: 10.1093/ije/dyy135.

27. HM Treasury. GDP deflators at market prices, and money GDP December 2021 (Quarterly National Accounts). Available at: https://www.gov.uk/government/statistics/gdp-deflators-at-market-prices-and-money-gdp-december-2021-quarterly-national-accounts

28. Joint Formulary Committee. British National Formulary. **2020**. Available at: http://www.medicinescomplete.com

29. Whitlock GG, Gibbons DC, Longford N, Harvey MJ, McOwan A, Adams EJ. Rapid testing and treatment for sexually transmitted infections improve patient care and yield public health benefits. Int J STD AIDS, **2018**; 29: 474–482.

30. Lorenc A, Kesten J, Brangan E, Horner PJ, Clarke M, Crofts M, Turner J, Muir P, Horwood J. What can be learnt from a qualitative evaluation of implementing a rapid sexual health testing, diagnosis and treatment service? BMJ Open, **2021**; 11: e050109. doi: 10.1136/bmjopen-2021-050109

31. Fifer H, Saunders J, Soni S, Tariq Sadiq S, FitzGerald M. 2018 UK national guideline for the management of infection with *Neisseria gonorrhoeae*. Int J STD AIDS, **2019**; 31: 4-15.

32. de Vocht F, Katikireddi SV, McQuire C, Tilling K, Hickman M, Craig P. Conceptualising natural and quasi experiments in public health. BMC Med Res Methodol, **2021**; 21: 32. doi:10.1186/s12874-021-01224-x

For peer review only

For peer review only - http://bmjopen.bmj.com/site/about/guidelines.xhtml

Figure legends

Figure 1. Modelled outcome estimates for males. 'Panther' indicates the intervention-date representing the first week the Panther system was implemented for the male pathway: 12 November 2018.

Figure 2. Modelled outcome estimates for females. 'Panther for females' indicates the intervention-date representing the first week the Panther system was implemented for the female pathway: 29 May 2019.

Figure 3. Modelled estimates of staff capacity for males and females combined.

For peer review only

For peer review only - http://bmjopen.bmj.com/site/about/guidelines.xhtml

Figure 1. Modelled outcome estimates for males. 'Panther' indicates the intervention-date representing the first week the Panther system was implemented for the male pathway: 12 November 2018.

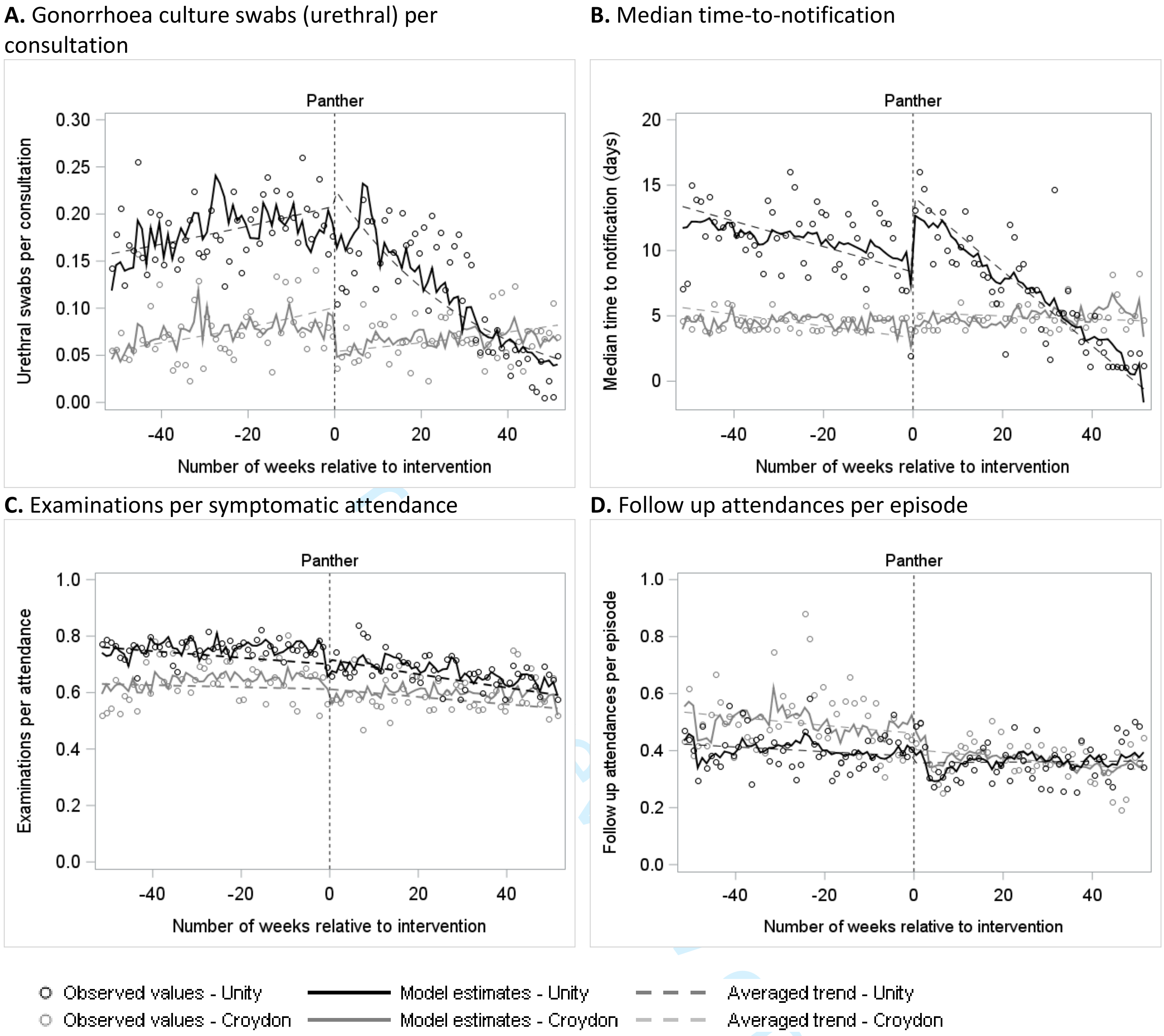

Figure 2. Modelled outcome estimates for females. 'Panther for females' indicates the intervention-date representing the first week the Panther system was implemented for the female pathway: 29 May 2019.

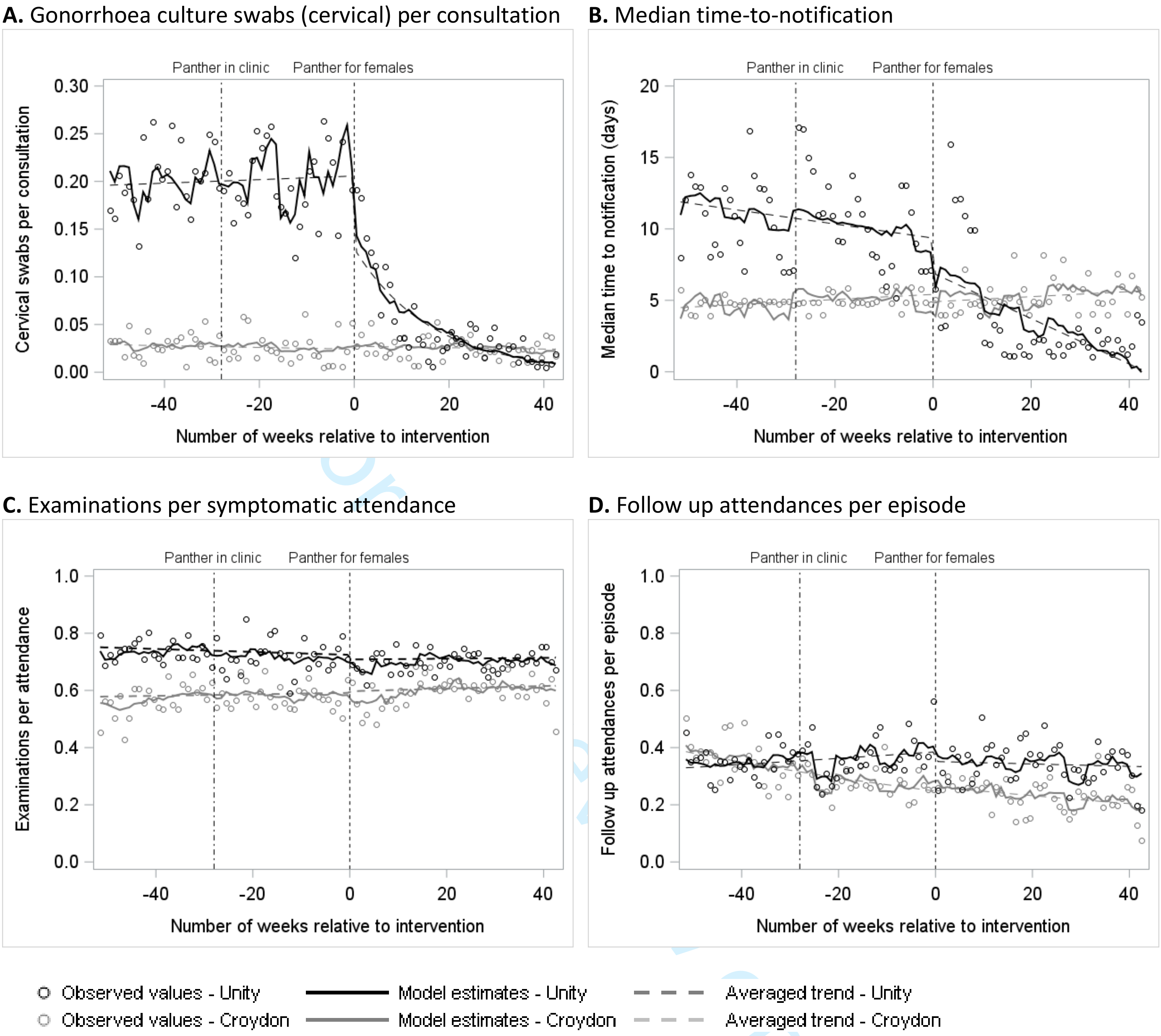

Figure 3. Modelled estimates of staff capacity for males and females combined.

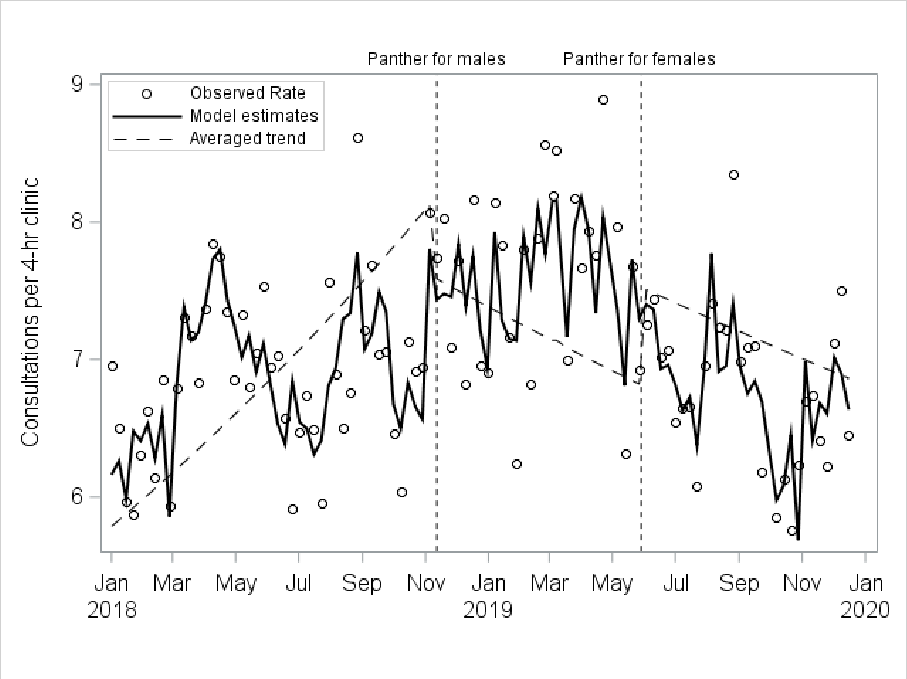

For peer review only

Supplementary material

The impact of rapid near-patient STI testing on service delivery outcomes in an integrated sexual health service in the United Kingdom: a controlled interrupted time series study

Scott R Walter, Joni Jackson, Gareth Myring, et al.

Contents:

Rapid STI testing details

Figure S1. Male treatment pathway before (usual care pathway) and after (rapid pathway) implementation of the Panther rapid results system.

Figure S2. Overview of female rapid treatment pathway for asymptomatic and symptomatic patients before (usual care pathway) and after (rapid pathway) implementation of the Panther rapid results system.

Table S1. Definition of examination of any type based on a combination of two examination-related variables.

Figure S3. Diagrams to illustrate controlled interrupted time series variables for estimating A) changes at the time of intervention and B) changes in trends.

Table S2. Unit costs.

Table S3. Intervention-related model estimates for females and males from sensitivity analyses using generalised additive models.

Figure S4. Modelled outcome estimates for males based on sensitivity analyses using generalised additive models.

Figure S5. Modelled outcome estimates for females based on sensitivity analyses using generalised additive models.

Figure S6. Modelled estimates of staff capacity for males and females combined.

Definition of complex cases

Definition of ethnic minority

For peer review only

Rapid STI testing details

Male urine samples, self-taken vaginal swabs, and clinician-taken endocervical swabs, rectal swabs and throat swabs were collected into the appropriate Aptima Collection kits (Hologic) and tested in the satellite laboratory at the intervention site for chlamydia and gonorrhoea using the Aptima Combo 2 assay (Hologic). Vaginal and endocervical samples were tested for Trichomonas using the Aptima *Trichomonas vaginalis* assay (Hologic). Reactive chlamydia NAAT results were not confirmed and were reported as positive, as internal audit has demonstrated that the result of the Aptima Combo 2 NAAT has a high concordance with Aptima CT NAAT when used to confirm a reactive chlamydia Aptima Combo 2 result[S1].

A reactive gonorrhoea Aptima Combo 2 result was reported as reactive and sent to the main laboratory (UKHSA South West Regional Laboratory) for confirmation using the Aptima GC NAAT. As the UKHSA South West Regional Laboratory have a > 90% positive predictive value (low risk of false positives) for a reactive gonorrhoea Aptima Combo 2 result, such patients were managed by clinicians as though they were gonorrhoea-positive which is consistent with the British Association for Sexual Health and HIV (BASHH) national guideline[S2] but informed that there was a small chance it could be a false positive result pending the confirmatory test result, which was usually available within 2 working days. All assays were performed using the automated Panther instrument (Hologic). Quality control measures in both laboratories included weekly testing of the Amplirun Total CT/NG/TV/MGE Control (Vircell, testing of NEQAS *Chlamydia trachomatis* & *Neisseria gonorrhoeae* external quality assessment (EQA) panels three times per annum and testing of Quality Control in Molecular Diagnostics *C. trachomatis* DNA and *N. gonorrhoeae* DNA EQA panels twice per annum.)

S1. Public Health England. UK Standards for Microbiology Investigations: *Chlamydia trachomatis* infection – testing by Nucleic Acid Amplification Tests (NAAT). V 37, issue 4, 9th January 2017. Available at: https://assets.publishing.service.gov.uk/government/uploads/system/uploads/attachment_data/file/583847/V_37i4.pdf)

S2. Fifer H, Saunders J, Soni S, Sadiq ST, FitzGerald M. UK national guideline for the management of infection with Neisseria gonorrhoeae. Int J STD AIDS, **2020**; 31(1): 4-15. doi: 10.1177/0956462419886775.

For peer review only

Figure S1. Male treatment pathway before (usual care pathway) and after (rapid pathway) implementation of the Panther rapid results system. Reproduced from: Lorenc A, Kesten J, Brangan E, Horner PJ, Clarke M, Crofts M, Turner J, Muir P, Horwood J. What can be learnt from a qualitative evaluation of implementing a rapid sexual health testing, diagnosis and treatment service? BMJ Open, 2021; 11: e050109. doi: 10.1136/bmjopen-2021-050109.

**Pathway stage**	**Usual care pathway**	**Rapid pathway**
**Reception**	- Walk-in patients' queue as clinic opens and appt are allocated on first-come-first-served basis	Patients are triaged to eligible for rapid STI service or not eligible.<br>• Eligible – Registered<br>• Non-eligible - continue along old pathway
**Clinical review**	Appt with clinician, full history taken.	Brief history taken at initial appt<br>MSM referred to Health Adviser<br>If follow up appt needed then full history taken at that time
**Sample testing**	Asymptomatic: Self-taken samples<br>Symptomatic: Clinician-taken samples for NAAT, GC culture and microscopy (as appropriate)	Self-taken NAAT testing **only** at initial appt<br>GC cultures taken **only** if GC positive on NAAT<br>Samples taken for microscopy only if NAAT negative and symptomatic
**Test results**	All test results available after approximately 10-14 days	Symptomatic: results available within the same day (triggering same/next day follow-up appt)<br>Asymptomatic: within 48 hours
**Treatment**	Patients treated syndromically prior to NAAT results.<br>Patient return for infection-specific treatment when test results are known (10-14 days)	Infection-specific treatment provided same/next day based on rapid test results
**Partner notification**	Started when positive test results returned (at 10-14 days)	Immediately commenced at same/next day follow-up appt

Figure S2. Overview of female rapid treatment pathway for asymptomatic and symptomatic patients before (usual care pathway) and after (rapid pathway) implementation of the Panther rapid results system. NAAT = Nucleic Acid Amplification Test GC = Gonorrhoea CT= Chlamydia, TV= *Trichomonas vaginalis*

Pathway stage	Usual care pathway	Rapid pathway: asymptomatic	Rapid pathway: symptomatic
**Reception**	Walk-in patients' queue as clinic opens and appointments are allocated on first-come-first-served basis.	Patients are triaged to rapid STI service or not:<br>● Asymptomatic and Eligible = Registered<br>● Asymptomatic and Non-eligible = continue along modified usual care pathway	Patients triaged to rapid STI service or not:<br>● Symptomatic and Eligible = Registered<br>● Symptomatic and non-eligible = modified usual care pathway (GC/CT and TV NAAT results <48 hrs) GC cultures only taken if GC NAAT positive or patient receives treatment for GC before results of GC NAAT available
↓	↓	↓	↓
**Clinical review**	Appt. with clinician, full history taken.	Brief history taken at initial appt.<br>If follow up appt. needed then full history taken at that time	History taken and no indications for visual inspection or pelvic examination required.<br>If follow up appt. needed then full history taken at that time
↓	↓	↓	↓
**Sample testing**	● Asymptomatic: Self-taken samples<br>● Symptomatic: Clinician-taken samples for CT/GC NAAT. If vaginal symptoms or pelvic pain then speculum exam and swabs taken for microscopy, TV and GC culture (as appropriate).	Self-taken NAAT testing ***only*** at initial appt<br>GC cultures taken ***only*** if GC positive on NAAT or if treatment as GC contact within 2 weeks of sexual intercourse with contact	Self-taken:<br>● Vaginal CT/GC/TV NAAT<br>● Vaginal swab for Gram staining<br>GC cultures taken ***only*** if GC positive on NAAT or if treatment as GC contact within 2 weeks of sexual intercourse with contact.
↓	↓	↓	↓
**Test results**	All test results available after approximately 10-14 days.	Results available within 48 hours.	Results of vaginal microscopy available in 20 mins and patient is then reviewed by clinician CT/GCTV NAAT results available within 48 hours
↓	↓	↓	↓
**Treatment**	Patients treated syndromically prior to NAAT results.<br>Patients return for infection-specific treatment when test results are known (10-14 days).	Infection-specific treatment provided same/next day based on rapid test results.	Infection-specific treatment provided same/next day based on rapid test results.
↓	↓	↓	↓
**Partner notification**	Started when positive test results returned (at 10-14 days).	Immediately commenced at same/next day follow-up appt.	Immediately commenced at same/next day follow-up appt.

Table S1. Definition of examination of any type based on a combination of two examination-related variables.

Categorical exam variable	Free text exam variable	Define as exam – MALES	Define as exam - FEMALES
Yes	Notes indicating exam	Yes	Yes
Yes	Missing	Yes	Yes
No	Notes indicating exam	Yes	Yes
No	Notes indication NO exam		
No	Missing		
External only	Notes indicating exam	Yes	Yes
External only	Notes indication NO exam		
External only	Missing		Yes
Speculum and external	Notes indicating exam	Yes	Yes
Speculum and external	Notes indication NO exam		
Speculum and external	Missing		Yes
Missing	Notes indicating exam	Yes	Yes
Missing	Notes indication NO exam		
Missing	Missing		

Notes: The categorical exam variable was intended for use with female patients but was sometimes used for males.

For peer review only

Figure S3. Diagrams to illustrate controlled interrupted time series variables for estimating A) changes at the time of intervention and B) changes in trends.

A. Changes at the time of intervention

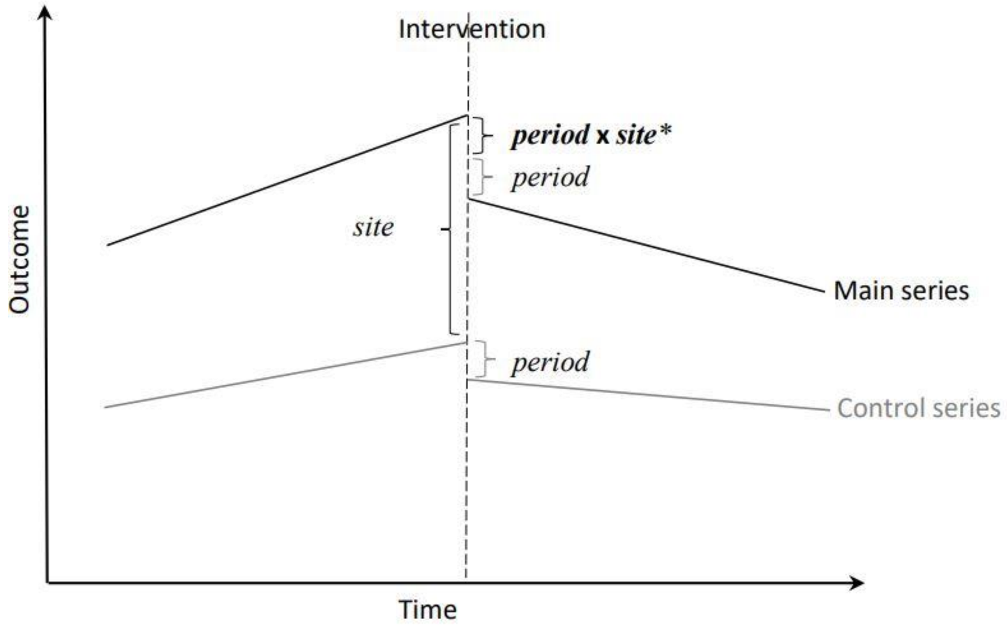

B. Trend changes

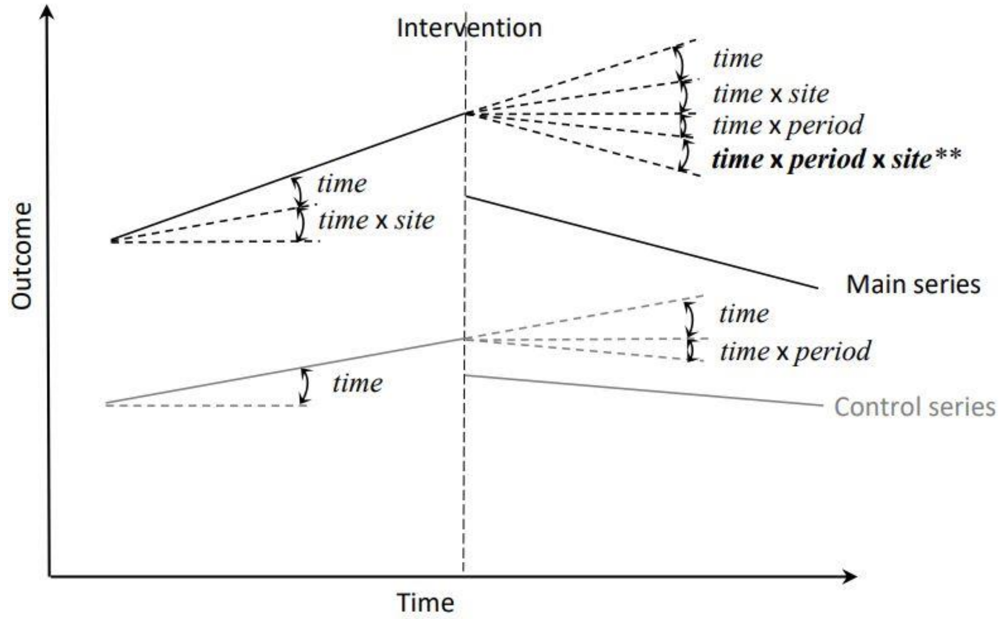

Note: *site* is a binary variable indicating either intervention or control sites; *period* is also binary indicating pre- or post-intervention periods; *time* is a continuous variable consecutively numbering each time unit (weeks in this study) with *time*=0 centred at the intervention

* *period* x *site* represents change in the intervention site at the time of intervention over and above any changes in the control site

** *time* x *period* x *site* represents change in trend for the intervention site over and above any trend changes in the control site

Table S2. Unit costs.

Unit costs of postal kit tests, and staff time[12] were from the literature and inflated to 2021 values using a UK government GDP deflator[26]. Local unit costs of diagnostic tests were provided by the intervention site. Treatment costs were from the British National Formulary[27].

Resource use	Unit cost
**Tests**	
Lab CT/GC test	£8.10
POCT CT/GC	£9.48
GC culture swab	£6.13
Male postal kit returned	£4.44
Male postal kit not returned	£3.61
Female postal kit returned	£4.08
Female postal kit not returned	£3.24
**Consultation staff time**	
Follow-up	£9.33
Male non-complex	£29.03
Male complex	£46.54
MSM complex	£42.97
Female non-complex	£29.03
Female complex	£52.26
**Treatment**	
13.5mg Levonorgestrel IUS	£69.22
6mg norelgestromin and 600micrograms ethinylestradiol	£19.51
Aciclovir 400 mg (tds for 5 Days)	£0.79
Aciclovir 400mg (bd for 6 months)	£17.64
Aciclovir 400mg (bd for 3 months)	£8.82
Aciclovir 800mg (tds for 2 days)	£0.59
Amoxicillin 250 mg tds for 5 days	£0.98
Amoxicillin 500mg	£1.01
Anusol Cream	£2.49
Anusol Ointment	£2.49
Anusol Suppositories	£1.74
Aqueous Cream BP 100g Tube	£0.77
Aqueous Cream BP 500g Tub	£3.85
Azithromycin 1g (2 x 500mg tablets)	£0.81
Azithromycin 1g (4 x 250mg capsules)	£1.24
Azithromycin 1g stat, then 500mg od for 2 days	£1.21
Azithromycin 1g stat, then 500mg od for 4 days	£2.42
Azithromycin 2g o stat	£1.62
Benzathine Benzylpenicillin 2.4 million units on day 0	£9.50
Benzathine benzylpenicillin 2.4 million units at day 7	£9.50
Benzathine benzylpenicillin 2.4 million units at day14	£9.50
Betamethasone Valerate 0.1% w/w Cream	£1.47
Betamethasone Valerate 0.1% w/w Ointment	£1.84
Betamethasone Valerate Ointment (Betnovate RD)	£1.84
Cefixime 400mg (2 x 200mg)	£26.46
Ceftriaxone 1g	£3.62
Ceftriaxone 500 mg (2 x 250mg vials)	£4.60
Chlorphenamine	£2.21
Cilest 63 tablet pack	£4.65

For peer review only

Ciprofloxacin 500mg (2 x 250mg)	£0.31
Clindamycin 300mg bd for 7 days	£17.84
Clindamycin phosphate vaginal cream	£10.86
Clobetasol Propinate (0.05% w/w) Cream (Dermovate)	£2.69
Clobetasol Propionate (0.05% w/w) Ointment	£2.69
Clobetasone Butyrate Cream (Eumovate)	£1.86
Clobetasone Butyrate Ointment (Eumovate)	£1.86
Clobetasone Butyrate, Calcium oxtertracycline & Nystatin Cream (Trimovate)	£12.45
Clotrimazole 100mg Pessary	£0.64
Clotrimazole 200mg Pessary	£1.14
Clotrimazole 500 mg Pessary	£6.99
Clotrimazole Cream 1%	£1.36
Co-Amoxiclav 250/125 (contains PENICILLIN)	£2.03
Co-amxoxiclav 500/125 (contains PENICILLIN)	£2.53
Crotamiton 10% w/w cream	£2.50
Dermol Lotion 500	£6.04
Desogestrel 75 micrograms	£2.26
Doxycycline 100mg (bd for 14 days)	£3.67
Doxycycline 100mg (bd for 21 days)	£5.51
Doxycycline 100mg (bd for 28 days)	£7.35
Doxycycline 100mg (bd for 7 days)	£2.26
Doxycycline 200mg bd for 4 weeks	£5.51
Emtricitabine 200mg & Tenofovir Disproxil 245mg	£106.00
Emtricitabine 200mg & Tenofovir Disproxil 245mg (3 days)	£10.60
Emulsifying Ointment	£4.82
Erythromycin 250 mg	£8.95
Estradiol 0.5g gel	£5.08
Estradiol 1.0mg gel	£5.85
Estradiol 10 micrograms vaginal tablet	£16.72
Femodene 63 tablet pack	£6.73
Flucloxacillin	£1.41
Fluconazole 150mg	£0.91
Fusidic acid cream	£1.92
GENTAMICIN 240mg for IM injection	£4.13
GYNAEFIX IUD	£27.11
Gardasil 0.5ml - First Dose	£86.50
Gardasil 0.5mls - Second Dose	£86.50
Gardasil 0.5mls - Third Dose	£86.50
Gedarel 20/150	£5.08
Gedarel 30/150	£5.08
Hepatitis A & B Combined Vaccine (adult)	£31.18
Hepatitis A Vaccine (2nd at 6 months)	£16.77
Hepatitis A vaccine Day 0	£16.77
Hepatitis B Vaccine (final at 6 months)	£12.20
Hepatitis B Vaccine - Dose 1 - 10mcg or 20mcg	£12.20
Hepatitis B Vaccine - Dose 2 - 10mcg or 20mcg	£12.20
Hepatitis B Vaccine - Dose 3 - 10mcg or 20mcg	£12.20
Hepatitis B Vaccine - Dose 4 - 10mcg or 20mcg	£12.20
Hepatitis B Vaccine - Extra Dose - 10mcg or 20 mcg	£12.20
Hepatitis B Vaccine 10 mcg 1 month	£12.20

For peer review only

Hepatitis B Vaccine 10 mcg 12 months	£12.20
Hepatitis B Vaccine Day 7	£12.20
Hepatits B Vaccine Day 21	£12.20
Hydro-Caine 6mls	£10.50
Hydrocortisone Cream 1%	£1.40
Hydrocortisone Ointment 1%	£1.59
Ibuprofen 200mg	£1.03
Imiquimod 5%	£48.60
Itraconazole 100mg	£3.29
Levonorgestral and Ethinylestradiol 150microgram/30microgram	£2.60
Levonorgestrel 1.5 mg	£3.65
Levonorgestrel 30 micrograms	£0.92
Levosert 52mgs IUS	£66.00
Lidocaine 4% w/w cream	£2.98
Lidocaine 5% m/m Ointment	£8.28
Lidocaine HCL 1% in 2 mls injection	£0.25
Lidocaine HCL 1% in 3.5 mls injection	£0.30
Lidocaine HCL 1% in 5 mls injection	£0.30
Lidocaine HCL 1% in 8mls for IM inj (with IM penicillin) second dose	£0.10
Lidocaine HCL 1% in 8mls for IM injection (with IM penicillin for syphilis)	£0.10
Lidocaine HCL 1% in 8mls for IM injection (with IM penicillin) third dose	£0.10
Lidocaine HCL 2% in 2 mls injection	£0.27
Lidocaine HCL 2% in 5 mls injection	£0.32
Lignocaine 2% Gel	£2.99
Loestrin 20 63 Tablet Pack	£1.99
Loestrin 30 63 Tablet Pack	£1.99
Logynon	£2.60
Marvelon 63 Tablet Pack	£7.10
Mebendazole 100mg	£2.66
Medroxyprogesterone Acetate 104mg in 0.65mls sub cutaneous	£6.90
Medroxyprogesterone Acetate 150mg in 1ml	£6.01
Mefenamic Acid 250mg	£8.17
Mepivacaine Hydrochloride 3%	£0.44
Mepivicaine 3% in 2.2mls	£0.44
Mercilon 63 Tablet Pack	£8.44
Metronidazole 0.75% Vaginal Gel	£4.31
Metronidazole 2g stat dose (400 mg x 5)	£0.52
Metronidazole 400mg (bd for 5 days)	£1.03
Metronidazole 400mg bd for 10 days	£2.07
Miconazole Nitrate 2%w/w, hydrocortisone 1%w/w Cream (Daktocourt)	£2.49
Miconazole Nitrate Cream 20mg/g (Gyno-Daktarin)	£4.33
Miconazole nitrate 20mg per g	£4.33
Millinette 20/75	£5.41
Millinette 30/75	£4.12
Mini TT 380	£12.46
Mirena 52mg IUS	£88.00
Moxifloxacin 400mg od for 10 days	£19.08
Moxifloxacin 400mg od for 14 days	£26.71
Nexplanon 68mg implant	£83.43
Nitrofurantoin 50mg o qds 7 days	£5.08
Nitrofurantoin 50mg o qds for 3 days	£2.18

For peer review only

Nonoxinol-9	£11.00
Norethisterone 350 micrograms	£2.10
Norethisterone 350 micrograms 84 Tablet Pack	£2.10
Norimin 63 Tablet Pack	£2.28
Nova T 380	£15.20
Ofloxacin 200mg (one tablet twice daily for 14 days)	£12.54
Ofloxacin 200mg (one tablet twice daily for 7 days)	£6.27
Ofloxacin 200mg (two tablets twice daily for 14 days)	£25.09
Paediatric Hepatitis B Vaccine - Dose 1 - 10mcg	£12.20
Paediatric Hepatitis B Vaccine - Dose 2 - 10 mcg	£12.20
Paediatric Hepatitis B Vaccine - Dose 3 - 10 mcg	£12.20
Paediatric Hepatitis B Vaccine - Dose 4 - 10mcg	£12.20
Paracetamol 500mg	£0.86
Permethrin 5% w/w cream	£8.54
Podophyllotoxin 0.15% Cream	£17.83
Podophyllotoxin 0.5% Solution	£14.49
Raltegravir 400 mg bd for 3 days	£47.14
T- Safe 380A QL	£10.55
TT 380 Slimline	£12.46
Terbinafine Hydrochloride 1% Cream	£2.39
Trimethoprim 200mg	£1.16
Ulipristal Acetate 30mg	£14.05
Xylocaine 1% with adrenaline 1 :200,000	£1.77

For peer review only

Table S3. Intervention-related model estimates for females and males from sensitivity analyses using generalised additive models.

Outcome	Change at time of intervention (95% CI)	P-value for post-panther non-linearity of intervention site data
**MALES – 12th November 2018**		
Gonorrhoea culture swabs per consultation	-16.6% (-30.1%, -0.5%)	<0.001
Time to notification	+0.4 days (+0.27, +0.71)	0.03
**FEMALES – 29th May 2019**		
Gonorrhoea culture swabs per consultation	-11.1% (-29.8%, +12.6%)	<0.001
Time to notification	-0.16 days (-0.42%, +0.10)	<0.001

For peer review only

Figure S4. Modelled outcome estimates for males based on sensitivity analyses using generalised additive models. Both the overall time trend and the post-panther intervention site trend were estimated as splines with three degrees of freedom. All other covariates treated as in the main analysis.

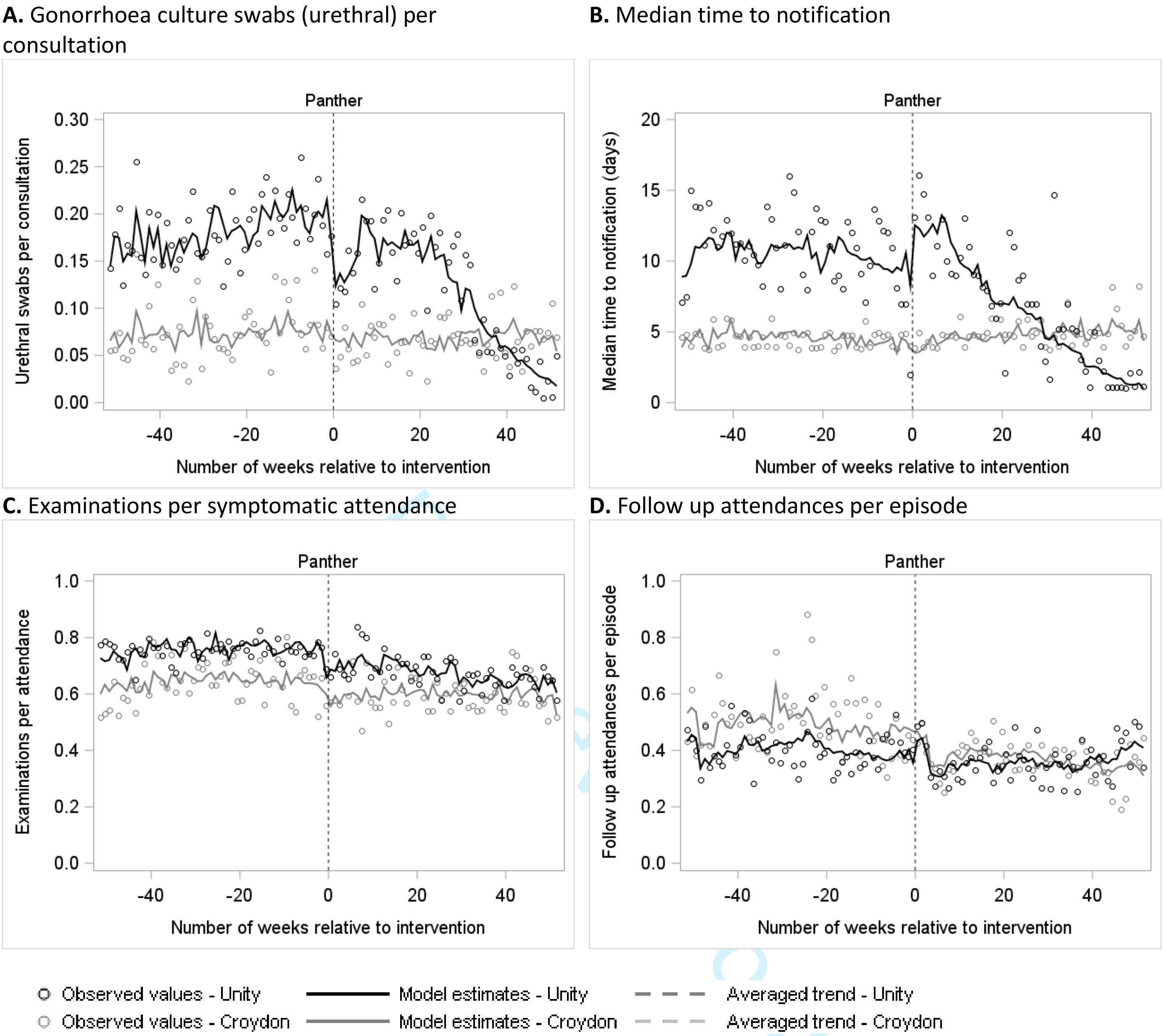

Figure S5. Modelled outcome estimates for females based on sensitivity analyses using generalised additive models. Both the overall time trend and the post-panther intervention site trend were estimated as splines with three degrees of freedom. All other covariates treated as in the main analysis.

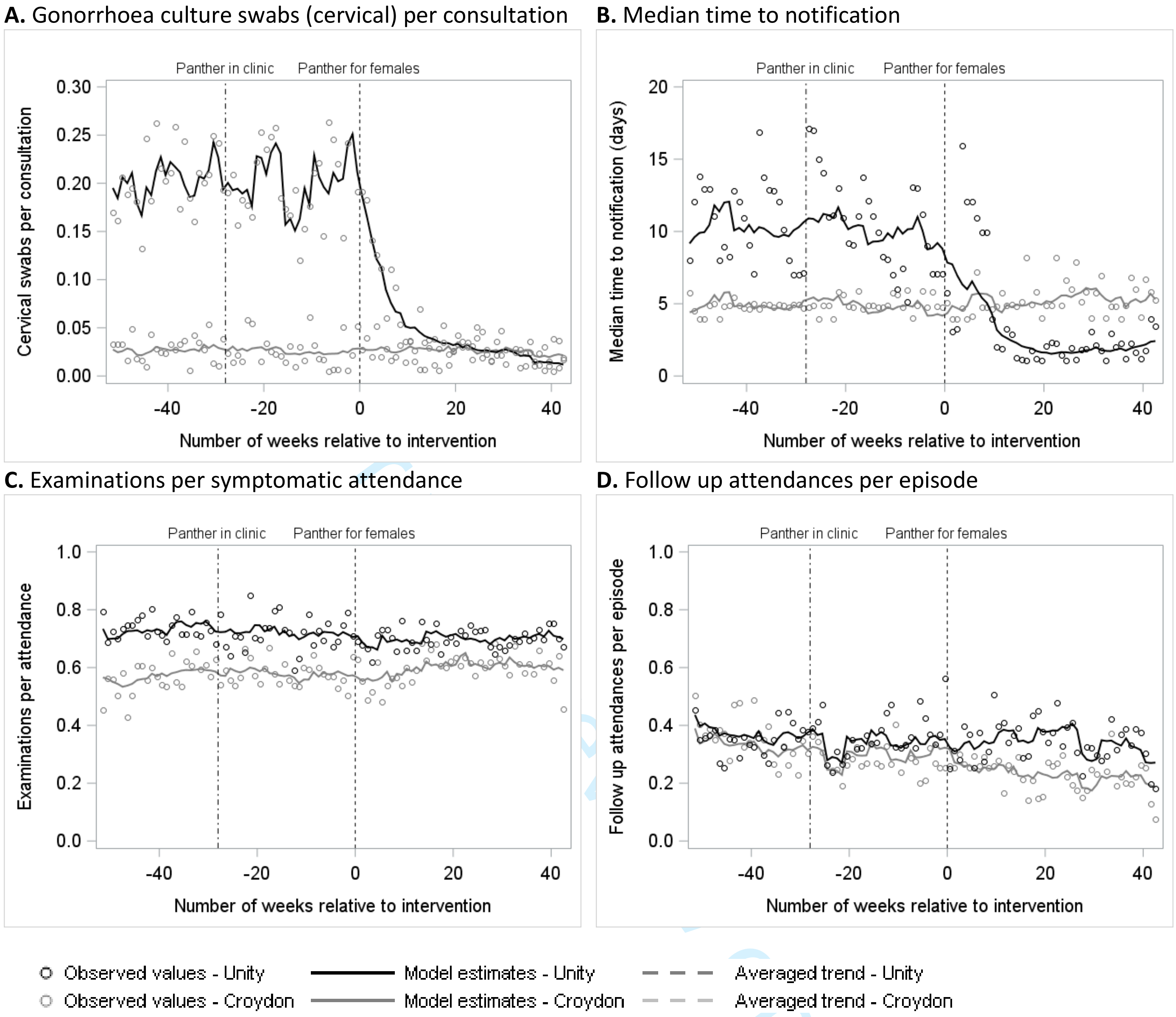

Figure S6. Modelled estimates of staff capacity for males and females combined. Time trends modelled with splines to allow for non-linearity. All other covariates treated as in the main analysis.

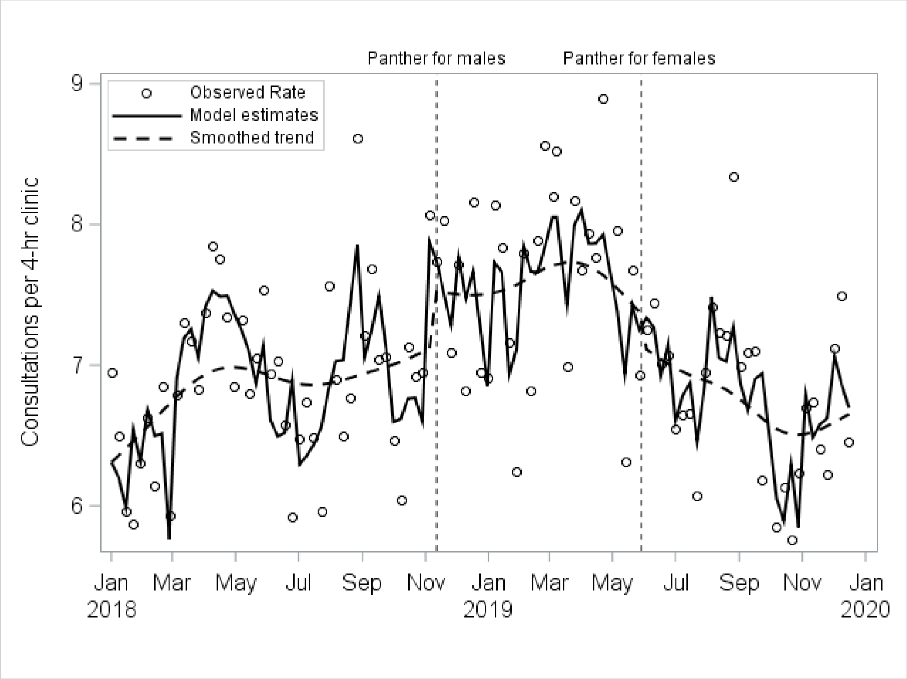

For peer review only

Definition of complex cases

Criteria for all patients:

a. Patients under 18 years of age
b. Have been/are currently exposed to child sexual exploitation, domestic violence, sexual assault
c. Has a current record of substance misuse
d. Has a current diagnosis of syphilis
e. Has current multiple diagnoses clinical diagnoses (GUMCAD coding B &/or C)
f. Has a history of/current diagnosis of genital herpes or had a swab taken for genital herpes
g. Has had post exposure prophylaxis after sexual exposure to HIV (PEPSE)
h. Needed an interpreter/use of translation service
i. Has current diagnosis of D2B on GUMCAD

Additional criteria for females:

i. Receive contraceptive care
ii. experienced pelvic pain, dyspareunia or post coital bleeding
iii. are pregnant
iv. experienced female genital mutilation.

Additional criteria for males:

v. are bisexual
vi. has sex with men
vii. Experienced testicular pain
viii. has a history/current record of chronic pelvic syndrome

For peer review only

Definition of an ethnic minority

This is self-reported at patient registration using the nationally-defined categories embedded within the patient record system.

Categories included in our definition are:

African

Caribbean

Any other black background

White and black Caribbean

White and black African

Indian

Pakistani

Bangladeshi

Any other Asian background

White and Asian

Chinese

Any other mixed background

Any other ethnic group

For peer review only

The RECORD statement – checklist of items, extended from the STROBE statement, that should be reported in observational studies using routinely collected health data.

	Item No.	STROBE items	Location in manuscript where items are reported	RECORD items	Location in manuscript where items are reported
**Title and abstract**					
	1	(a) Indicate the study's design with a commonly used term in the title or the abstract (b) Provide in the abstract an informative and balanced summary of what was done and what was found	(a) p.1<br>(b) p.2	RECORD 1.1: The type of data used should be specified in the title or abstract. When possible, the name of the databases used should be included.<br><br>RECORD 1.2: If applicable, the geographic region and timeframe within which the study took place should be reported in the title or abstract.<br><br>RECORD 1.3: If linkage between databases was conducted for the study, this should be clearly stated in the title or abstract.	1.1, abstract p.2<br><br>1.2, abstract p.2<br><br>N/A
**Introduction**					
Background rationale	2	Explain the scientific background and rationale for the investigation being reported			Introduction pp.4-5
Objectives	3	State specific objectives, including any prespecified hypotheses			End of introduction p.5
**Methods**					
Study Design	4	Present key elements of study design early in the paper			Section 2.1, p.5
Setting	5	Describe the setting, locations, and relevant dates, including periods of recruitment, exposure, follow-up, and data collection			Methods pp.5-7

For peer review only

Participants	6	*(a) Cohort study* - Give the eligibility criteria, and the sources and methods of selection of participants. Describe methods of follow-up<br>*Case-control study* - Give the eligibility criteria, and the sources and methods of case ascertainment and control selection. Give the rationale for the choice of cases and controls<br>*Cross-sectional study* - Give the eligibility criteria, and the sources and methods of selection of participants<br><br>*(b) Cohort study* - For matched studies, give matching criteria and number of exposed and unexposed<br>*Case-control study* - For matched studies, give matching criteria and the number of controls per case		RECORD 6.1: The methods of study population selection (such as codes or algorithms used to identify subjects) should be listed in detail. If this is not possible, an explanation should be provided.<br><br>RECORD 6.2: Any validation studies of the codes or algorithms used to select the population should be referenced. If validation was conducted for this study and not published elsewhere, detailed methods and results should be provided.<br><br>RECORD 6.3: If the study involved linkage of databases, consider use of a flow diagram or other graphical display to demonstrate the data linkage process, including the number of individuals with linked data at each stage.	Table 1<br><br>N/A<br><br>N/A
Variables	7	Clearly define all outcomes, exposures, predictors, potential confounders, and effect modifiers. Give diagnostic criteria, if applicable.		RECORD 7.1: A complete list of codes and algorithms used to classify exposures, outcomes, confounders, and effect modifiers should be provided. If these cannot be reported, an explanation should be provided.	Outcomes – table 1<br>Confounders – section 2.5, pp.7-8
Data sources/ measurement	8	For each variable of interest, give sources of data and details of methods of assessment (measurement).<br>Describe comparability of assessment methods if there is more than one group			Sections 2.4 & 2.5, pp.6-9

For peer review only

Bias	9	Describe any efforts to address potential sources of bias			Section 2.5, pp.7-8
Study size	10	Explain how the study size was arrived at			Section 2.4, p.6
Quantitative variables	11	Explain how quantitative variables were handled in the analyses. If applicable, describe which groupings were chosen, and why			Sections 2.5 and 2.6, pp.7-9 Supplement Table S1
Statistical methods	12	(a) Describe all statistical methods, including those used to control for confounding<br>(b) Describe any methods used to examine subgroups and interactions<br>(c) Explain how missing data were addressed<br>(d) *Cohort study* - If applicable, explain how loss to follow-up was addressed<br>*Case-control study* - If applicable, explain how matching of cases and controls was addressed<br>*Cross-sectional study* - If applicable, describe analytical methods taking account of sampling strategy<br>(e) Describe any sensitivity analyses			Sections 2.5 and 2.6, pp.7-9
Data access and cleaning methods		..		RECORD 12.1: Authors should describe the extent to which the investigators had access to the database population used to create the study population.	12.1: Section 2.4, p.6<br><br>12.2: Section 2.4, p.7

For peer review only

				RECORD 12.2: Authors should provide information on the data cleaning methods used in the study.	
Linkage		..		RECORD 12.3: State whether the study included person-level, institutional-level, or other data linkage across two or more databases. The methods of linkage and methods of linkage quality evaluation should be provided.	N/A
**Results**					
Participants	13	(a) Report the numbers of individuals at each stage of the study (*e.g.*, numbers potentially eligible, examined for eligibility, confirmed eligible, included in the study, completing follow-up, and analysed)<br>(b) Give reasons for non-participation at each stage.<br>(c) Consider use of a flow diagram		RECORD 13.1: Describe in detail the selection of the persons included in the study (*i.e.,* study population selection) including filtering based on data quality, data availability and linkage. The selection of included persons can be described in the text and/or by means of the study flow diagram.	Results, first paragraph, p.9
Descriptive data	14	(a) Give characteristics of study participants (*e.g.*, demographic, clinical, social) and information on exposures and potential confounders<br>(b) Indicate the number of participants with missing data for each variable of interest<br>(c) *Cohort study* - summarise follow-up time (*e.g.*, average and total amount)			Results, first paragraph p.9 and Table 2.
Outcome data	15	*Cohort study* - Report numbers of outcome events or summary measures over time<br>*Case-control study* - Report numbers in each exposure			Table 2

For peer review only

		category, or summary measures of exposure<br>*Cross-sectional study* - Report numbers of outcome events or summary measures			
Main results	16	(a) Give unadjusted estimates and, if applicable, confounder-adjusted estimates and their precision (e.g., 95% confidence interval). Make clear which confounders were adjusted for and why they were included<br>(b) Report category boundaries when continuous variables were categorized<br>(c) If relevant, consider translating estimates of relative risk into absolute risk for a meaningful time period			Sections 3.1-3.3 (pp.9-11) and Tables 3 & 4.
Other analyses	17	Report other analyses done—e.g., analyses of subgroups and interactions, and sensitivity analyses			Sections 3.1-3.3 (pp.9-11) and supplement table S3, figures S4-S6.
**Discussion**					
Key results	18	Summarise key results with reference to study objectives			pp.11-13
Limitations	19	Discuss limitations of the study, taking into account sources of potential bias or imprecision. Discuss both direction and magnitude of any potential bias		RECORD 19.1: Discuss the implications of using data that were not created or collected to answer the specific research question(s). Include discussion of misclassification bias, unmeasured confounding, missing data, and changing eligibility over time, as they pertain to the study being reported.	Section 4.1, pp13-14.
Interpretation	20	Give a cautious overall interpretation of results considering objectives,			Section 4.2, pp.14-15

For peer review only - http://bmjopen.bmj.com/site/about/guidelines.xhtml

		limitations, multiplicity of analyses, results from similar studies, and other relevant evidence			
Generalisability	21	Discuss the generalisability (external validity) of the study results			Section 4.2, pp.14-15
**Other Information**					
Funding	22	Give the source of funding and the role of the funders for the present study and, if applicable, for the original study on which the present article is based			p.16
Accessibility of protocol, raw data, and programming code		..		RECORD 22.1: Authors should provide information on how to access any supplemental information such as the study protocol, raw data, or programming code.	See data statement, p.16

*Reference: Benchimol EI, Smeeth L, Guttmann A, Harron K, Moher D, Petersen I, Sørensen HT, von Elm E, Langan SM, the RECORD Working Committee. The REporting of studies Conducted using Observational Routinely-collected health Data (RECORD) Statement. *PLoS Medicine* 2015; in press.

*Checklist is protected under Creative Commons Attribution (CC BY) license.

For peer review only
